# Effect of miR-183-5p on Cholestatic Liver Fibrosis by Regulating Fork Head Box Protein O1 Expression

**DOI:** 10.3389/fphys.2021.737313

**Published:** 2021-11-18

**Authors:** Yongxin Wang, Bin Chen, Chengcheng Xiao, Jiang Yu, Xiangyang Bu, Fengxing Jiang, Weijie Ding, Zhong Ge

**Affiliations:** ^1^Department of Hepatobiliary-Pancreatic Surgery, Qingdao Municipal Hospital, Qingdao University, Qingdao, China; ^2^Department of Urology, Qingdao Municipal Hospital, Qingdao University, Qingdao, China

**Keywords:** liver fibrosis, miR-183-5p, FOXO1, hepatic stellate cell, TGF-β pathway

## Abstract

Liver fibrosis is a common pathological feature of end-stage liver disease and has no effective treatment. MicroRNAs (miRNAs) have been found to modulate gene expression in liver disease. But the potential role of miRNA in hepatic fibrosis is still unclear. The objective of this research is to study the potential mechanism and biological function of miR-183-5p in liver fibrosis. In this study, we used high-throughput sequencing to find that miR-183-5p is upregulated in human fibrotic liver tissues. In addition, miR-183-5p was upregulated both in rat liver fibrosis tissue induced by bile-duct ligation (BDL) and activated LX-2 cells (human hepatic stellate cell line) according to the result of quantitative real-time PCR (RT-qPCR). Moreover, the inhibition of miR-183-5p alleviated liver fibrosis, decreased the fibrotic biomarker levels *in vitro* and *in vivo*, and led toLX-2 cell proliferation inhibition and, apoptosis induction. The result of dual-luciferase assay revealed that miR-183-5p suppressed fork head box protein O1 (FOXO1) expression by binding to its 3′UTR directly. Next, we used lentivirus to overexpress FOXO1 in LX-2 cells, and we found that overexpression of FOXO1 reversed the promotion of miR-183-5p on liver fibrosis, reducing the fibrotic biomarker levels inLX-2 cells, inhibitingLX-2 cell proliferation, and promoting apoptosis. Furthermore, overexpression of FOXO1 prevented the activation of the transforming growth factor (TGF)-β signaling pathway in TGF-β1-induced LX-2 cells according to the result of western blotting. In conclusion, the findings showed thatmiR-183-5p might act as a key regulator of liver fibrosis, and miR-183-5p could promote cholestatic liver fibrosis by inhibiting FOXO1 expression through the TGF-β signaling pathway. Thus, inhibition of miR-183-5pmay be a new way to prevent and improve liver fibrosis.

## Introduction

Hepatic fibrosis is highly prevalent in most chronic hepatic conditions, leading to cirrhosis, hepatic failure, cancer, and death (Ma et al., 2017). The fibrosis process includes stimulation of fiber production[extracellular matrix (ECM) synthesis] and regulation of fibrinolysis (ECM degradation) (Bataller and Brenner, 2005; Tsukada et al., 2006). It may be caused by hepatic lipid accumulation, such as non-alcoholic fatty liver disease, or it may be caused by poisoning or injury, such as excessive drinking or hepatitis, or bile acid accumulation, such as chronic cholestasis disease (Fernandez-Ramos et al., 2018). These cause hepatic stellate cells (HSCs) to be activated, which is the main mechanism leading to hepatic fibrosis (Geerts, 2001). α-smooth muscle actin (α-SMA) is a sign of HSC activation, and its expression level is positively correlated with the degree of liver fibrosis. Activated HSCs are the main precursor of fibroblasts, which secrete ECM components and stimulate cytokines (Higashi et al., 2017). The obstruction of intrahepatic or extra hepatic bile ducts causes cholestasis, which is characterized by bile duct reaction and extensive fibrosis (Desmet, 1995; Li and Crawford, 2004). HSCs are activated by several factors secreted by bile duct cells, including transforming growth factor (TGF)-β (Friedman, 2008; Wu et al., 2016). TGF-β stimulates the gene expression of ECM and decreases the degradation of ECM by downregulating matrix metalloproteinases (MMPs) and upregulating MMP-inhibitors (TIMPs) (Lurie et al., 2015). Although many studies have been performed on the cellular and molecular mechanisms involved in hepatic fibrosis (Elpek, 2014), the trigger mechanism for activation of HSC in liver fibrosis still remains to be further explored.

MicroRNAs (miRNAs) are approximately 22-nucleotide-long non-coding RNAs (Mohr and Mott, 2015). miRNAs suppress gene expression by inhibiting translation and degrading target mRNA (Lu and Rothenberg, 2018). In the liver, miRNA expression often correlates with non-alcoholic fatty liver disease, liver fibrosis, and hepatic carcinoma. Research shows that miRNAs play a pivotal role in the activation of HSC (Zhou et al., 2018). For instance, miRNA-29a inhibits BRD4 and reduces hepatic fibrosis in murine tissues by suppressing the activation of HSC (Huang Y. H. et al., 2019). Additionally, miR-455-3p reduces liver fibrosis and HSC activation by inhibiting the expression of HSF1 (Wei et al., 2019), and miR-199a-3p exacerbates hepatic fibrosis by inhibiting the expression of caveolin-2 and triggering the TGF-β pathway (Yang et al., 2020). However, the important microRNA related to liver fibrosis has not been revealed to a great extent.

In this work, we explored the modulatory mechanisms of miR-183-5p in hepatic fibrosis. First, miR-183-5p was found to be upregulated in human fibrotic liver tissue, activated LX-2 cells, and a fibrotic liver rat model. However, inhibition of miR-183-5p alleviated hepatic fibrosis, reduced the fibrotic biomarker levels *in vitro* and *in vivo*, which led to LX-2 cell proliferation inhibition and apoptosis induction. Furthermore, miR-183-5p was found to regulate liver fibrosis through the direct modulation of fork head box protein O1 (FOXO1) and overexpression of FOXO1 prevented TGF-β pathway in LX-2 cells. Our results reveal the therapeutic potential of miR-183-5p in hepatic fibrosis; it might be a potential target for slowing down or even reversing liver fibrosis.

## Materials and Methods

### Patient Samples

The fibrotic liver tissue samples were obtained from patients with hepatolithiasis who required liver resection at the Qingdao Municipal Hospital. Control hepatic tissue consisted of distal para-hemangioma healthy tissue from hepatic hemangioma cases requiring surgical resection. The clinical parameters of these patients are included in Table 1. All tissues were rapidly preserved at −80°C or 10% neutral-buffered formalin for clinical and pathological investigation. Samples of patients were obtained after informed consent. The research was approved by the Ethics Committee of Qingdao Municipal Hospital.

**TABLE 1:** Clinical characteristics of the patients with hepatolithiasis and hepatic hemangioma.

	Case 1	Case 2	Case 3	Case 4	Case 5	Case 6
**Gender**	**Male**	**Male**	**Female**	**Female**	**Male**	**Female**
Age	63	62	61	55	28	36
ALT (U/L)	116.22	342.04	25.62	15.20	22.60	17.56
AST (U/L)	90.32	244.80	28.00	19.25	30.25	29.00
ALP (U/L)	186.51	281.17	152.43	102.64	90.65	122.56
GGT (U/L)	300.97	480.03	118.86	27.00	35.82	15.45
TBIL (μmol/L)	37.20	50.40	13.70	36.60	18.56	20.25
Location of stone	Segments: II, III + CBD	Segments: II–IV +CBD	Segments: I–IV	None	None	None
Diameter of largest IHS (mm)	12	10	14	None	None	None
Location of hemangioma	None	None	None	Segments: VI, VII	Segments: II–IV	Segments: II, III
Removed parts	Segments: II, III	Segments: II–IV	Segments: I–IV	Segments: VI, VII	Segments: II–IV	Segments: II, III
liver fibrosis	Yes	Yes	Yes	None	None	None

*ALT, alanine aminotransferase; AST, aspartate aminotransferase; ALP, alkaline phosphatase; GGT, γ-glutamyltransferase; TBIL, total bilirubin; CBD, common bile duct; IHS, intrahepatic bile duct stones.*

### High-Throughput Sequencing

Normal liver tissues and hepatolithiasis-induced liver fibrosis tissues (*n* = 3) were analyzed using high-throughput sequencing. RNA database building, sequencing, and information analysis were performed at the Berry Genomics Co., Ltd, Beijing, China. EdgeR software (edgeR, RRID:SCR_012802) was used to screen differentially expressed miRNAs.

### Quantitative Real-Time PCR

TRIzol (Tiangen, Beijing, China) was used to extract total RNA from liver tissues or cells. The ABI 7500 Fast Real-time PCR system (7500 Real-Time PCR Software, RRID:SCR_014596) was used to perform quantitative real-time PCR (RT-qPCR). The 2-ΔΔCt method was used to calculate relative gene expression levels. *U6* and GAPDH served as internal references for miRNA and mRNA expression levels, respectively. The primers designed for RT-qPCR are shown in Table 2.

**TABLE 2:** Primer sequences for RT-qPCR.

Gene	Primer sequences	
miR-183-5p	F:5′-CGCGGTATGGCACTGGTAGA-3′	R:5′-AGTGCAGGGTCCGAGGTATTC-3
*FOXO1*	F: 5′-CAATGACCCCGCACGATTTC-3′	R:5′-CATGGAGGGCGGATTGGAA-3′
α-SMA	F: 5′-TCATGGTCGGTATGGGTCAG-3′	R:55′-CCGTGCTCGATAGGGTACTT-3′
*GAPDH*	F: 5′-GGCGTTCTCTTTGGAAAGGTGTTC-3′	R: 55′-GTACTCAGCGGCCAGCATCG-3′
*U6*	F:5′-GCTTCGGCAGCACATATACTAAAAT-3′	R:55′-CGCTTCACGAATTTGCGTGTCAT-3′

*F, forward primer; R, reverse primer.*

### Animal Model and Experimental Protocol

Male Wistar rats were procured from the Animal Center of Qingdao University. Research-purpose liver fibrosis was developed through BDL (14-day duration). In the SHAM group, a 2-cm incision was made in the midline of the upper abdomen, and the common bile duct was separated from the surrounding tissues. The rats in the BDL group were operated in the same way, and using 6–0 silk thread to doubly ligate the common bile duct (Wang et al., 2014). The rats in the BDL/sham groups were transfected with 10 mg/kg control antagomir or miR-183-5p antagomir (GenePharma Co., Ltd, Shanghai, China) via tail injection. Drugs were given from the second day after the operation, two times a week for 2 weeks (Wei et al., 2019). The rats were euthanized 2 weeks after the operation to collect blood and livers. The plasma was immediately separated by centrifugation and preserved at −20°C. The liver tissues were preserved at −80°C and within 4% paraformaldehyde for biochemical and pathological investigations.

### Histopathological Examination

The fixed liver tissue was cut into 4-μm-thick slices and then dehydrated using 70% ethanol. Dysmorphisms were identified by hematoxylin and eosin (H&E) or Masson’s trichrome staining. The latter was used to visualize collagen deposits and degree of fibrosis. Light microscopy (200× magnification) was used to capture photomicrographs. The Ishak scoring system was used to assess the fibrosis stages (F0 = no fibrosis to F6 = cirrhosis) (Ishak et al., 1995). Collagen deposits were quantitatively assessed for collagen volume fraction (CVF) in each sample slice using the following equation:


(1) C⁢V⁢F=c⁢o⁢l⁢l⁢a⁢g⁢e⁢n⁢a⁢r⁢e⁢a/t⁢o⁢t⁢a⁢l⁢a⁢r⁢e⁢a×100%.

### Biochemical Analysis

The levels of total bilirubin (TBIL), aspartate transaminase (AST), and alanine transaminase (ALT) in serum were measured by automatic chemiluminescence immunoassays (Roche Diagnostics).

### Immunohistochemistry

The 4-μm-thick slices after deparaffinization/hydration were blocked with hydrogen peroxide (3%) and then treated with 10% normal goat serum (Abcam Cat# ab7481, RRID:AB_2716553), followed by incubation with the anti-α-SMA antibody (1:500, Cell Signaling Technology Cat# 19245, RRID:AB_2734735), FOXO1 (1:1000, Cell Signaling Technology Cat# 2880, RRID:AB_2106495) at 4°C overnight. Then incubated the slices with horseradish peroxidase (HRP)-conjugated secondary antibody (1:500, Thermo Fisher Scientific Cat# 31466, RRID:AB_10960844). We used 3,3′-diaminobenzidine to stain the slides for coloration and microscopy (×200) were used to capture the images.

### Western Blotting Assay

Total proteome was collected from samples using RIPA lysis buffer (Aspen Biotechnology) containing protease inhibitors. Then using the BCA assay kit (Aspen Biotechnology) to quantify proteins. The following antibodies were used for incubating the membranes overnight: FOXO1 (1:1000, Cell Signaling Technology Cat# 2880, RRID:AB_2106495), α-SMA (1:1000, Cell Signaling Technology Cat# 19245, RRID:AB_2734735), collagen-I (1:1000, Cell Signaling Technology Cat# 91144, RRID:AB_2800169), tissue inhibitor of metalloproteinase 1 (TIMP-1) (1:2000, Thermo Fisher Scientific Cat# PA5-99559, RRID:AB_2818492), TGF-β1 (1:1000, Cell Signaling Technology Cat# 3711, RRID:AB_2063354), p-Smad2/3 (1:1000, Cell Signaling Technology Cat# 8828, RRID:AB_2631089), Smad2 (1:1000, Cell Signaling Technology Cat# 5339, RRID:AB_10626777), Smad3 (1:1000, Cell Signaling Technology Cat# 9523, RRID:AB_2193182), and GAPDH (1:1,000, Cell Signaling Technology Cat# 5174, RRID:AB_10622025). Thereafter, the membranes were incubated with HRP-conjugated secondary antibodies (1:2000; Cell Signaling Technology Cat# 7074, RRID:AB_2099233). The Odyssey Infrared Imaging System (LI-COR Biotechnology) was used to visualize Protein bands.

### Cell Culture and Transient Transfection

The human LX-2 cell line was purchased from BeNa Culture Collection (cat no. BNCC341818, Beijing, China). The cells were cultured in DMEM (Thermo Fisher Scientific, Inc) with 10% FBS (Thermo Fisher Scientific, Inc) at 37°C in 5% CO_2_. Using 10 ng/mL TGF-β1 (cat no. PHG9202, Thermo Fisher Scientific, Inc) to treat the cells for 48 h to induce target gene expression. Lipofectamine 2000 (Invitrogen, Shanghai, China) was used to transfect control antagomir, miR-183-5p antagomir, control agomir, miR-183-5p agomir, or FOXO1 lentivirus (GenePharma Co., Ltd, Shanghai, China) into LX-2 cells (Detailed procedure is available in the section “Supplementary Method”).

### Cell Viability Assay

The cell counting kit-8 (CCK-8) assay kit (cat no. CK04, Dojindo) was used for evaluatingLX-2 viability according to the manufacturer’s instructions. Absorbance was analyzed at 450 nm using the microplate reader.

### Flow Cytometry

According to the manufacturer’s instructions, LX-2 cells were identified using the Annexin-V-FITC Apoptosis Detection Kit (cat no. BB-4101-50T, Best bio). Flow cytometry was used to evaluate apoptosis.(Detailed procedure is available in the section “Supplementary Method”).

### Dual-Luciferase Reporter Assay

We applied the miRNA prediction websites miRbase (miRBase, RRID:SCR_003152), miRDB (miRDB, RRID:SCR_010848) and TargetScan (TargetScan, RRID:SCR_010845) to identify downstream genes and binding sites of miR-183-5p. The dual luciferase reporter gene detection was used to identify the targeting association of miR-183-5p/FOXO1. We constructed a wild-type (wt) pGL3-FOXO1 reporter plasmid containing the 3′ UTR of FOXO1 (putative binding location for miR-183-5p). Mutated (mut) pGL3-FOXO1, in which the potential miR-183-5p-binding sites were mutated, was also generated. Using Lipofectamine 2000 (Invitrogen, Shanghai, China), both the reporter plasmids were used to co-transfect LX-2 cells, with miR-183-5p agomir and control agomir (Wang et al., 2020). We detected and analyzed luciferase activity48 h after transfection.

### Statistical Analysis

We used the SPSS software version 22.0 (SPSS, RRID:SCR_002865) to perform statistical analyses. Datasets represent mean ± standard deviation (SD). All experiments were repeated three times. The *t* test was used for comparative analysis, whereas multigroup comparisons were performed using one-way analysis of variance. A probability value less than 0.05 indicates statistical significance.

## Results

### miR-183-5p Upregulation in Cholestatic Liver Fibrosis

**FIGURE 1:**
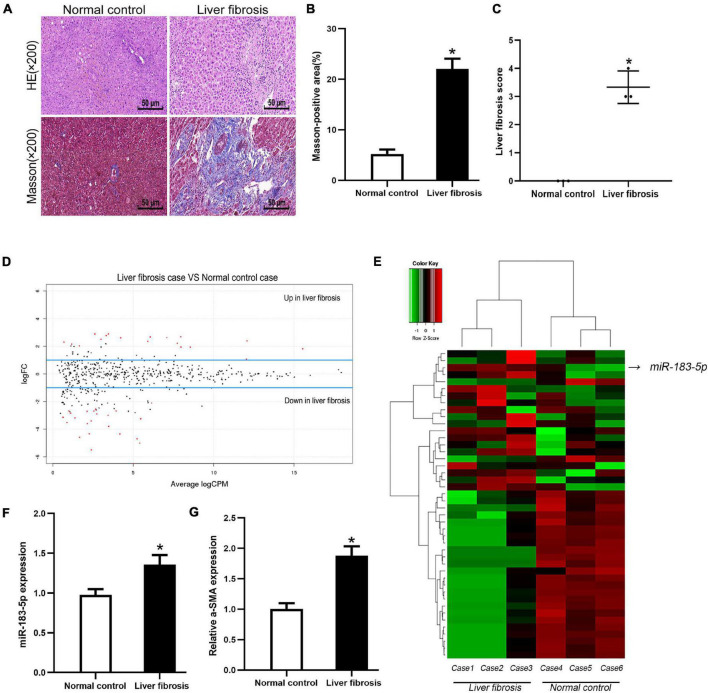
The high expression level of miR-183-5p in liver fibrosis tissues. **(A)** Representative images of H&E and Masson staining of liver fibrosis in human, 200×, Scale bar = 50 μm. **(B)** The quantification of Masson-positive fibrosis areas in human liver. **(C)** Statistical quantification analysis showed the liver fibrosis score based on the Ishak scoring system. **(D)** Aberrant expression miRNAs in liver fibrosis tissues were reflected by the MA plot. **(E)** The heatmap showed miR-183-5p was a higher-expression miRNA in liver fibrosis tissues (*n* = 3 per group). **(F)** The expression of miR-183-5p in human liver tissues was examined by RT-qPCR (*n* = 5 per group). **(G)** The expression of a-SMA in human liver tissues was examined by RT-qPCR (*n* = 5 per group). Data are expressed as the mean ± SD. *p < 0.05 vs. normal control group and independent *t*-test.

We screened three pairs of liver fibrosis and matched normal liver tissues. Differentially expressed miRNAs were detected and identified using high-throughput sequencing. First, H&E and Masson staining revealed increased fibrosis and collagen deposition in cholestatic liver fibrosis (Figures 1A–C). Differentially expressed miRNAs were screened, including 18 upregulated and 26 downregulated miRNAs according to the conditions of *p* < 0.05 and | log FC | > 1. Moreover, miR-183-5p was among the highly upregulated miRNAs (Figures 1D,E). To detect miR-183-5p expression in disease state, RT-qPCR was performed using both rescreened fibrotic liver (*n* = 5) and normal liver (*n* = 5) tissues, and miR-183-5p was found to be upregulated in the fibrotic liver (Figure 1F). Moreover, the expression of α-SMA was also upregulated in the fibrotic liver according to the result of RT-qPCR (Figure 1G).

### Inhibition of miR-183-5p Alleviated Liver Fibrosis After Bile-Duct Ligation

**FIGURE 2:**
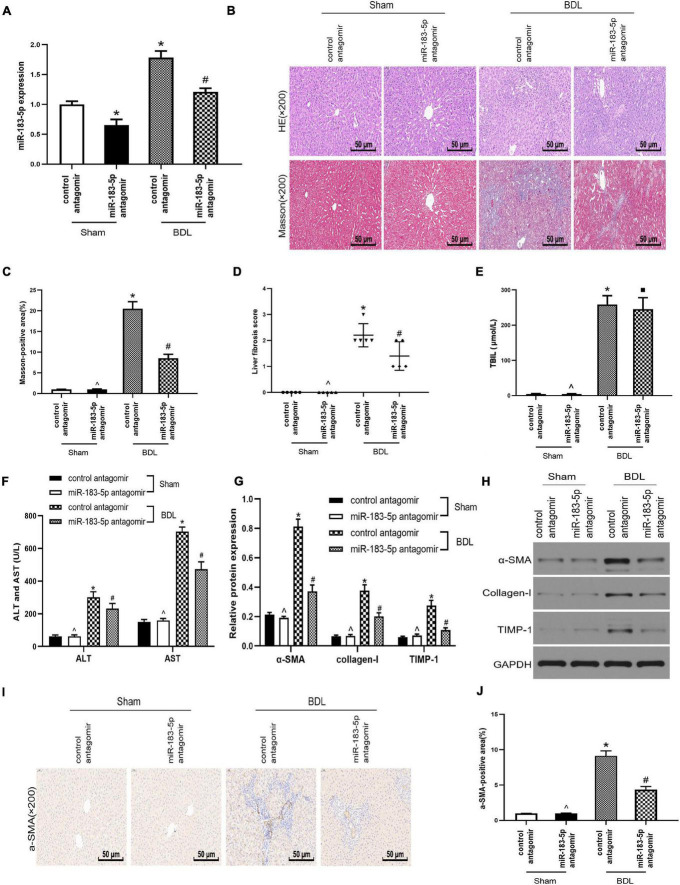
Inhibition of miR-183-5p alleviated liver fibrosis after bile duct ligation. **(A)** The expression of miR-183-5p was examined by RT-qPCR. **(B)** H&E and Masson staining of liver tissues in each group. 200×, Scale bar = 50 μm. **(C)** The quantification of Masson-positive fibrosis areas in each group. **(D)** Statistical quantification analysis showed the liver fibrosis score based on the Ishak scoring system. **(E)** TBIL level was measured by automatic chemiluminescence immunoassays (Roche Diagnostics). **(F)** ALT and AST levels was measured by automatic chemiluminescence immunoassays (Roche Diagnostics). **(G,H)** The protein levels of a-SMA, Collagen-I, and TIMP-1 were examined by western blotting. **(I)** Immunohistochemical staining of a-SMA in rat liver tissues. 200×, Scale bar = 50 μm. **(J)** The quantification of the a-SMA-positive area in each group. Data are expressed as the mean ± SD. *n* = 5 per group. *p < 0.05 vs. control antagomir Sham group, #*P* < 0.05 vs. control antagomir BDL group, ^*p* > 0.05 vs. control antagomir Sham group, ■ *P* > 0.05 vs. control antagomir BDL group, one-way ANOVA.

As miR-183-5p was upregulated in cholestatic liver fibrosis, it prompted us to further study the relationship between miR-183-5p and liver fibrosis. We treated Wistar rats with BDL for 2 weeks and established an experimental liver fibrosis model. RT-qPCR showed that miR-183-5p was upregulated in the hepatic tissue of rats in the BDL group, whereas application of the miR-183-5p antagomir downregulated the level of miR-183-5p (Figure 2A). H&E and Masson staining showed that miR-183-5p antagomir alleviated BDL-induced liver fibrosis (Figures 2B–D). Meanwhile the levels of TBIL, AST, and ALT in the BDL group were markedly increased, and reduced by miR-183-5p inhibition except the TBIL (Figures 2E,F). Western blotting showed that the expression of α-SMA, collagen I, and TIMP-1 in the BDL group increased, whereas miR-183-5p inhibition reduced their expression (Figures 2G,H). Moreover, immunohistochemical detection showed that miR-183-5p antagomir reduced α-SMA expression in BDL-induced liver fibrosis when compared with the control antagomir group (Figures 2I,J).

### The Inhibition of miR-183-5p Downregulated Fibrotic Markers Expression and Inhibited the Proliferation of Hepatic Stellate Cells

**FIGURE 3:**
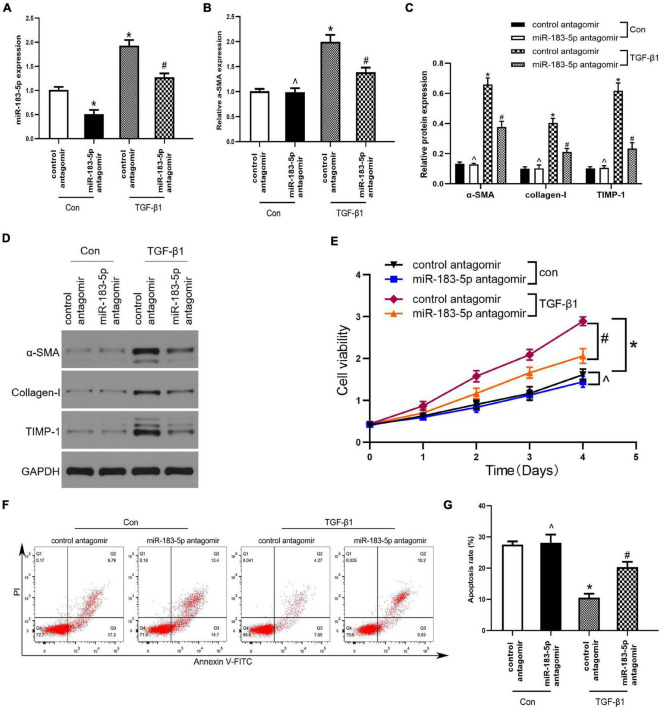
The Inhibition of miR-183-5p downregulated fibrotic markers expression and inhibited the proliferation of HSCs. **(A)** The expression of miR-183-5p in LX-2 cells was examined by RT-qPCR. **(B)** The mRNA level of a-SMA in LX-2 cells were examined by RT-qPCR. **(C,D)** The protein levels of a-SMA, Collagen-I, and TIMP-1 in LX-2 cells were examined by western blotting. **(E)** Proliferation of LX-2 cell was detected by cell viability assay. **(F)** The cell apoptosis of LX-2 cells was showed by flow cytometry. **(G)** The quantification of the apoptosis rate of the LX-2 cells. Data are expressed as the mean ± SD. *n* = 3 per group. *p < 0.05 vs. control antagomir con group, #*P* < 0.05 vs. control antagomir TGF-β1 group, ^*p* > 0.05 vs. control antagomir con group, one-way ANOVA.

Under normal physiological conditions, HSCs remain at rest. In this study, we used TGF-β1 to activate LX-2 cells, transfected miR-183-5p antagomir, and control antagomir within LX-2 cells. miR-183-5p was upregulated in the TGF-β1 group, whereas miR-183-5p was downregulated after being transfected with miR-183-5p antagomir, according to the result of RT-qPCR (Figure 3A). We detected that α-SMA was upregulated in TGF-β1-induced LX-2 cells, while miR-183-5p inhibition can reduce its expression level, according to the result of RT-qPCR (Figure 3B).

In addition, the expression of α-SMA, collagen I, and TIMP-1 increased after being treated with TGF-β1, whereas inhibition of miR-183-5p reduced their expression, when compared with the control antagomir TGF-β1group (Figures 3C,D). Next, we evaluated whether miR-183-5p influenced the proliferation and apoptosis of HSCs. Cell viability assays revealed that the TGF-β1 treated group had an increased proliferative ability when compared with control antagomir TGF-β1 group, and miR-183-5p inhibition reduced proliferative ability (Figure 3E). According to the result of Flow cytometry analysis, in the TGF-β1 group miR-183-5p inhibition induced LX-2 cells apoptosis (Figures 3F,G). Our finding indicated that miR-183-5p inhibition can downregulate expression of fibrotic markers and proliferation of HSCs.

### Fork Head Box Protein O1 Is a Potential Target of miR-183-5p

**FIGURE 4:**
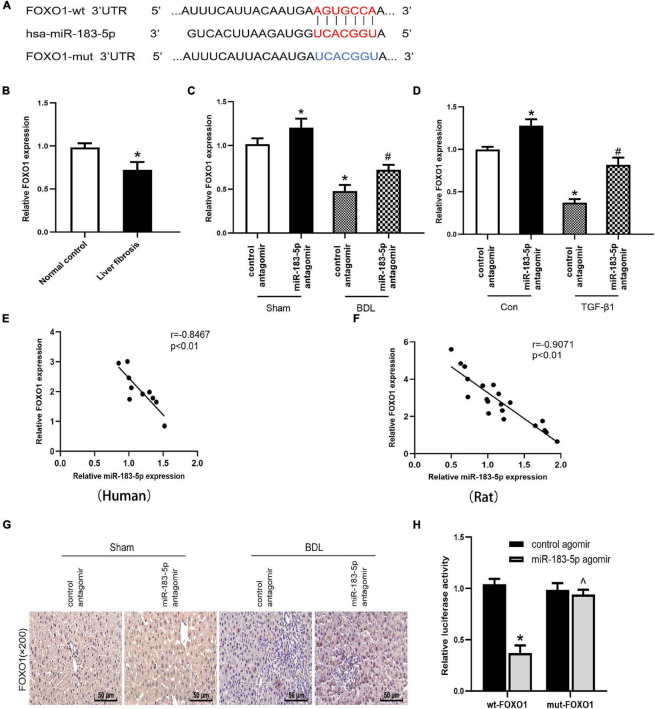
FOXO1 is a potential target gene for miR-183-5p. **(A)** Database predicted the existence of binding sites between FOXO1 3’UTR and miR-183-5p. **(B)** The mRNA levels of FOXO1 were examined in human liver tissues by RT-qPCR. **(C)** The FOXO1 expression in rat liver tissues was examined by RT-qPCR. **(D)** The FOXO1 expression in LX-2 cells was examined by RT-qPCR. **(E)** The expression of miR-183-5p in human was inversely correlated with the mRNA expression of FXOX1 by Pearson’ s correlation analysis. **(F)** The expression of miR-183-5pin rats was inversely correlated with the mRNA expression of FXOX1 by the Pearson’s correlation analysis. **(G)** Immunohistochemical staining of FOXO1 in rat liver tissues. 200×, Scale bar = 50 μm. **(H)** Verification of binding relationship between miR-183-5p and FOXO1 by the Dual-reporter luciferase assay. Data are expressed as the mean ± SD. *n* = 5 per group in liver tissues and *n* = 3 per group in LX-2 cells. *P < 0.05 vs. normal control group, control antagomir Sham group, control antagomir con group and wt –FOXO1 group, #*P* < 0.05 vs. control antagomir BDL group, control antagomir TGF-β1 group, ^*P* > 0.05 vs. mut –FOXO1 group, one-way ANOVA.

Results of bioinformatics analysis (TargetScan, miRDB, and miRbase) revealed that FOXO1 3′ UTR may have a complementary transcriptomic sequence conserved for miR-183-5p (Figure 4A). The other potential targets of miR-183-5p are shown in Table 3. A previous study has shown that low-expressed FOXO1 could promote the activation of HSC and stimulate the development of hepatic schistosome fibrosis (Huang et al., 2018). First, we preliminarily analyzed the association between miR-183-5p and FOXO1 by detecting FOXO1 expression in cholestatic liver fibrosis and normal fibrosis. RT-qPCR analysis revealed that FOXO1 expression was reduced in hepatic fibrosis (Figure 4B). Next, FOXO1 levels were quantified *in vivo* and *in vitro*. FOXO1 mRNA expression decreased in both the liver tissue of BDL-induced rats and in activated LX-2 cells, according to the RT-qPCR analysis result. And we also found that the application of miR-183-5p antagomir could increase the expression of FOXO1 (Figures 4C,D). Moreover, according to the result of the Pearson’s correlation analysis, FOXO1 expression was inversely proportional to that of miR-183-5p both in human tissues and BDL tissues (Figures 4E,F). FOXO1 protein expression was evaluated by immunohistochemistry in rat liver tissues. We found that the expression level ofFOXO1protein in tissue samples of BDL group was significantly reduced compared with the normal group, and delivered miR-183-5p antagomir *in vivo*, which could increase the expression of FOXO1 in liver tissues (Figure 4G). The dual-luciferase reporter gene assay was used to verify the direct interaction between miR-183-5p and FOXO1. Also wt-FOXO1 and mut-FOXO1 sequences of the putative miR-183-5p-binding sites on FOXO1 3′UTR were cloned into the luciferase vector. We found that miR-183-5p agomir reduced the luciferase activity of wt-FOXO1, but the luciferase activity of mut-FOXO1 was unaffected (Figure 4H). Therefore, we speculated that miR-183-5p may regulate FOXO1 expression in cholestatic liver fibrosis.

**TABLE 3:** The potential targets of miR-183-5p.

miRNA	Target gene
miR-183-5p	PSEN2, PTS, TMSB4X, HTR2A, SPRY2, MTA1, SRSF2, CRK, TPM1, PAM, IRS1, LRP6, NRG1, ATP2C1, RPS6KA3, TCF7L2, ZFPM2, PDCD4, FOXP1, PKD2, MAP3K4, CELF1, PPP2R2A, CX3CL1, RORA, PDE4D, EZR, SPRY3, ITGB1, TAOK1, SP2, SESN1, ATF2, SACS, ERN1, TET1, NR3C1, DAP, RALA, OCLN, GPAM, DPP8, ZEB1, THSD7A, CBL, TUB, DTNA, INSIG1, BRD4, EGR1, SAMD4A, DAGLA, HN1, HLF, CPEB1, SLC6A6, PDE3A, PAX5, CELSR3, NR4A2, TRIM2, MARK2, SEMA6D, PIGA, HBEGF, MED1, PRKCA, SKIL, SCARF1, FN1, ABCA1, NRXN3, MECOM, MTDH, RSF1, SIN3A, RAP2C, and RREB1

### Fork Head Box Protein O1 Negatively Mediated Hepatic Stellate Cells Activation by Suppressing the TGF-β Pathway

**FIGURE 5:**
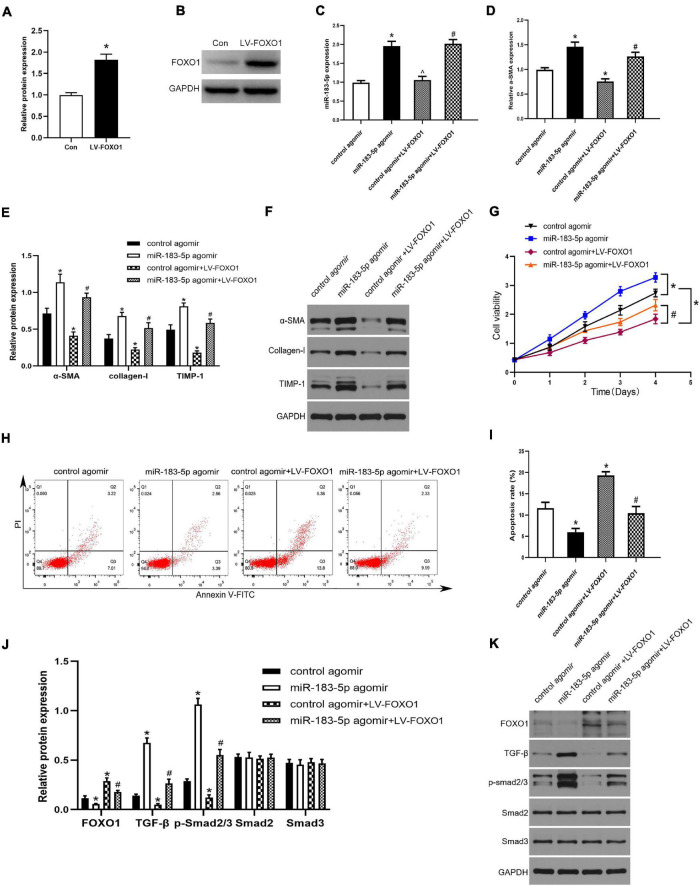
FOXO1 negatively mediated HSC activation by suppressing the TGF-β pathway. **(A,B)** The protein level of FOXO1 in LX-2 cells after transfected with lentivirus were examined by western blotting. **(C)** The expression of miR-183-5p in LX-2 cells was examined by the RT-qPCR. **(D)** The mRNA level of a-SMA in LX-2 cells were examined by RT-qPCR. **(E,F)** The protein levels of a-SMA, Collagen-I, and TIMP-1 in LX-2 cells were examined by western blotting. **(G)** Proliferation of LX-2 cells was detected by cell viability assay. **(H)** The cell apoptosis of LX-2 cells was showed by flow cytometry. **(I)** The quantification of the apoptosis rate of the LX-2 cells. **(J,K)** The protein levels of FOXO1, TGF-β, p-smad2/3, Smad2, and Smad3 in LX-2 cells were examined by western blotting. Data are expressed as the mean ± SD. *n* = 3 per group. *P < 0.05 vs. Con group, control agomir group, #*P* < 0.05 vs. control agomir LV-FOXO1 group, *^P* > 0.05 vs. control agomir group, one-way ANOVA.

To further clarify the specific mechanism of miR-183-5p and FOXO1 in liver fibrosis, we used a lentivirus to overexpress FOXO1 in activated LX-2 cells, which transfected with the control agomir and miR-183-5p agomir. First, we detected the upregulation of FOXO1 expression in cells after being transfected with lentivirus by western blotting, proving that the lentivirus was successfully transfected (Figures 5A,B). Next, we found that miR-183-5p was upregulated after treatment with the miR-183-5p agomir, but overexpression of FOXO1 does not change its expression level (Figure 5C). Next, we detected that the level of α-SMA was increased after being transfected with miR-183-5p agomir. This suggested that HSCs are further activated, while overexpression of FOXO1 can reduce its expression level (Figure 5D). The overexpression of miR-183-5p increased the expression of α-SMA, collagen I, and TIMP-1, but the overexpression of FOXO1 led to their downregulation (Figures 5E,F). In addition, the overexpression of miR-183-5p promoted LX-2 cell proliferation and inhibited apoptosis, whereas the overexpression of FOXO1 had the opposite effect (Figures 5G–I). Moreover, western blotting revealed that overexpression of miR-183-5p inhibited FOXO1 expression and promoted the expressions of TGF-β and p-Smad2/3. FOXO1 overexpression resulted in the upregulation of FOXO1 and prevented the activation of TGF-β signaling pathway (Figures 5J,K). Thus, the miR-183-5p/FOXO1/TGF-β/Smad2/3 axis plays a key role in modulating activation and proliferation of HSCs.

## Discussion

Liver fibrosis is a manifestation of multiple chronic hepatic conditions. In chronic liver injury or inflammation, the liver cannot regenerate, and turns fibrotic (Seki and Brenner, 2015). Progressive and persistent hepatic fibrosis can gradually develop into liver cirrhosis and even cancer (Ma et al., 2017). Activated HSCs are a marker of fibrosis (Huang et al., 2018). α-SMA is a biomarker of activated HSCs.

Bile duct obstruction causes cholestasis and increases bilirubin and bile acid levels in the serum. It causes the inflammation of intrahepatic cells, promotes the activation of HSCs, and accelerates the transition to fibroblasts. HSCs constitute 82%–96% of sourcing fibroblasts within cholestatic-models-induced liver fibrosis models (Mederacke et al., 2013), which can cause the accumulation of inflammatory cells, ECM, and fibers in liver tissues eventually leading to liver fibrosis. Identifying potential molecular and pathway targets to revert or halt the advance of hepatic fibrosis into cirrhosis and HCC is an emerging field.

Recently, with the development of bioinformatics and epigenetics, the role of miRNAs has been gradually discovered in the liver disease. Mounting evidence suggests that miRNA-29a inhibits CD36 and improves steatohepatitis and liver fibrosis induced by a high-fat diet in mice (Lin et al., 2019). miRNA-322/424 exacerbates hepatic fibrosis by influencing the CUL2/HIF-1α axis to regulate angiogenesis (Wang et al., 2021). Rno-miR-183-96-182 is upregulated in diethylaminoethylamine-induced hepatic fibrosis, which could play a key role in in the state of fibrosis and its progression (Chandel et al., 2018). However, this role and the mechanistic function still need to be clarified. In this study, we performed a high-throughput sequencing using cholestatic liver fibrosis tissues, matched normal liver tissues, and identified 44 differentially expressed miRNAs (18 upregulated and 26 downregulated). miR-183-5p was found to be upregulated in human cholestatic fibrosis. The results of RT-qPCR analysis indicated that miR-183-5p was upregulated in BDL-induced liver fibrosis and activated LX-2 cells; this result was consistent with the previous sequencing results. Furthermore, we found that inhibiting miR-183-5p alleviated liver fibrosis and downregulated the expression of related fibrotic biomarkers. Meanwhile, the inhibition of miR-183-5pinhibited LX-2 cell proliferation and promoted apoptosis. Our results indicate that miR-183-5p plays a key role in the occurrence and development of liver fibrosis and its upregulation may be related to the activation of HSCs.

However, in the research, we found that inhibiting miR-183-5p cannot be recovered to basal levels in both animal (2F and 2G) and culture experiments (3C). The reasons may be as follows: First, liver fibrosis is a pathological condition originating from liver damage, so removing the cause is the most effective way to treat liver fibrosis. For example, removal of obstructive agent or surgery is the main option in obstructive cholestasis (Kumar and Mahato, 2015). Next, it is known that miRNAs can alter gene expression at the posttranscriptional level in many developmental and physiological processes. Their expression profiles are different under normal and disease states, and can also be used for diagnostic, treatment and prognostic purposes (Szabo and Bala, 2013). Generally, a single miRNA can be fine-tuned to regulate many different target genes and contributes to biological processes. However, there are also a variety of miRNAs jointly involved in regulating the biological process of liver fibrosis. Previous studies found that miR-150-5p can reduce the activation of HSCs by inhibiting MYB, SP1, COL4A4, HOTTIP, and RAC1 (Chen et al., 2020). Dongiovanni et al. (2018) summarized that upregulation of miR-34a, miR-33a/b, miR-21, and miR-221/222, and downregulation of miR-15/16, miR-122, and miR-192 could activate HSCs and promote hepatocyte apoptosis, which may affect the progression of liver fibrosis (Dongiovanni et al., 2018). At the same time, our high-throughput sequencing found a total of 44 differentially expressed miRNAs. Therefore, the “one miRNA to one target” research model may not fully explain the biological process of the disease. There may be other miRNAs involved in the regulation of liver fibrosis in this study, so follow-up experiments are still needed to further verify the specific mechanisms.

We then used miRbase, TargetScan, and miRDB for bioinformatics analysis and predicted that FOXO1 may be the downstream for the miR-183-5p-binding target gene. Proteomic/transcriptomic FOXO1 expression levels in human fibrotic liver specimens, BDL rat liver, and activated LX-2 cultures were all downregulated. Furthermore, luciferase experiments verified that miR-183-5p modulates FOXO1expression by directly binding to the 3′UTR of its mRNA. FOXO1 is a member of the forkhead transcription factor family, which plays vital roles in oxidative stress suppression, cell cycle arrest, proliferation regulation, apoptosis induction, protein synthesis, and other biological activities. The transcriptional activity of FOXO1 is negatively regulated by the phosphatidylinositol-3-hydroxy kinase/protein kinase B (PI3K/Akt) pathway. Studies have confirmed that FOXO1 plays a role in many diseases, such as cardiovascular diseases, diabetes, cancer, aging, and stem cell activation (Xin et al., 2018). For example, miR-590-5p exacerbates liver cancer expansion and chemoresistance by directly acting on FOXO1 (Jia et al., 2019). Exosomes derived from colorectal cancer cells overexpressing miR-183-5p aggravate colorectal cancer by regulating FOXO1 (Shang et al., 2020). FOXO1 also modulates activation of HSC sand liver fibrosis (Li et al., 2015). For example, miR-182 inhibits FOXO1 expression through the PI3K/AKT signaling pathway, thus aggravating schistosomiasis-mediated liver fibrosis (Huang et al., 2018). FOXO1 can reduce tubulointerstitial fibrosis and tubular apoptosis in diabetic nephropathy by targeting STAT1 signaling (Huang F. et al., 2019). Consequently, we inferred that FOXO1 is involved in liver fibrosis induced by miR-183-5p. To further investigate this question, we examined FOXO1 expression in HSCs transfected with the miR-183-5p agomir. We found that miR-183-5p overexpression reduced FOXO1 levels. However, overexpression of FOXO1 reduced the fibrotic biomarker levels in LX-2 cells, inhibited proliferation, and promoted apoptosis of LX-2 cells. Moreover, overexpression of FOXO1 inhibited the TGF-β pathway to disrupt the activation of HSC, and reduced TGF-β, p-Smad2, and p-Smad3 levels in HSCs.

Transforming growth factor-β is a cellular factor, which is related to a variety of bioactivities, such as cell proliferation, apoptosis, migration and, differentiation (Borges et al., 2013). Moreover, the TGF-β pathway plays a key role in activation of HSCs (Perumal et al., 2017). TGF-β exerts its profibrosis effect through cascade stimulation of Smad proteins in downstream cells (Sulaiman et al., 2016). The TGF-β pathway can interact with FOXO1 pathway to regulate fibrosis (Hameedaldeen et al., 2014). Experiments have shown that TGF-β upregulates FOXO1 in cardiac fibroblasts (Vivar et al., 2016). In summary, these suggest that abnormal expression of FOXO1 may be associated with miR-183-5p and TGF-β signaling pathway, influencing the occurrence and development of hepatic fibrosis.

The occurrence and development of liver fibrosis is the result of multiple factors. In subsequent experiments, in order to further research the mechanism of FOXO1 for cholestatic liver fibrosis, we need to select one or more other putative miR-183-5p targets as controls. In addition, another limitation of this study was the lack of a large number of liver fibrosis cases to further study the expression of miR-183-5p and FOXO1 and their correlation with pathological features. Moreover, the effect of miR-183-5p on liver fibrosis caused by other etiologies and injuries has not yet been carried out, so we still need follow-up experiments for further verification.

**FIGURE 6:**
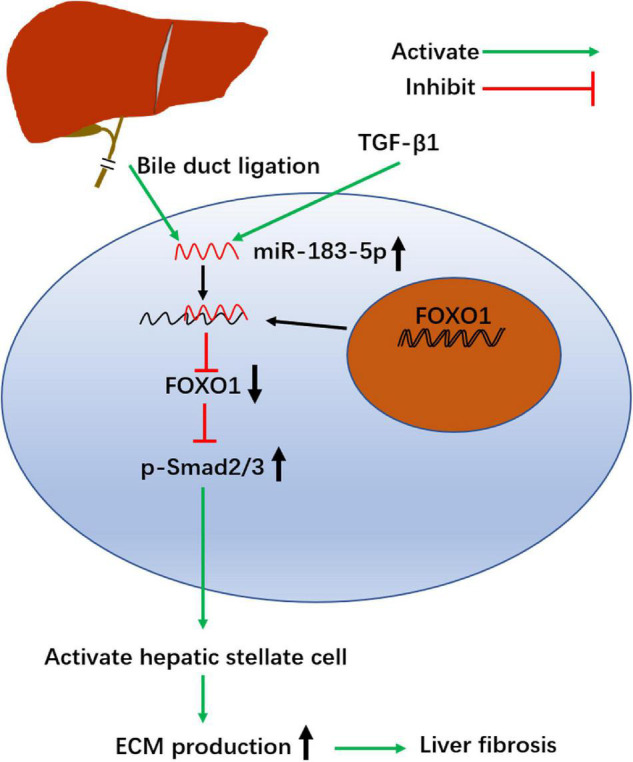
Schematic summary of the profibrosis effect of miR-183-5p in hepatic stellate cells. BDL and TGF-β1 up regulates the level of miR-183-5p in HSCs. miR-183-5p down regulates FOXO1 expression and increases p-Smad2/3 expression, thereby promoting HSC activation and stimulating extracellular matrix (ECM) production, which ultimately contributes to the occurrence and progression of liver fibrosis.

In summary, our study shows that miR-183-5p is upregulated, whereas FOXO1 is poorly expressed, in activated HSCs and cholestatic liver fibrosis models. High expression of miR-183-5p and low expression of FOXO1 promotes proliferation of HSCs, inhibits apoptosis, stimulates fibrosis, and involves the TGF-β signaling pathway (Figure 6). In summary, our data reveal a complex relationship between FOXO1, miR-183-5p, and the TGF-β signaling pathway, which might provide clues for the development of more effective strategies to treat cholestatic liver fibrosis.

## Data Availability Statement

The original contributions presented in the study are included in the article/Supplementary Material, further inquiries can be directed to the corresponding author.

## Ethics Statement

The studies involving human participants were reviewed and approved by Qingdao Municipal Hospital Medical Ethics Committee. The patients/participants provided their written informed consent to participate in this study. The animal study was reviewed and approved by the Institutional Animal Care and Use Committee of Qingdao Municipal Hospital.

## Author Contributions

YW and ZG designed the research, analyzed the data, and drafted the manuscript. YW, ZG, BC, JY, and XB performed the operation and experiments on patients. CX and FJ helped with data acquisition and discussion. WD collected the specimen. YW and ZG analyzed the data and prepared the figures. All authors contributed to the article and approved the submitted version.

## Conflict of Interest

The authors declare that the research was conducted in the absence of any commercial or financial relationships that could be construed as a potential conflict of interest.

## Publisher’s Note

All claims expressed in this article are solely those of the authors and do not necessarily represent those of their affiliated organizations, or those of the publisher, the editors and the reviewers. Any product that may be evaluated in this article, or claim that may be made by its manufacturer, is not guaranteed or endorsed by the publisher.
