# Supplementary figures and images for: Effect of miR-183-5p on Cholestatic Liver Fibrosis by Regulating Fork Head Box Protein O1 Expression
Source: Front Physiol. 2021 Nov 18;12:737313. doi: 10.3389/fphys.2021.737313 (PMC8639207; doi:10.3389/fphys.2021.737313)

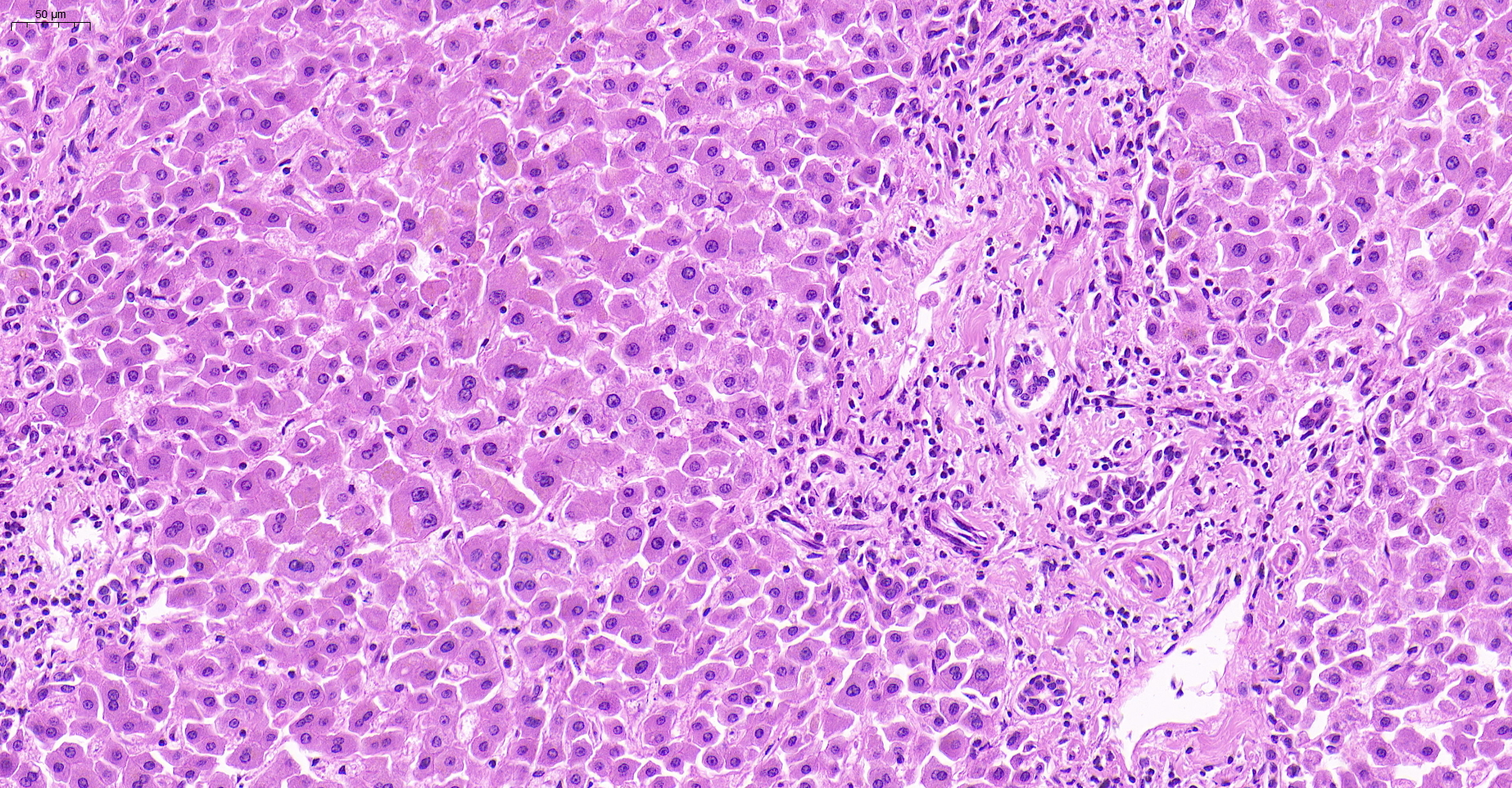

Supplement: Supplementary file 1 [file Data_Sheet_1.ZIP › Figure1 original data/microscopy images/fibrosis HE.jpg]

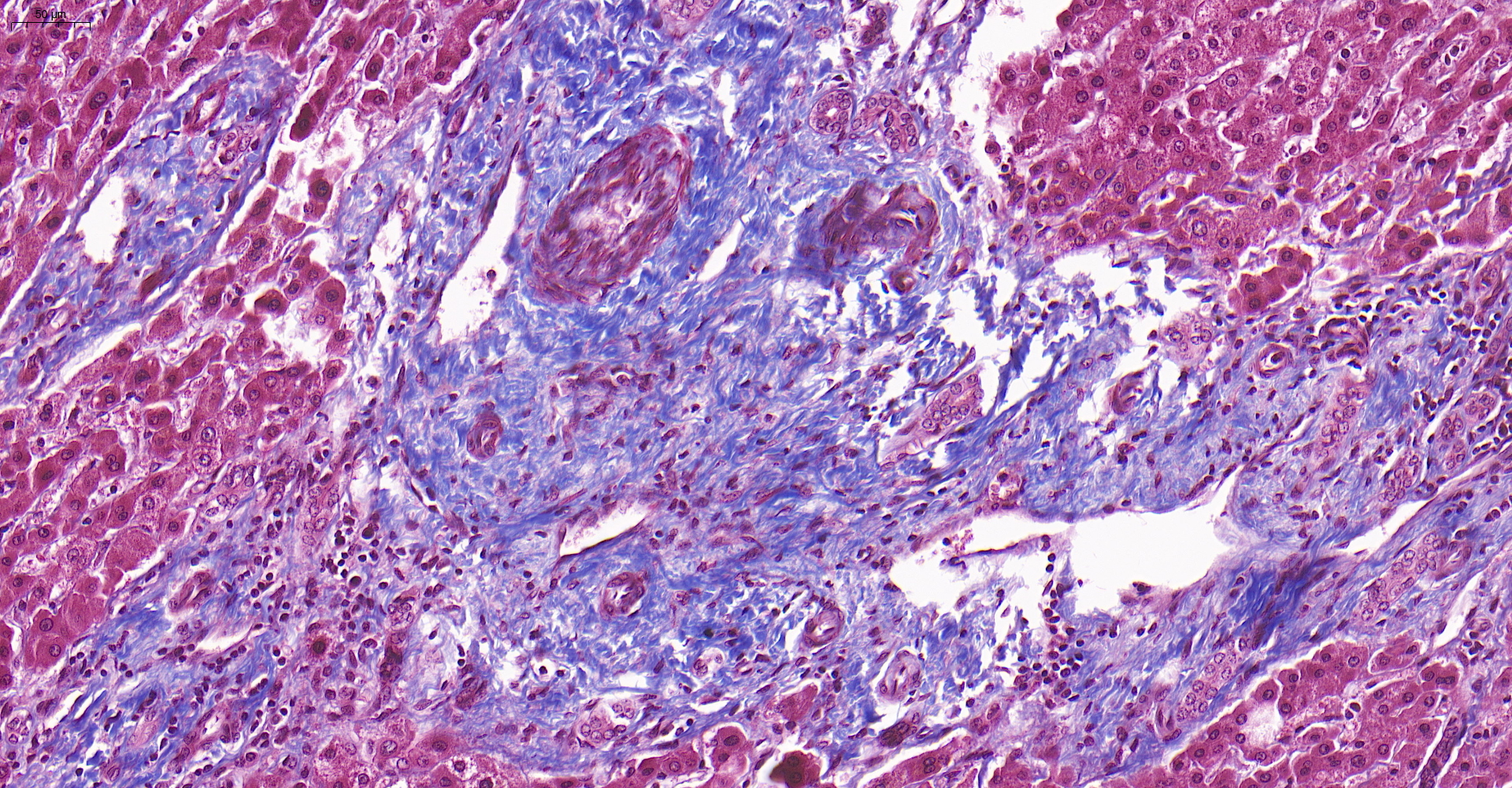

Supplement: Supplementary file 1 [file Data_Sheet_1.ZIP › Figure1 original data/microscopy images/fibrosis Masson.jpg]

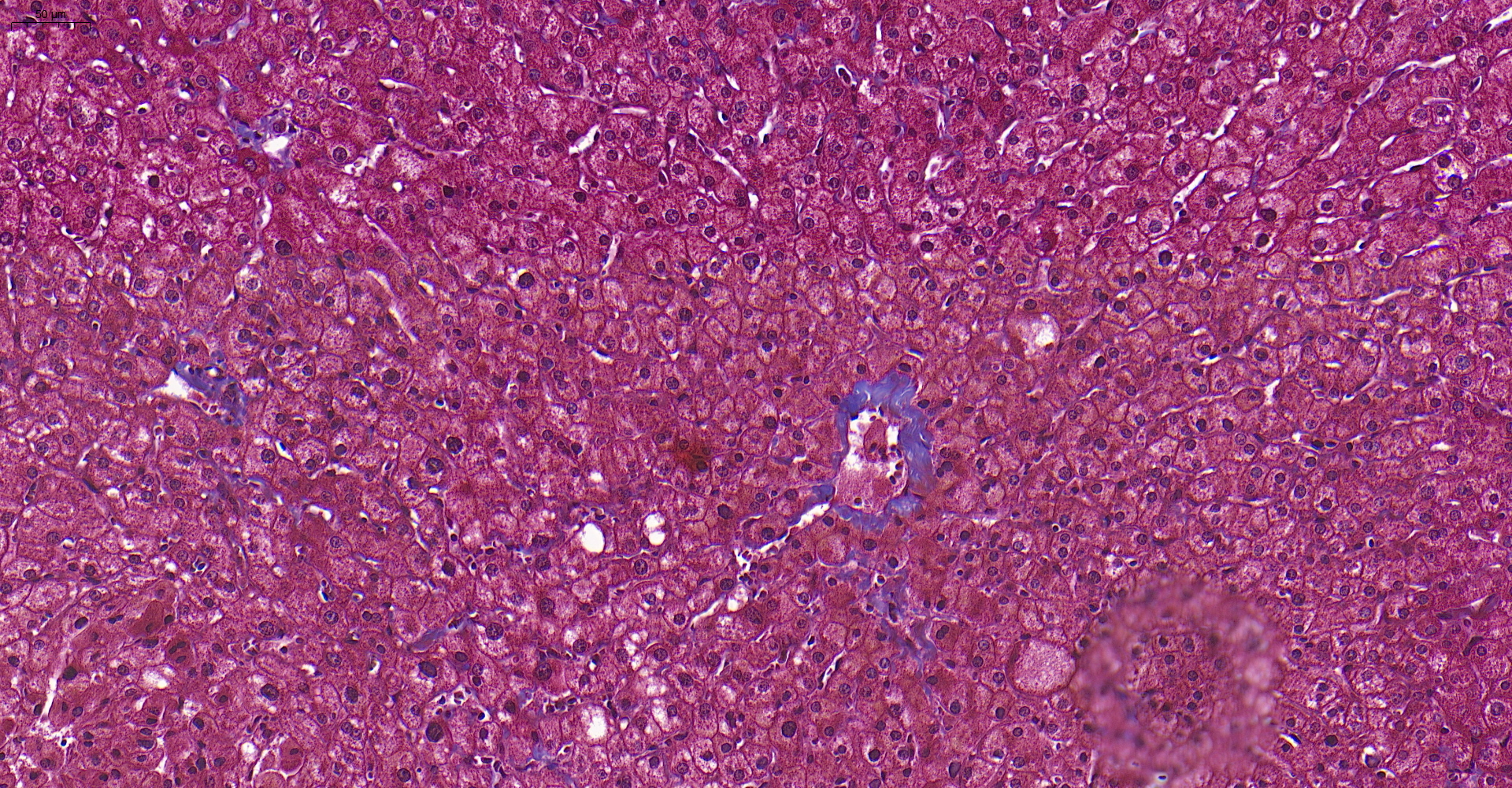

Supplement: Supplementary file 1 [file Data_Sheet_1.ZIP › Figure1 original data/microscopy images/normal Masson.jpg]

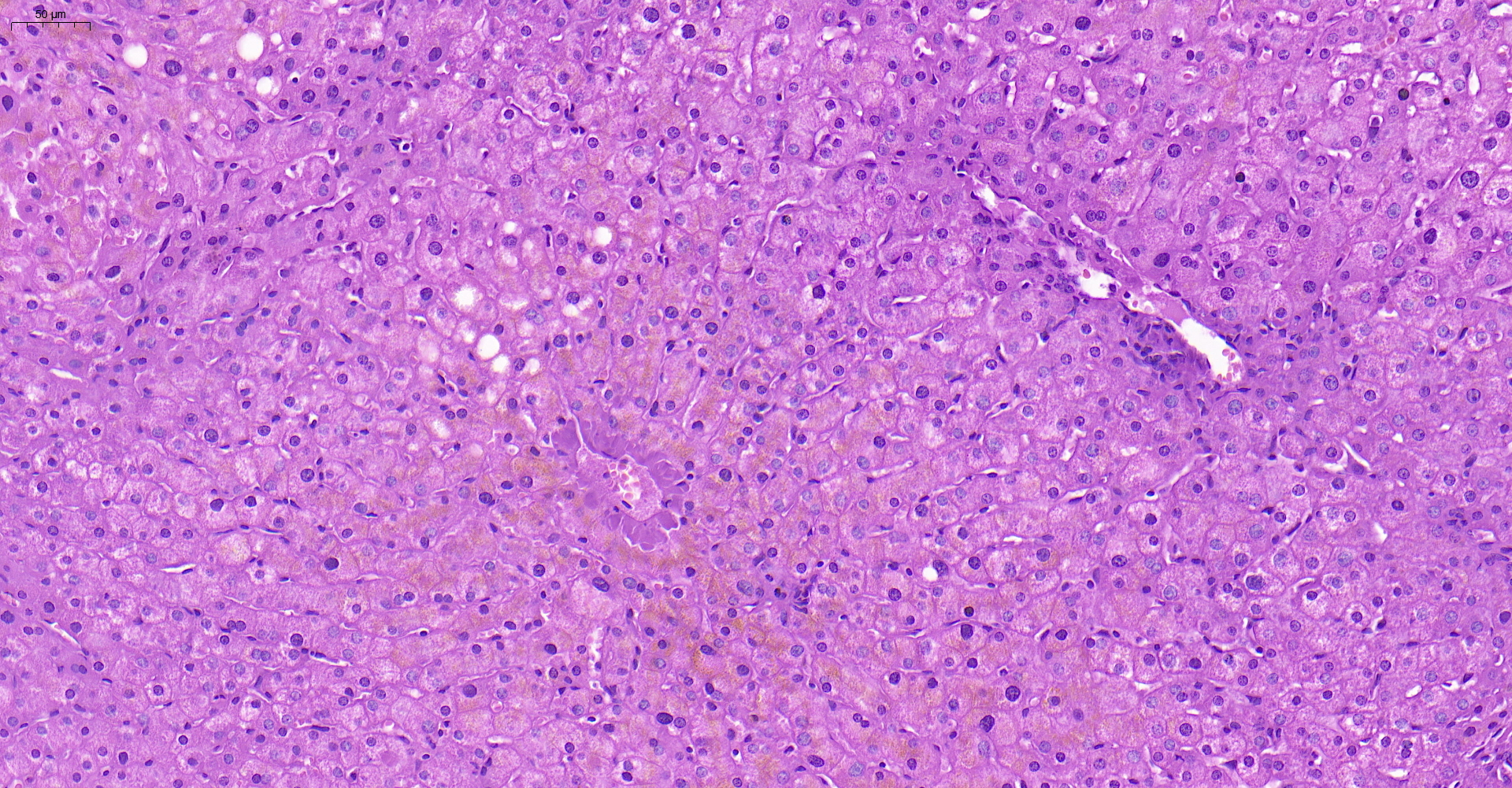

Supplement: Supplementary file 1 [file Data_Sheet_1.ZIP › Figure1 original data/microscopy images/normalHE.jpg]

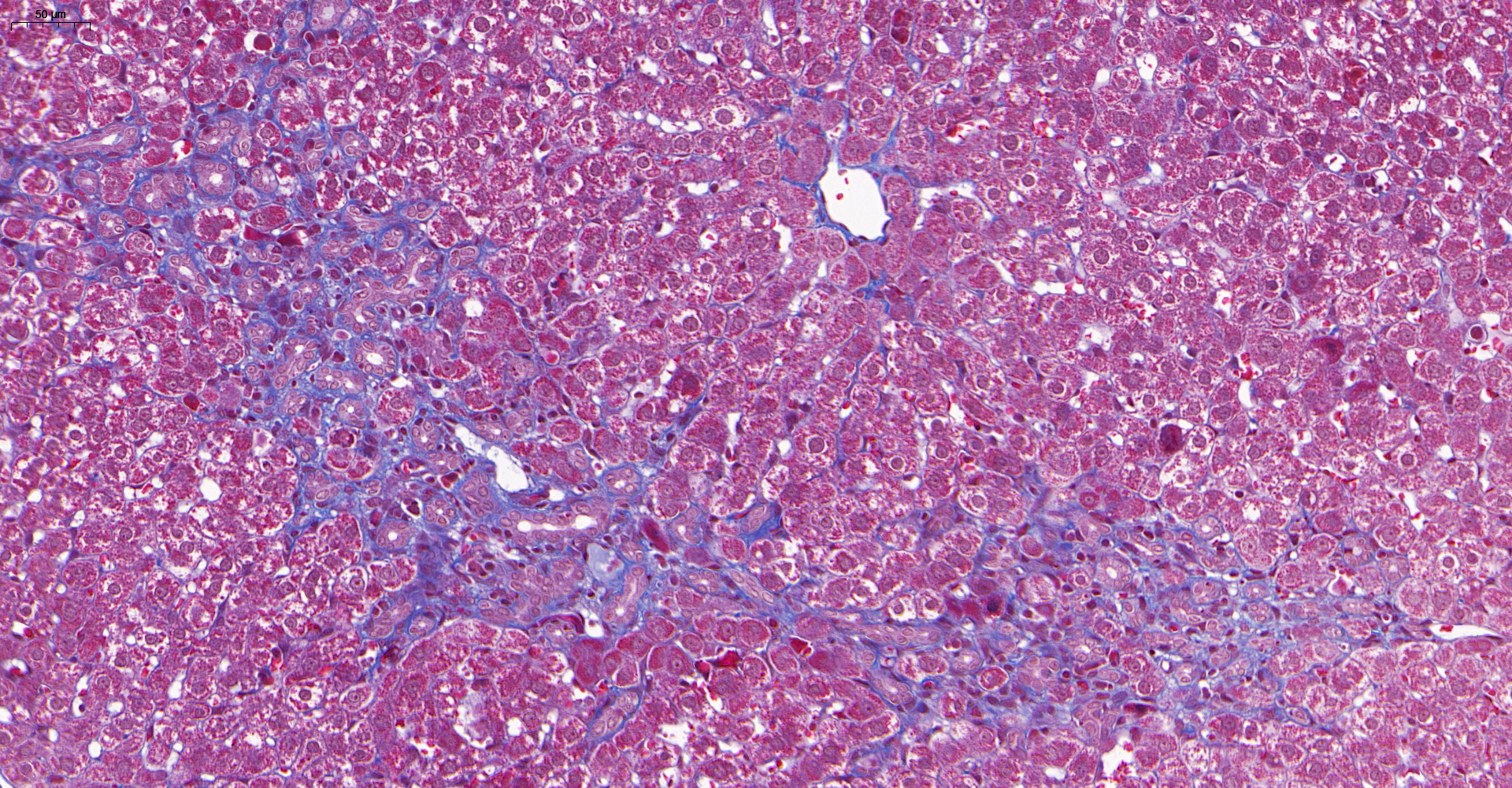

Supplement: Supplementary file 2 [file Data_Sheet_2.ZIP › Masson/control antagomir BDL group.tif]

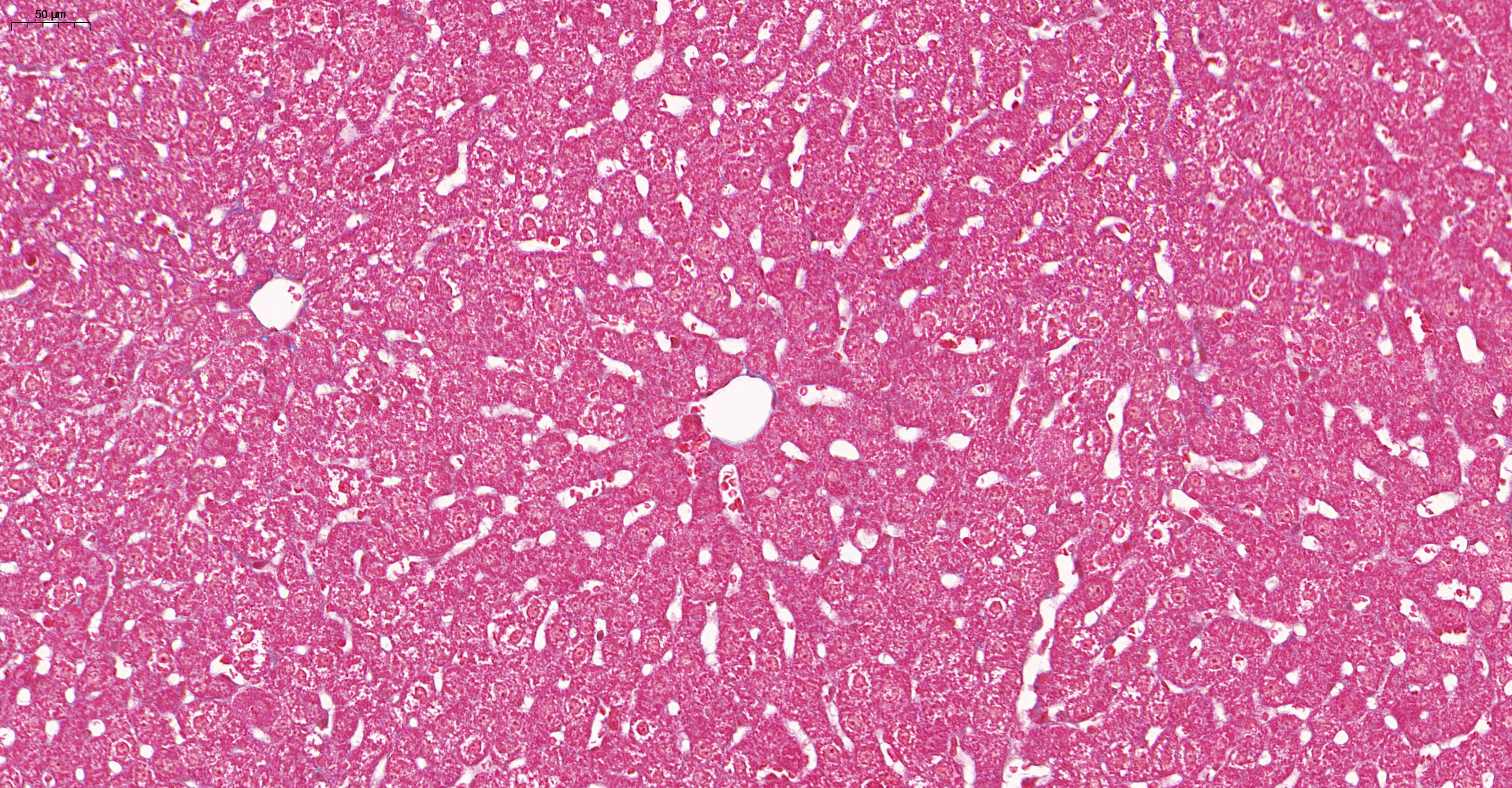

Supplement: Supplementary file 2 [file Data_Sheet_2.ZIP › Masson/control antagomir Sham group.tif]

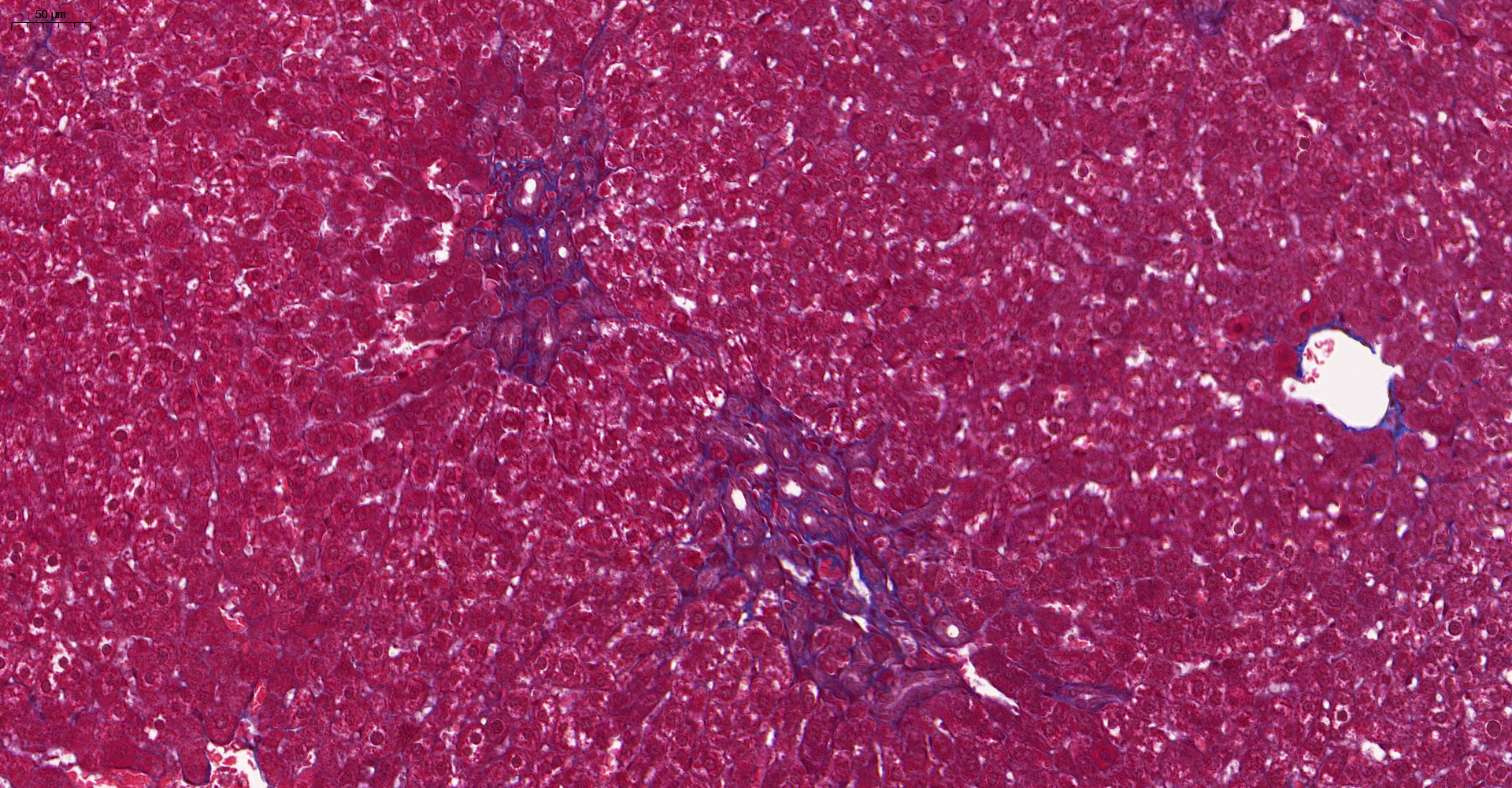

Supplement: Supplementary file 2 [file Data_Sheet_2.ZIP › Masson/miR-183-5p antagomir BDL group.tif]

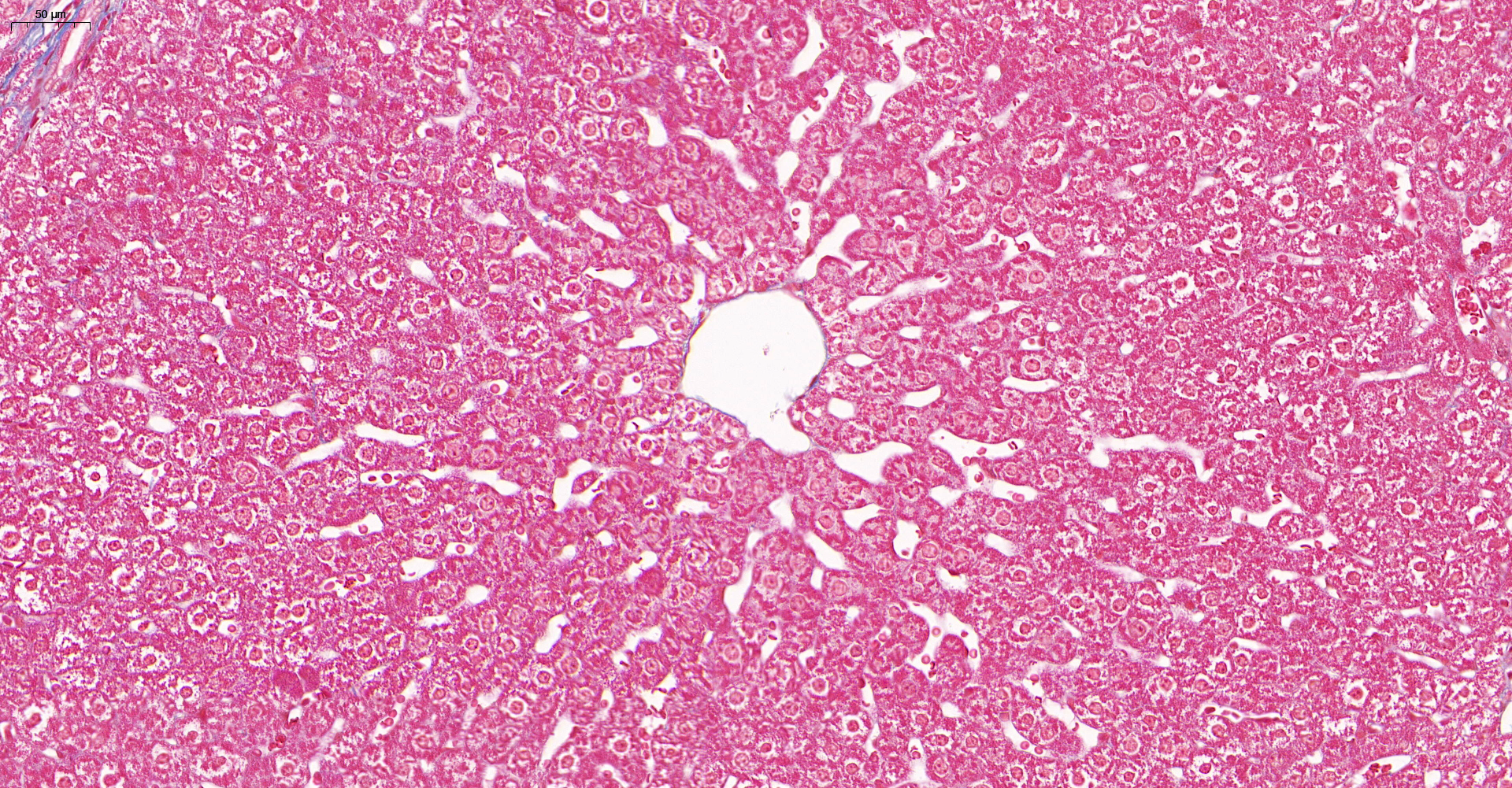

Supplement: Supplementary file 2 [file Data_Sheet_2.ZIP › Masson/miR-183-5p antagomir Sham group.tif]

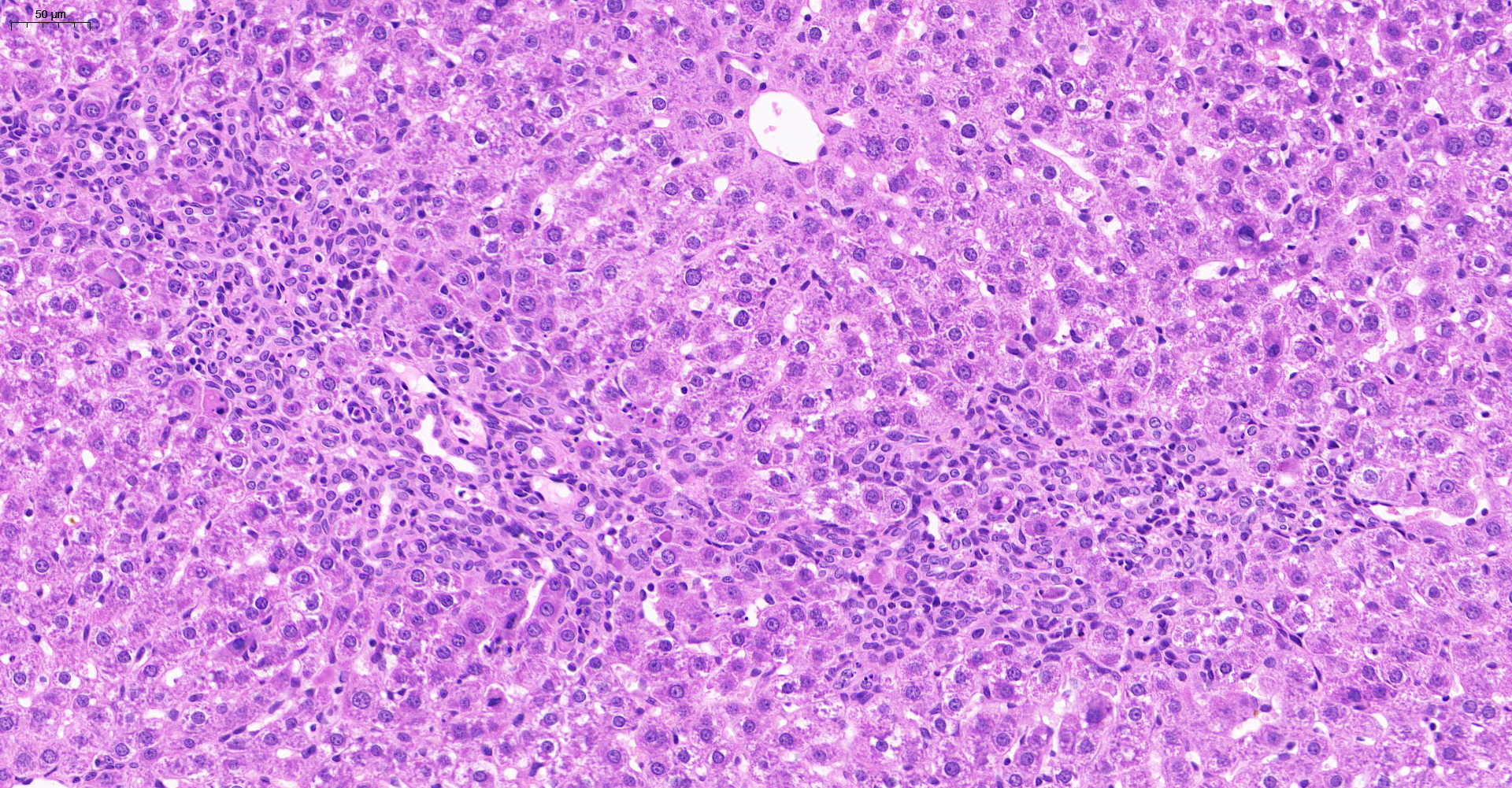

Supplement: Supplementary file 3 [file Data_Sheet_3.ZIP › HE/control antagomir BDL group.tif]

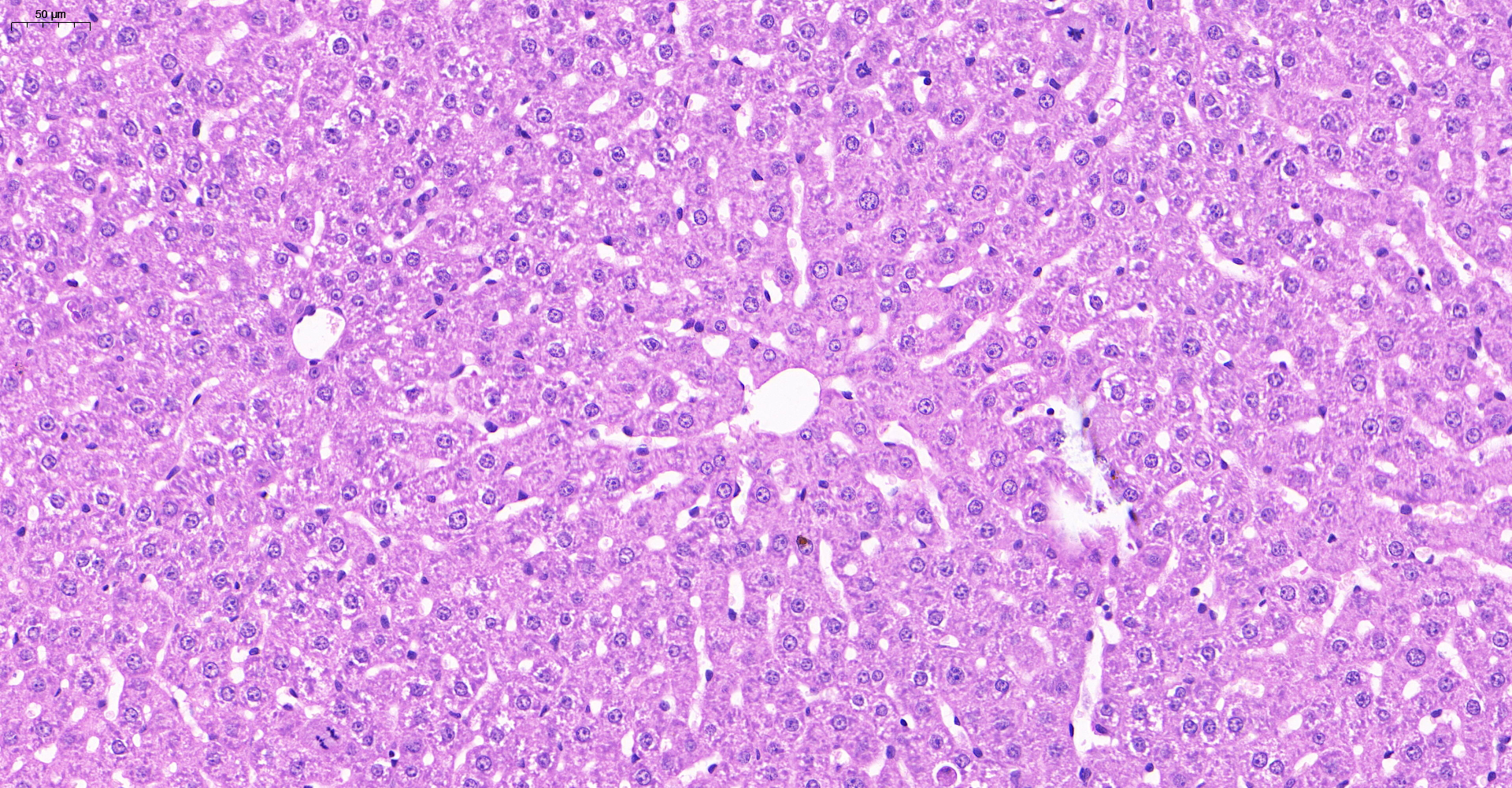

Supplement: Supplementary file 3 [file Data_Sheet_3.ZIP › HE/control antagomir Sham group.tif]

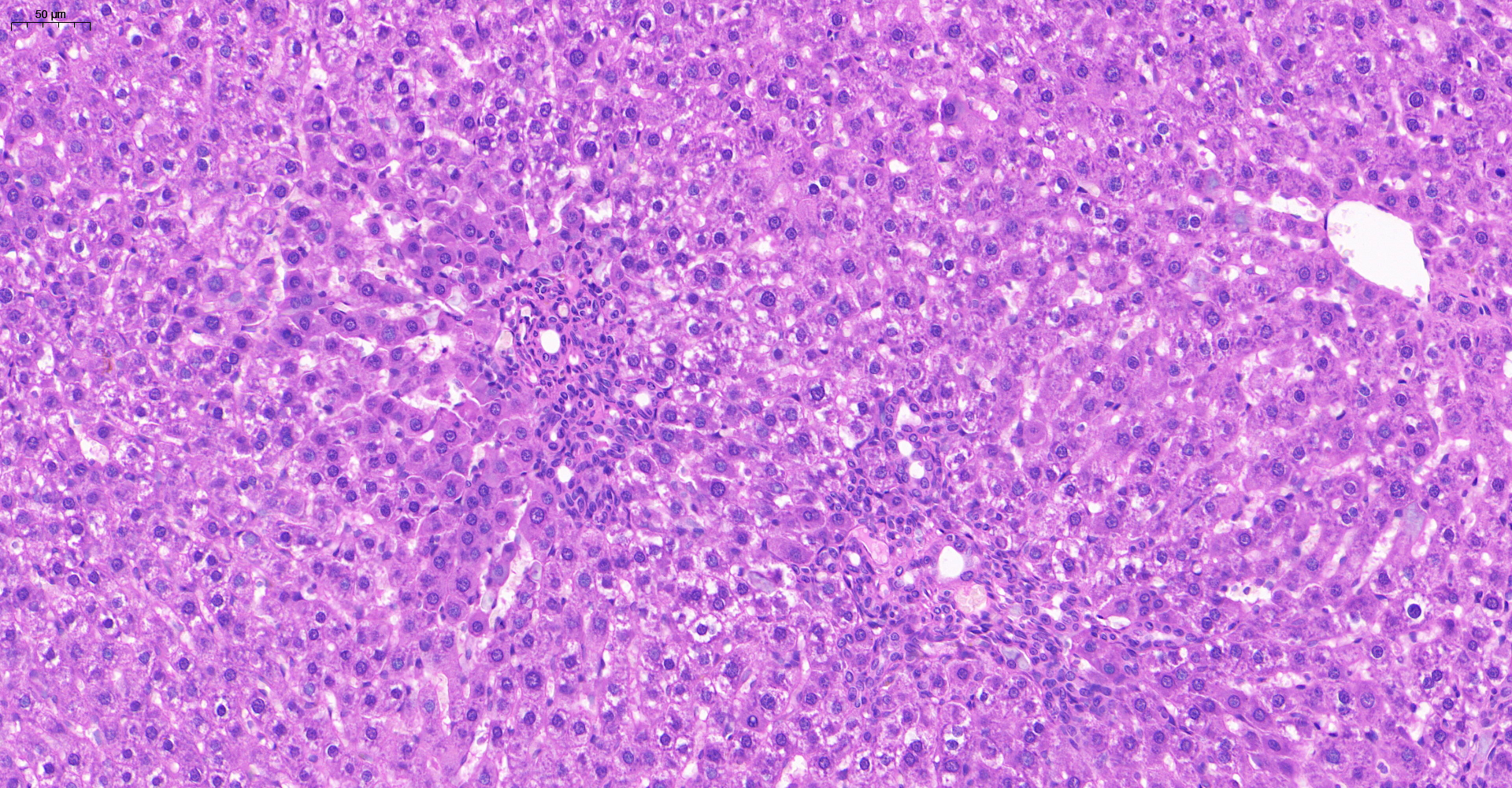

Supplement: Supplementary file 3 [file Data_Sheet_3.ZIP › HE/miR-183-5p antagomir BDL group.tif]

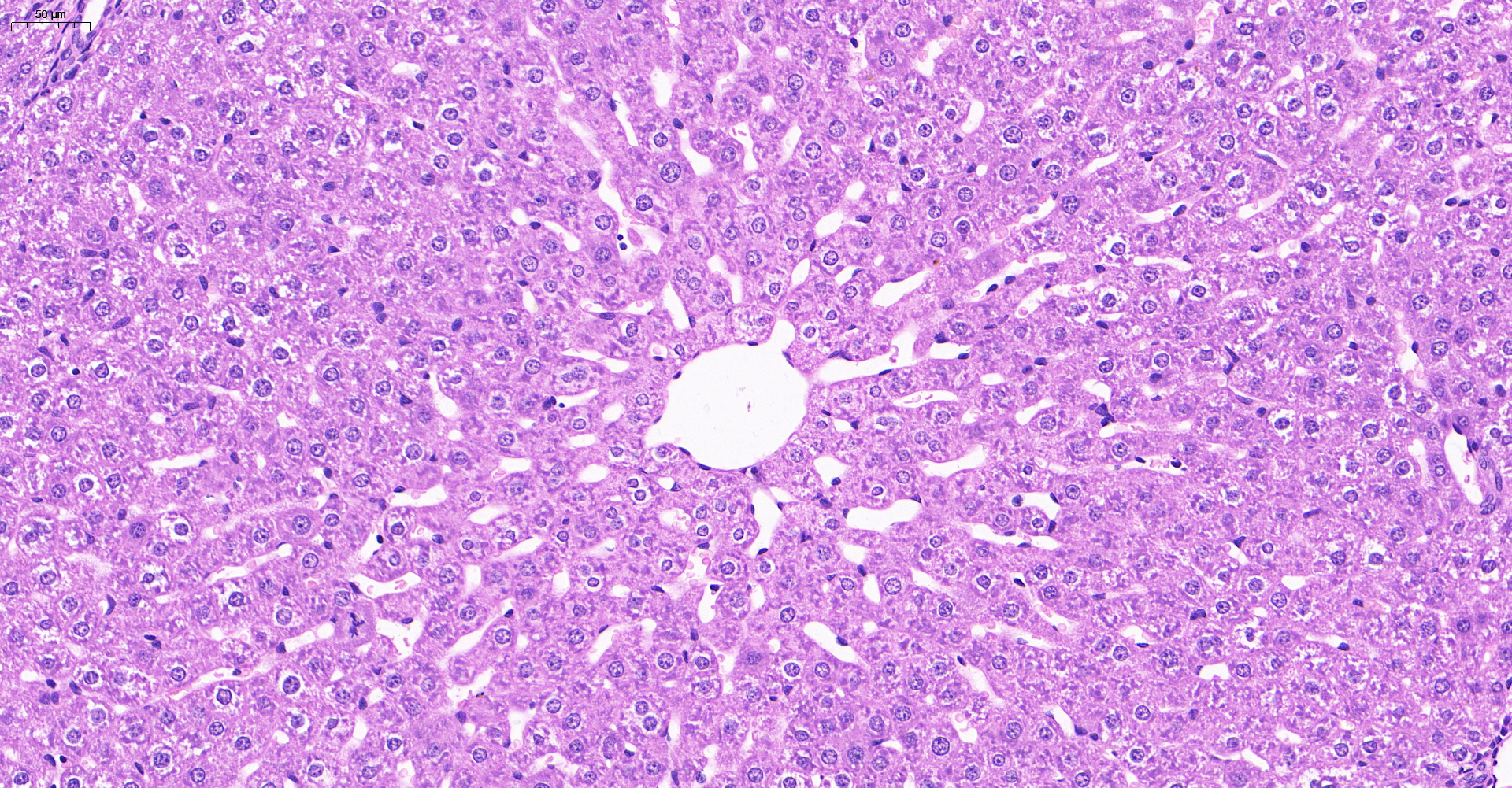

Supplement: Supplementary file 3 [file Data_Sheet_3.ZIP › HE/miR-183-5p antagomir Sham group.tif]

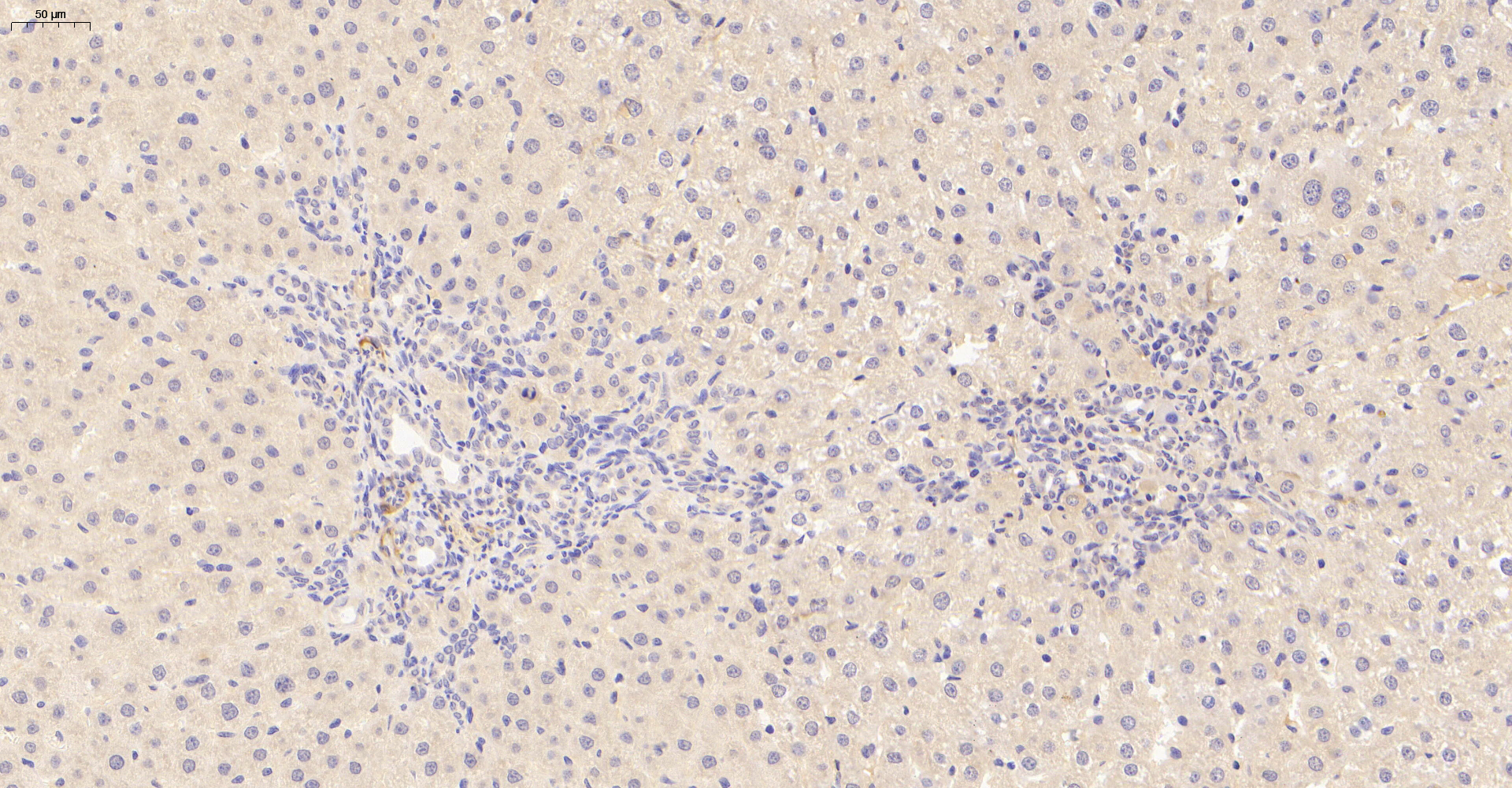

Supplement: Supplementary file 4 [file Data_Sheet_4.ZIP › IHC/control antagomir BDL group.tif]

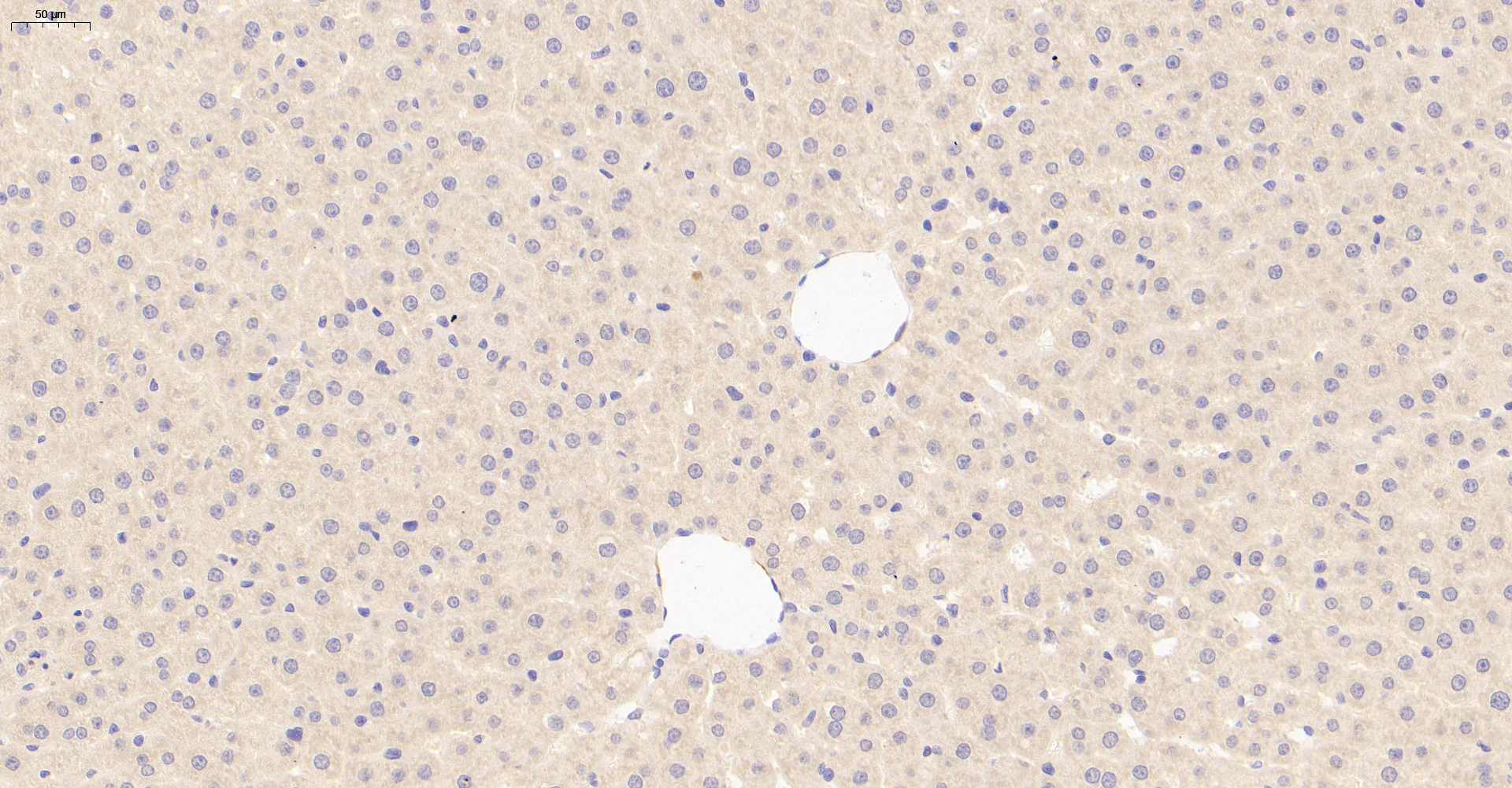

Supplement: Supplementary file 4 [file Data_Sheet_4.ZIP › IHC/control antagomir Sham group.tif]

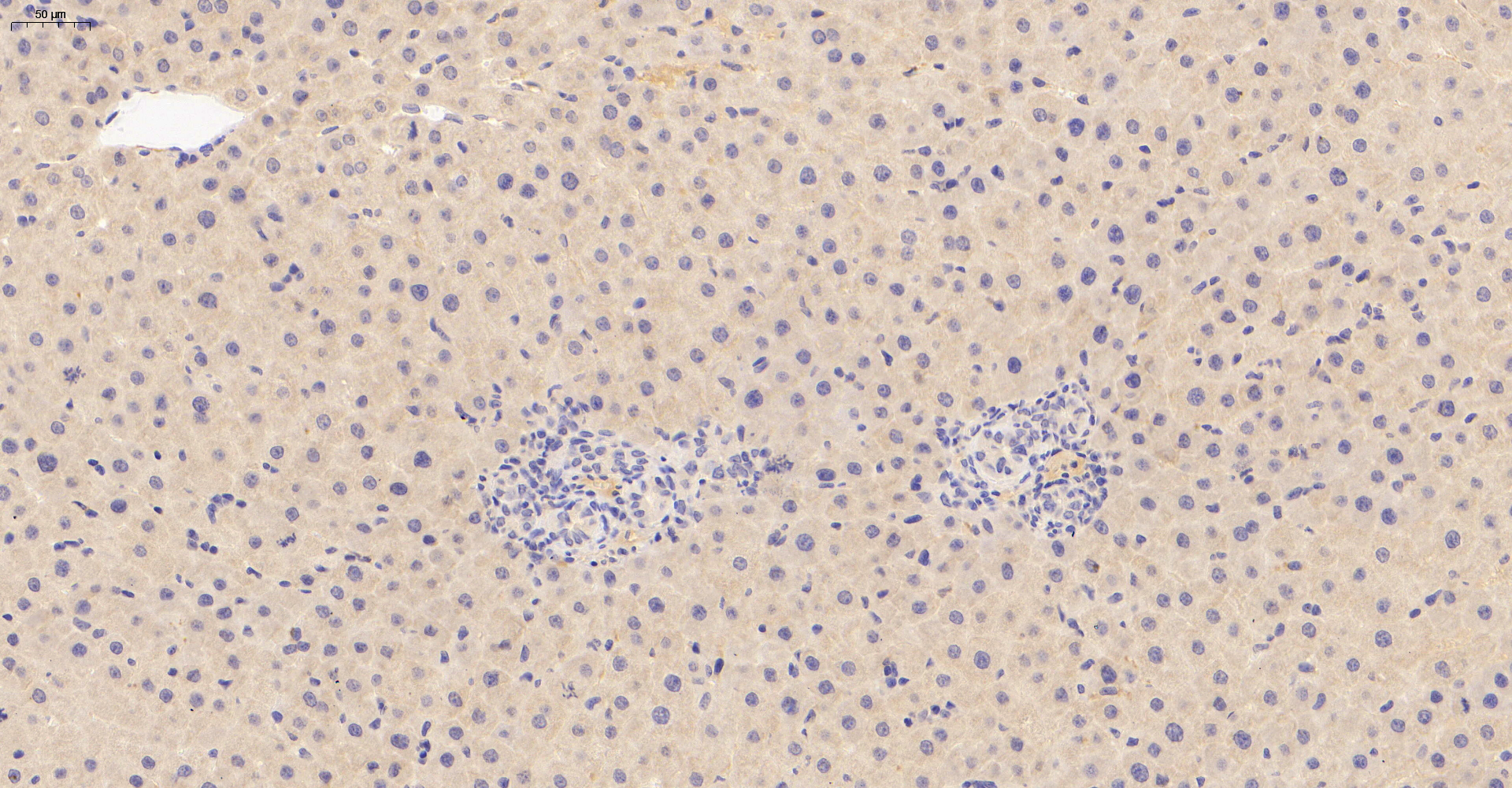

Supplement: Supplementary file 4 [file Data_Sheet_4.ZIP › IHC/miR-183-5p antagomir BDL group.tif]

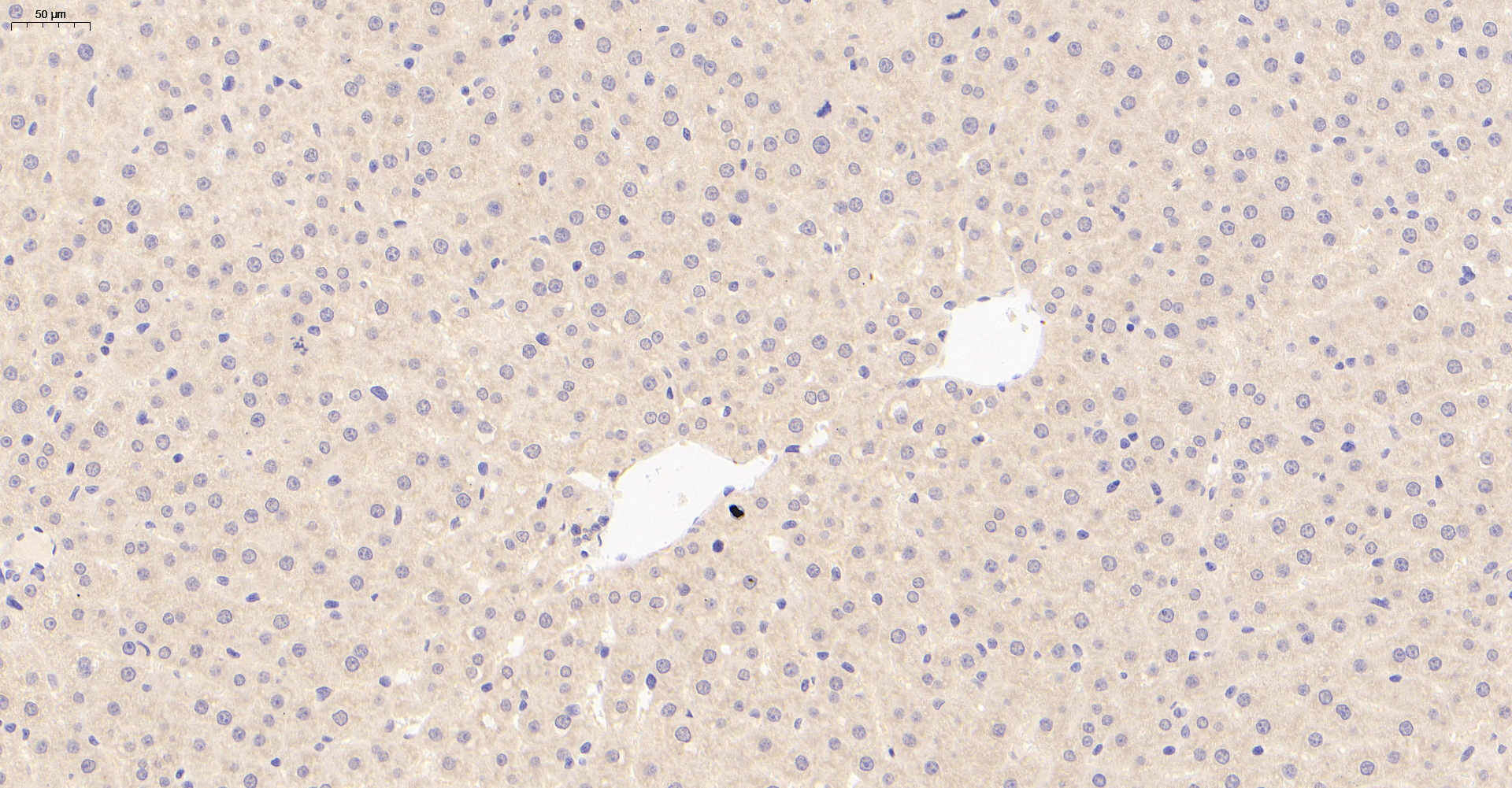

Supplement: Supplementary file 4 [file Data_Sheet_4.ZIP › IHC/miR-183-5p antagomir Sham group.tif]

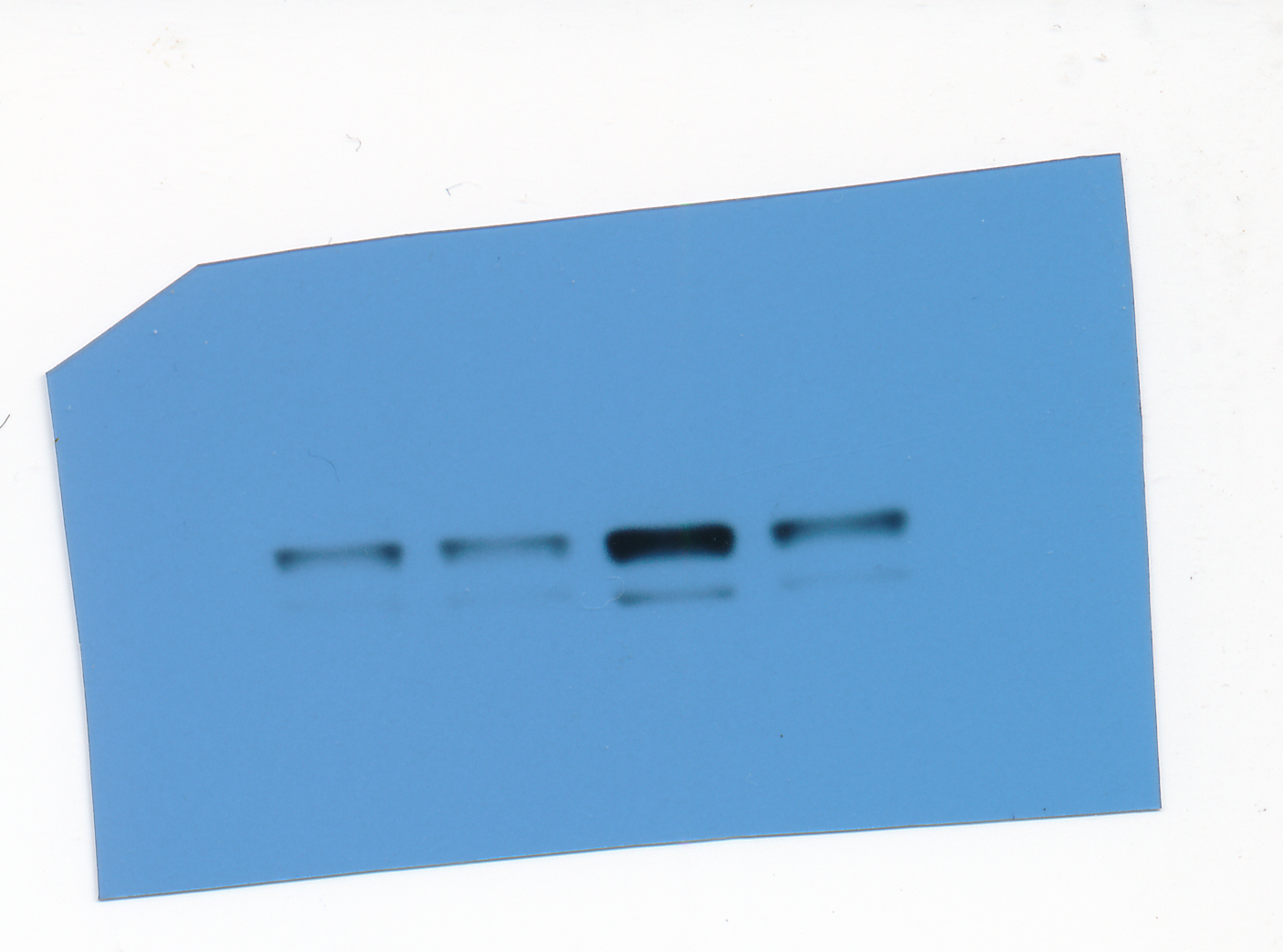

Supplement: Supplementary file 5 [file Data_Sheet_5.ZIP › Figure2 original data/Figure 2H WB/a-SMA.tif]

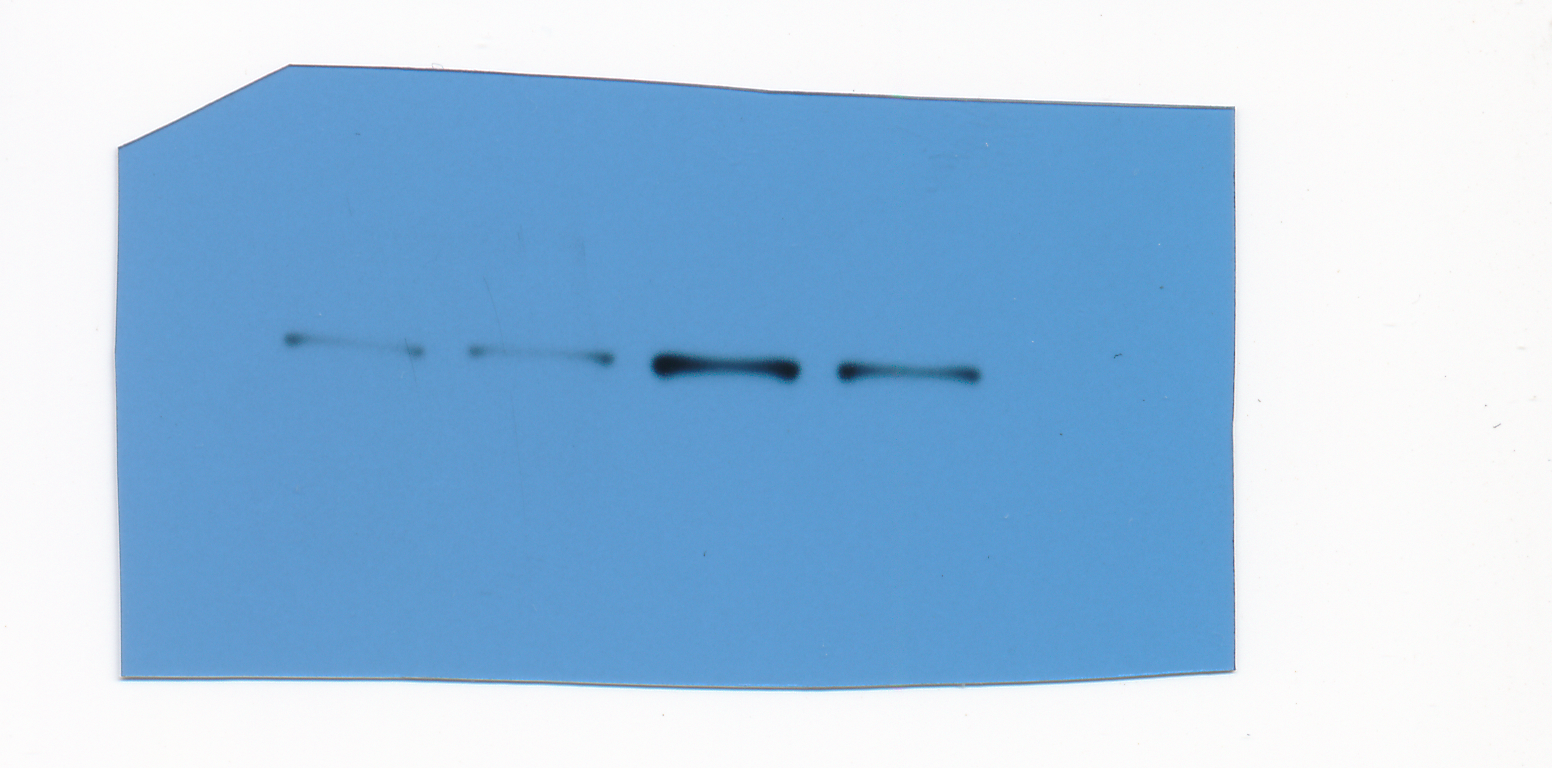

Supplement: Supplementary file 5 [file Data_Sheet_5.ZIP › Figure2 original data/Figure 2H WB/Collagen-I.tif]

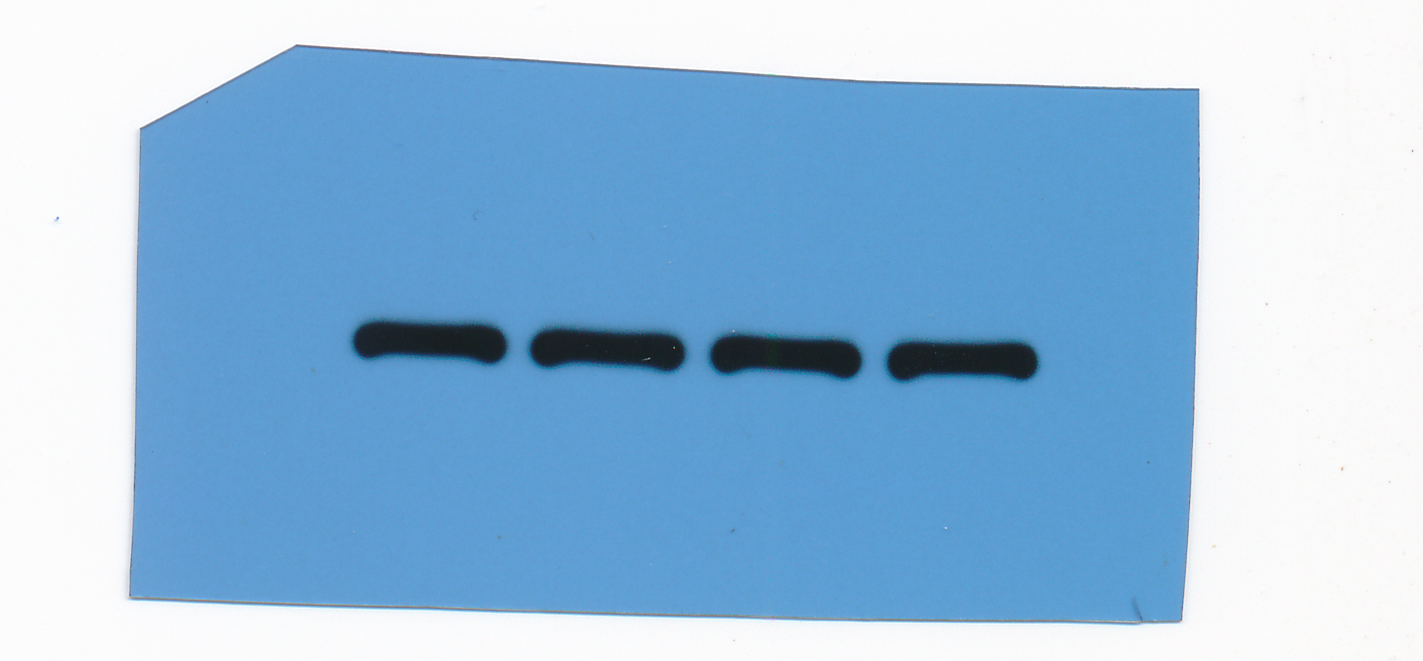

Supplement: Supplementary file 5 [file Data_Sheet_5.ZIP › Figure2 original data/Figure 2H WB/GAPDH.tif]

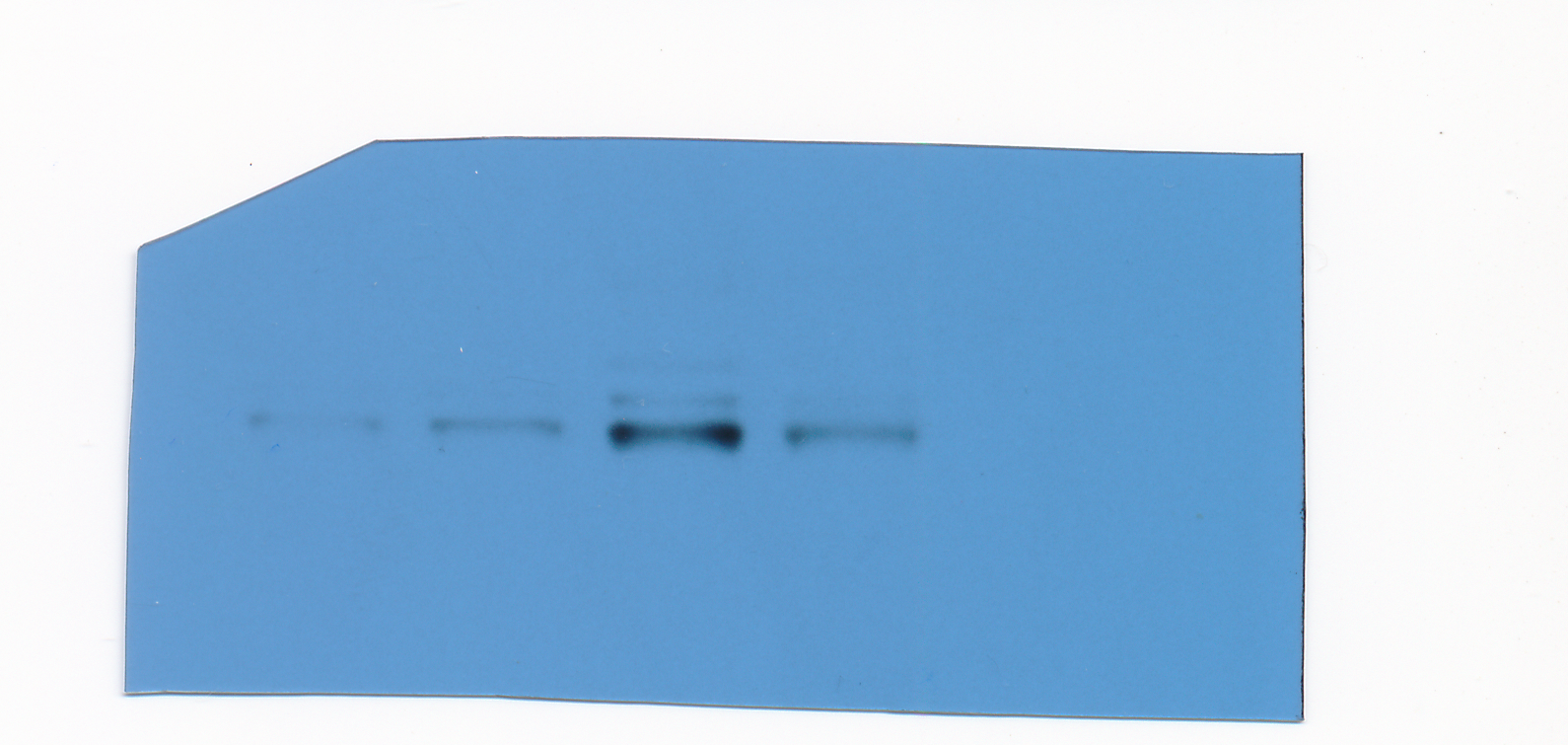

Supplement: Supplementary file 5 [file Data_Sheet_5.ZIP › Figure2 original data/Figure 2H WB/TIMP-1.tif]

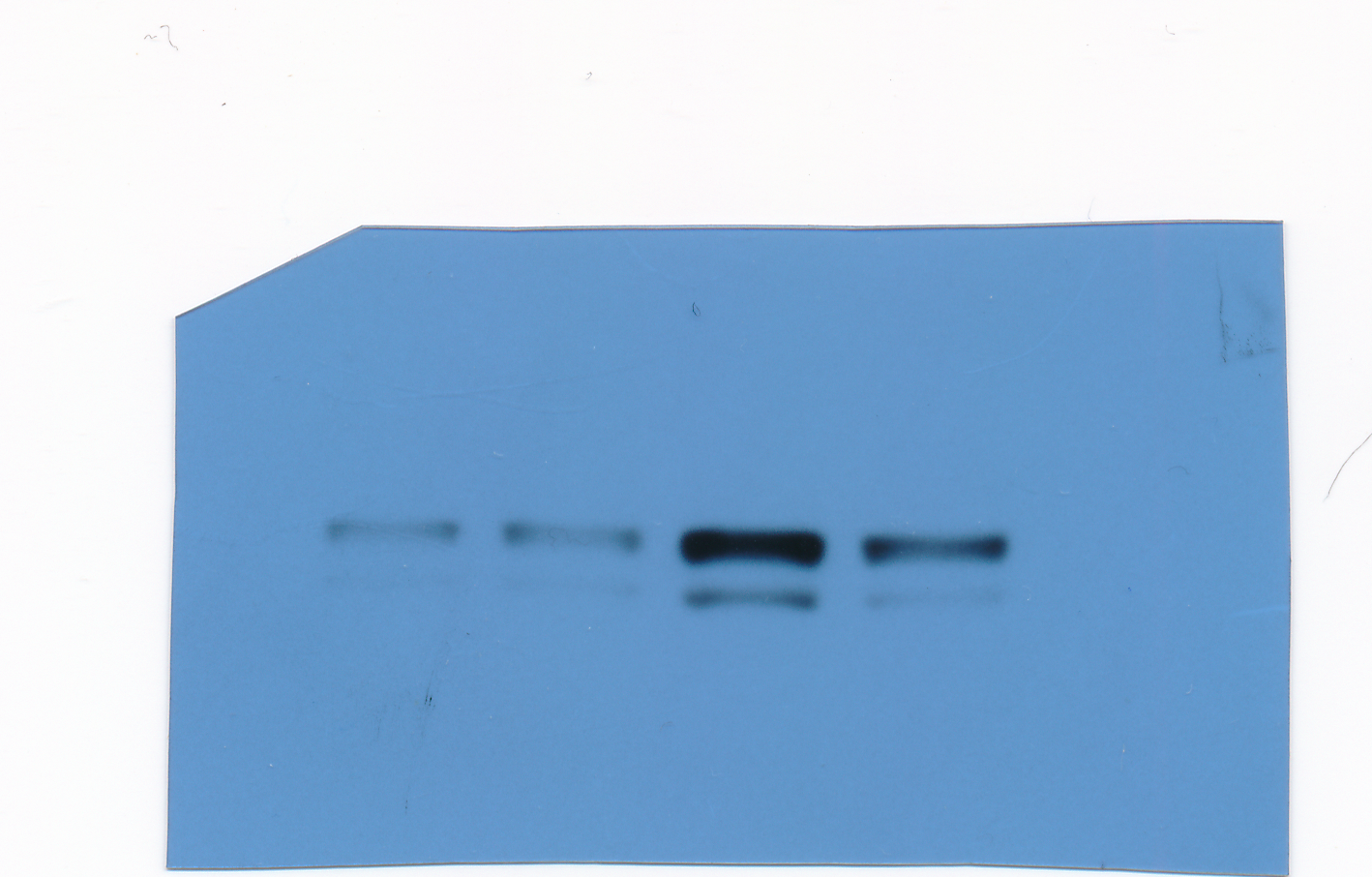

Supplement: Supplementary file 6 [file Data_Sheet_6.ZIP › Figure3 original data/Figure 3D WB/a-SMA.tif]

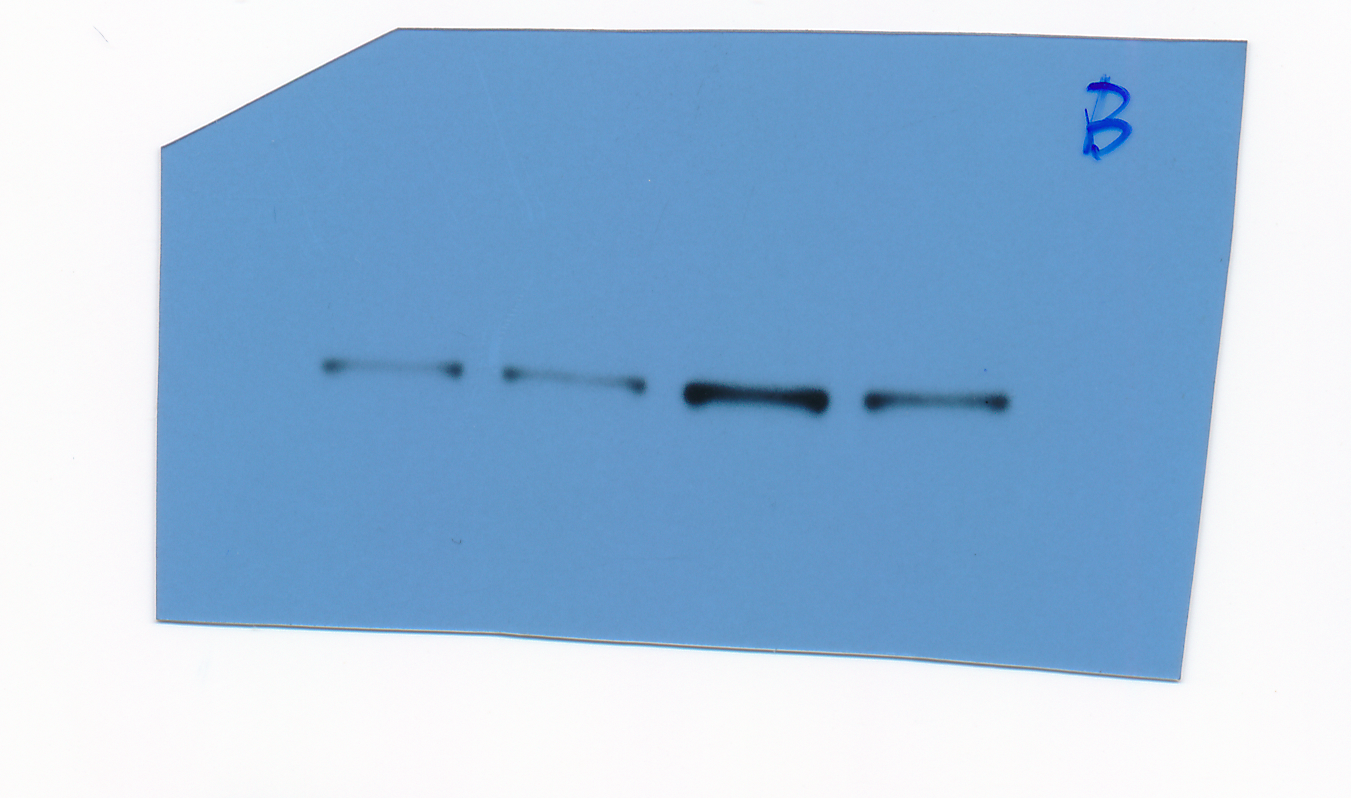

Supplement: Supplementary file 6 [file Data_Sheet_6.ZIP › Figure3 original data/Figure 3D WB/Collagen-I.tif]

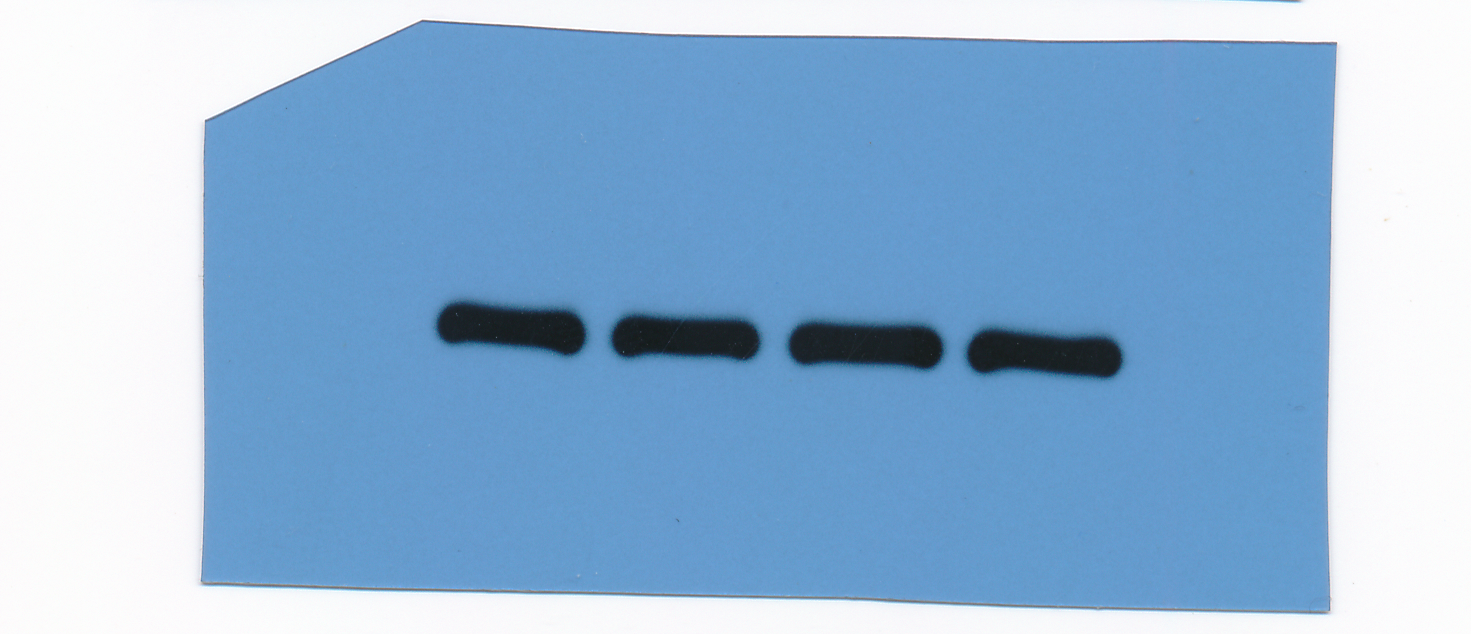

Supplement: Supplementary file 6 [file Data_Sheet_6.ZIP › Figure3 original data/Figure 3D WB/GAPDH.tif]

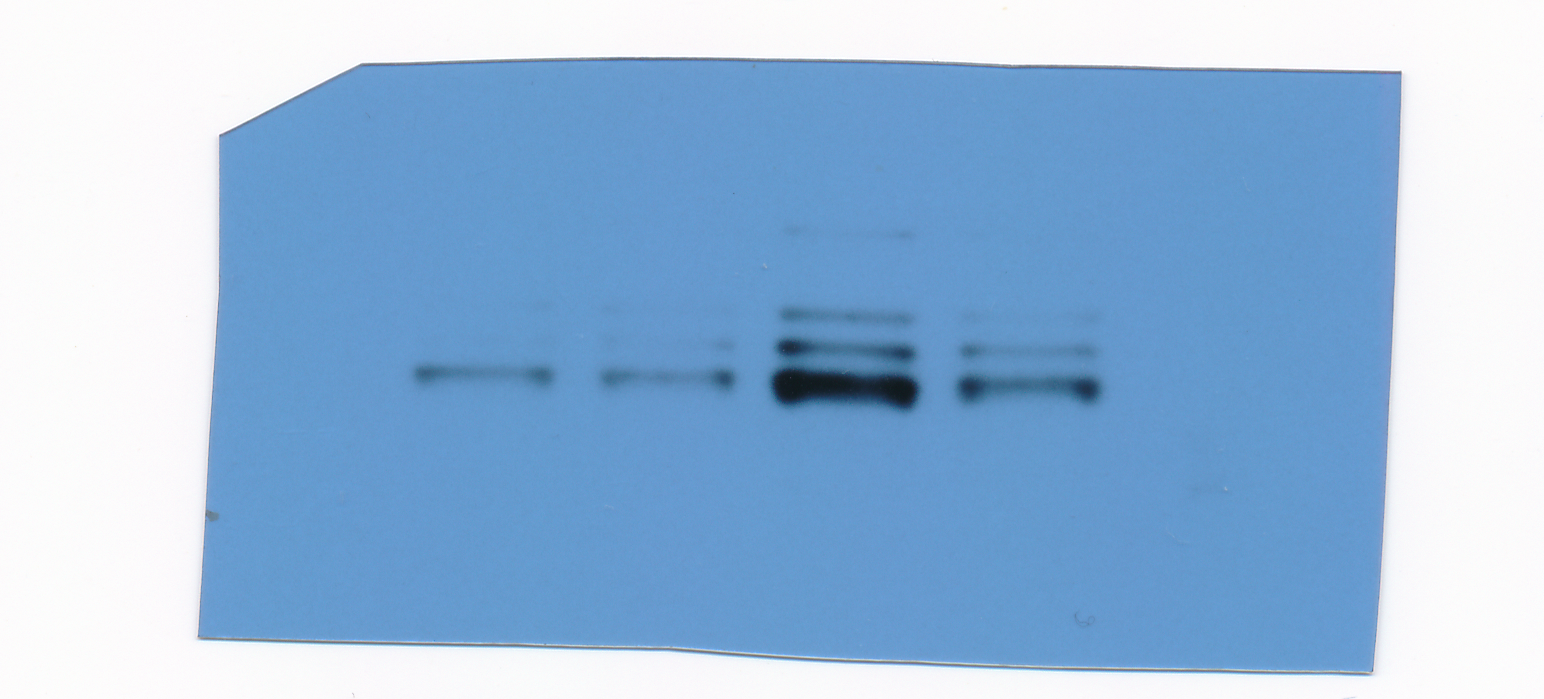

Supplement: Supplementary file 6 [file Data_Sheet_6.ZIP › Figure3 original data/Figure 3D WB/TIMP-1.tif]

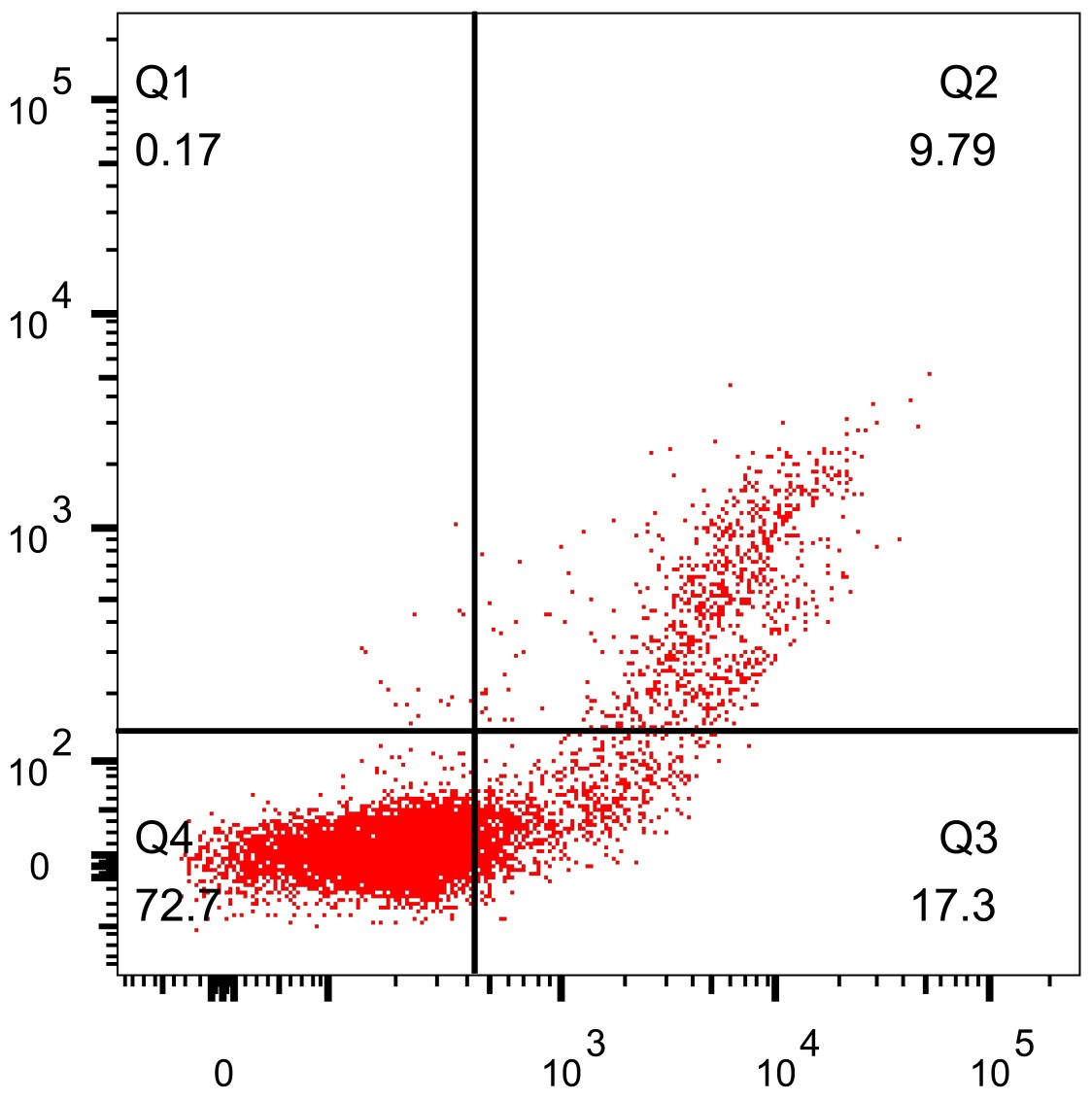

Supplement: Supplementary file 6 [file Data_Sheet_6.ZIP › Figure3 original data/Figure 3F/control antagomir con group.jpg]

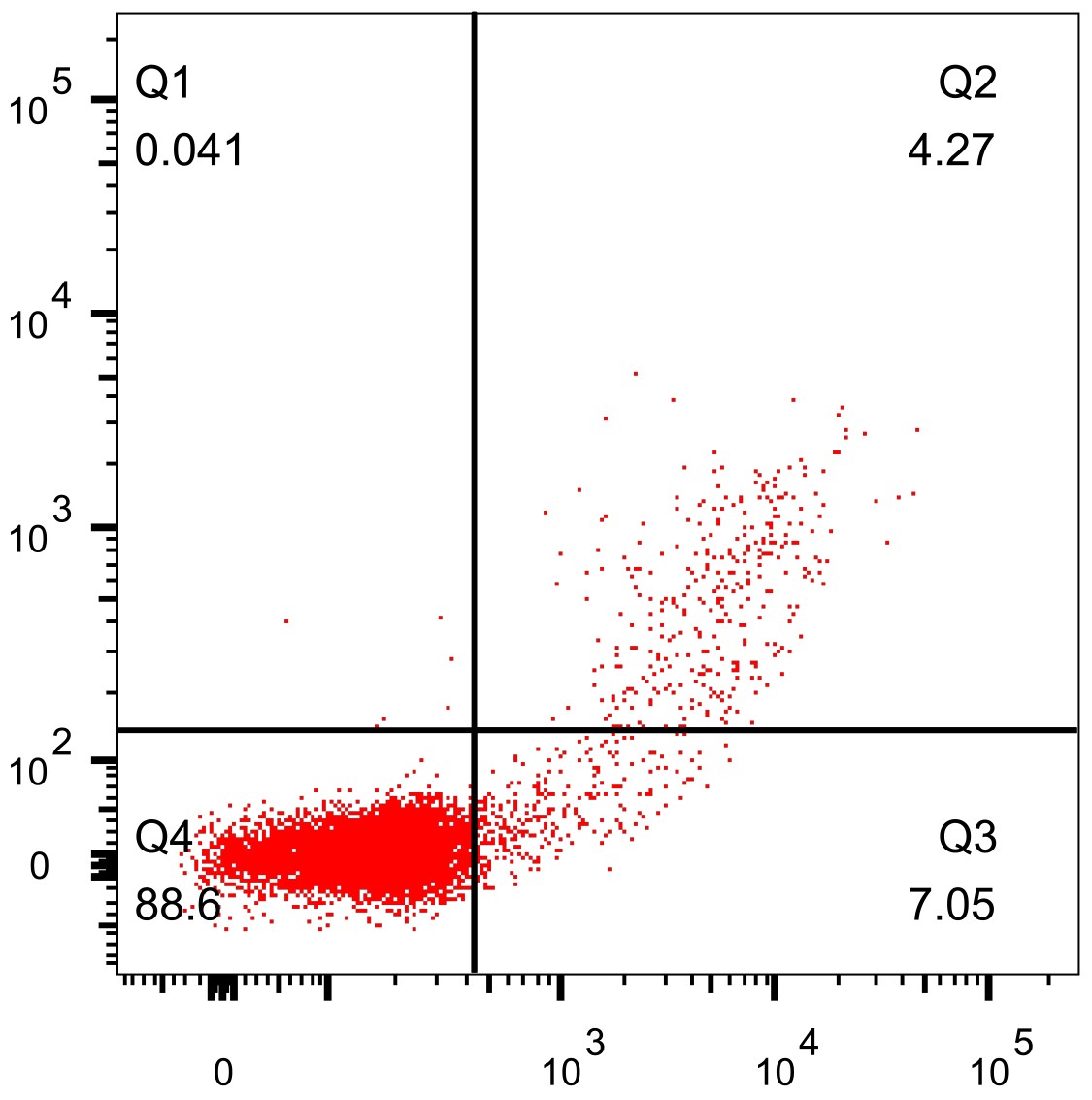

Supplement: Supplementary file 6 [file Data_Sheet_6.ZIP › Figure3 original data/Figure 3F/control antagomir TGF-a┬1 group.jpg]

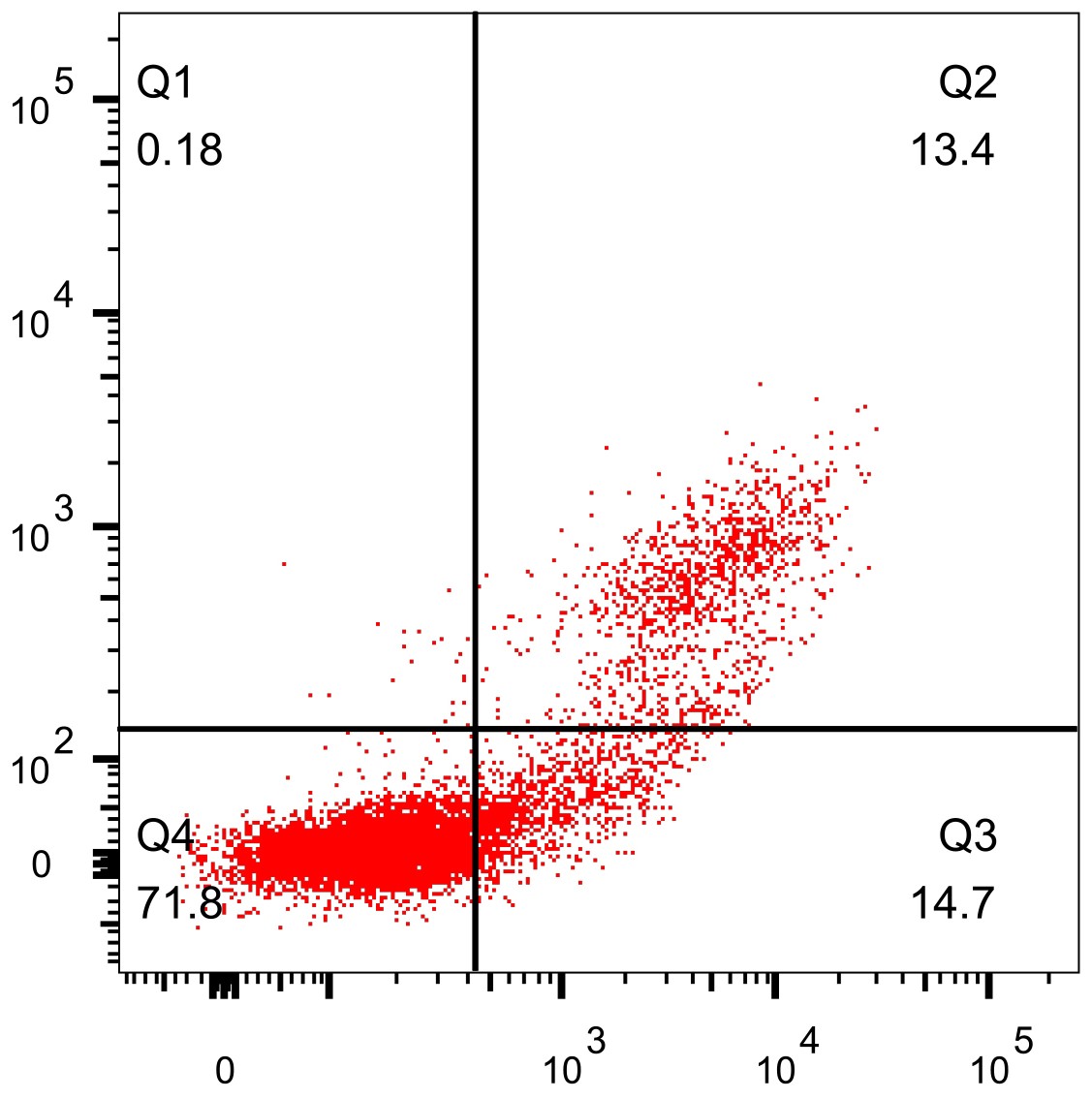

Supplement: Supplementary file 6 [file Data_Sheet_6.ZIP › Figure3 original data/Figure 3F/miR-183-5p antagomir con group.jpg]

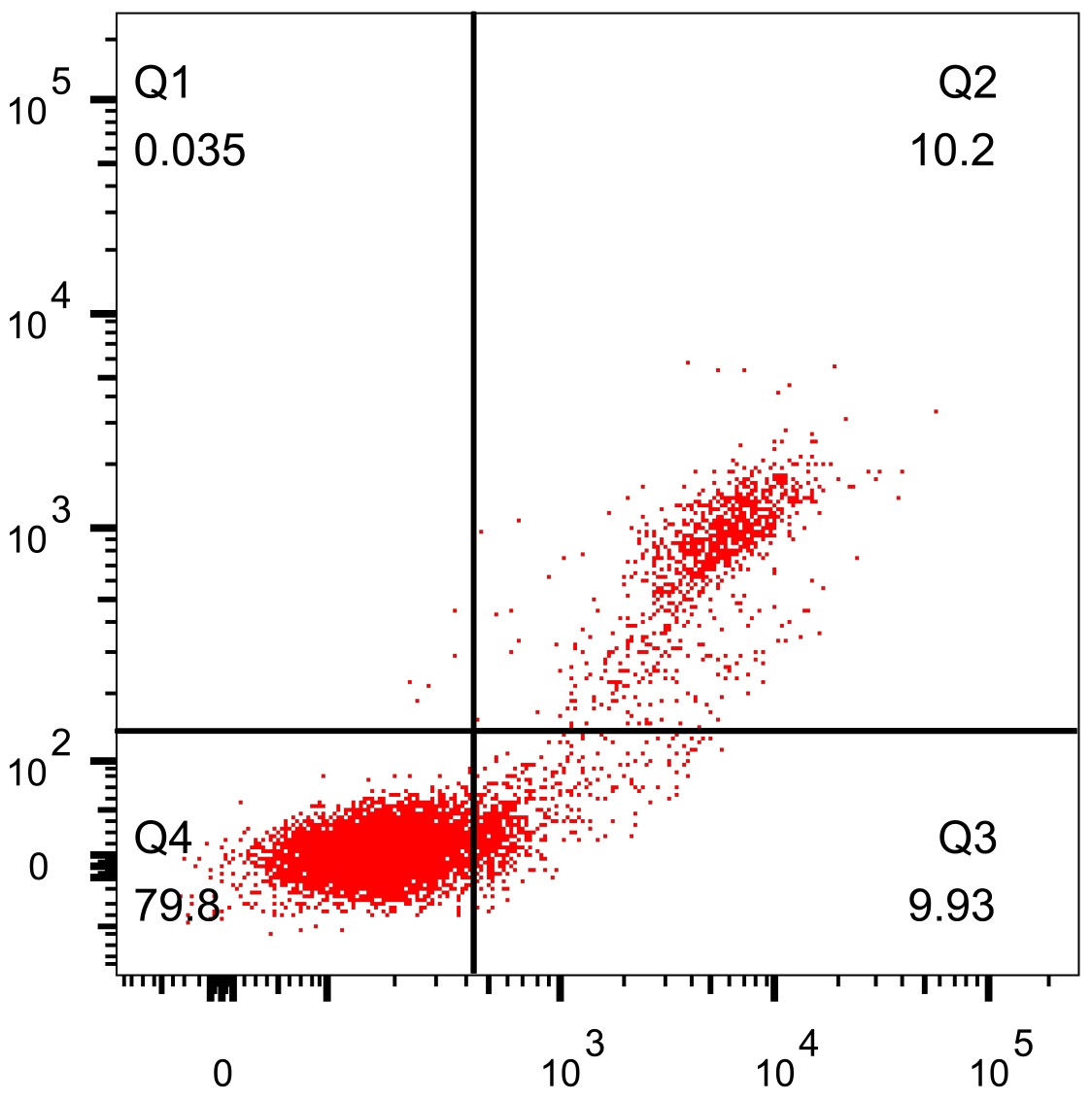

Supplement: Supplementary file 6 [file Data_Sheet_6.ZIP › Figure3 original data/Figure 3F/miR-183-5p antagomir TGF-a┬1 group.jpg]

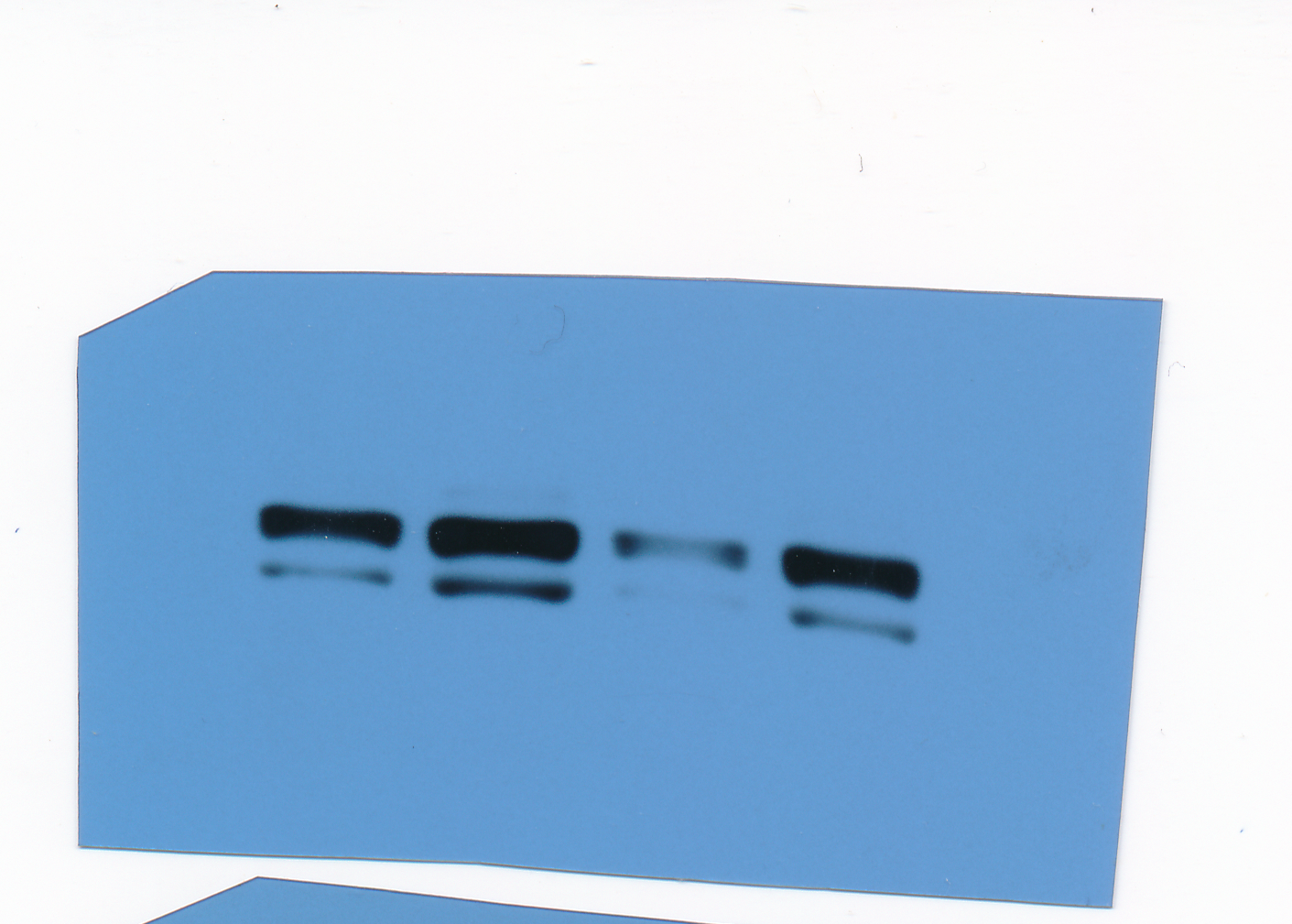

Supplement: Supplementary file 8 [file Data_Sheet_8.ZIP › Figure5 original data/Figure 5D WB/a-SMA.tif]

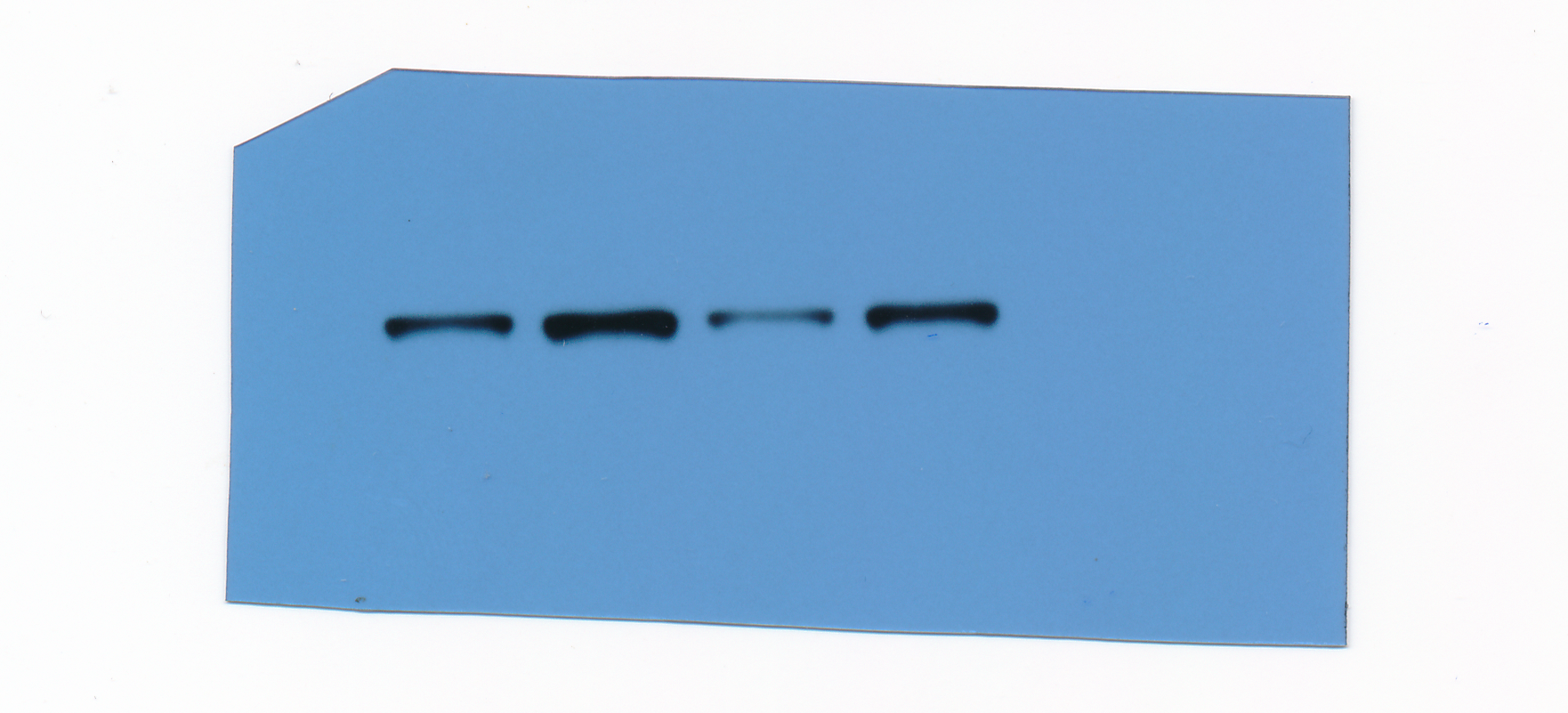

Supplement: Supplementary file 8 [file Data_Sheet_8.ZIP › Figure5 original data/Figure 5D WB/Collagen-I.tif]

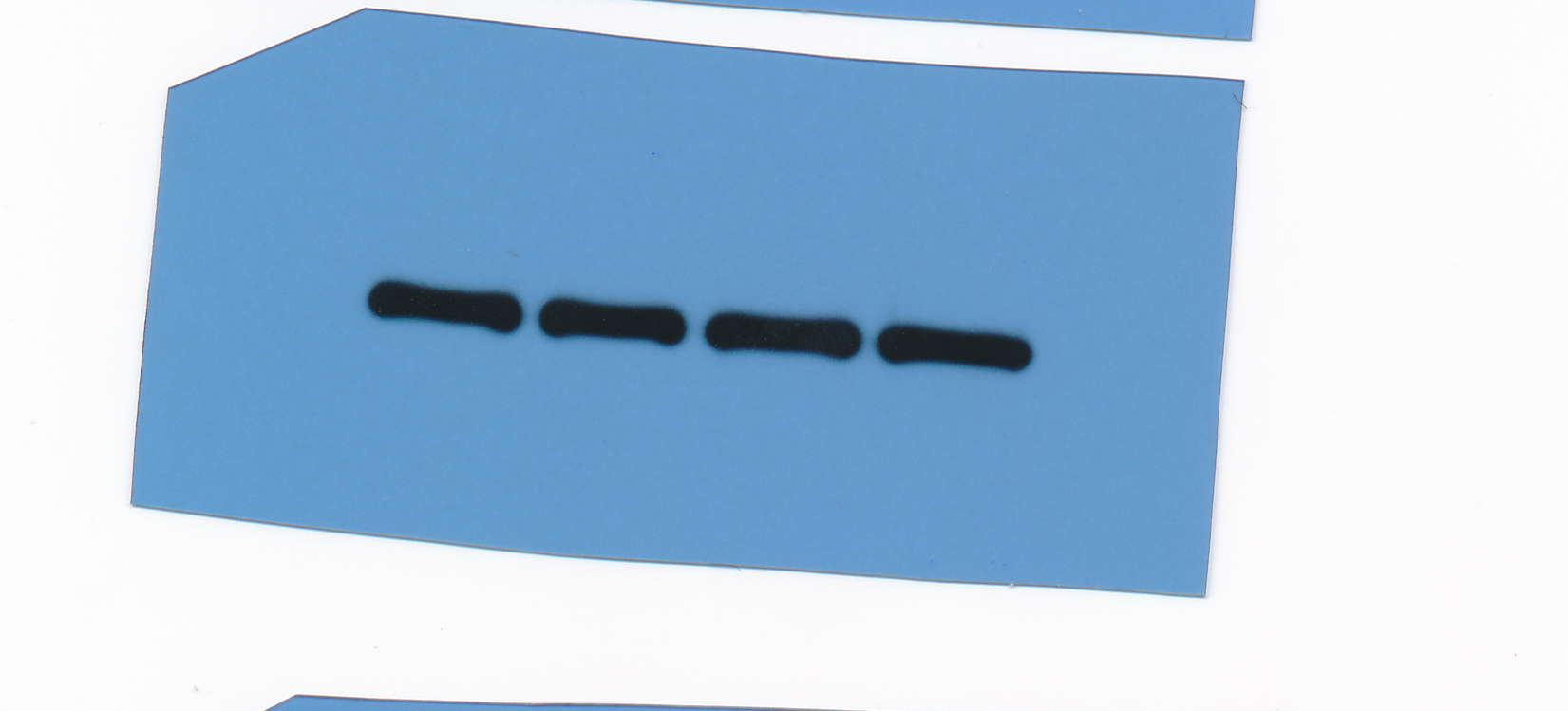

Supplement: Supplementary file 8 [file Data_Sheet_8.ZIP › Figure5 original data/Figure 5D WB/GAPDH.tif]

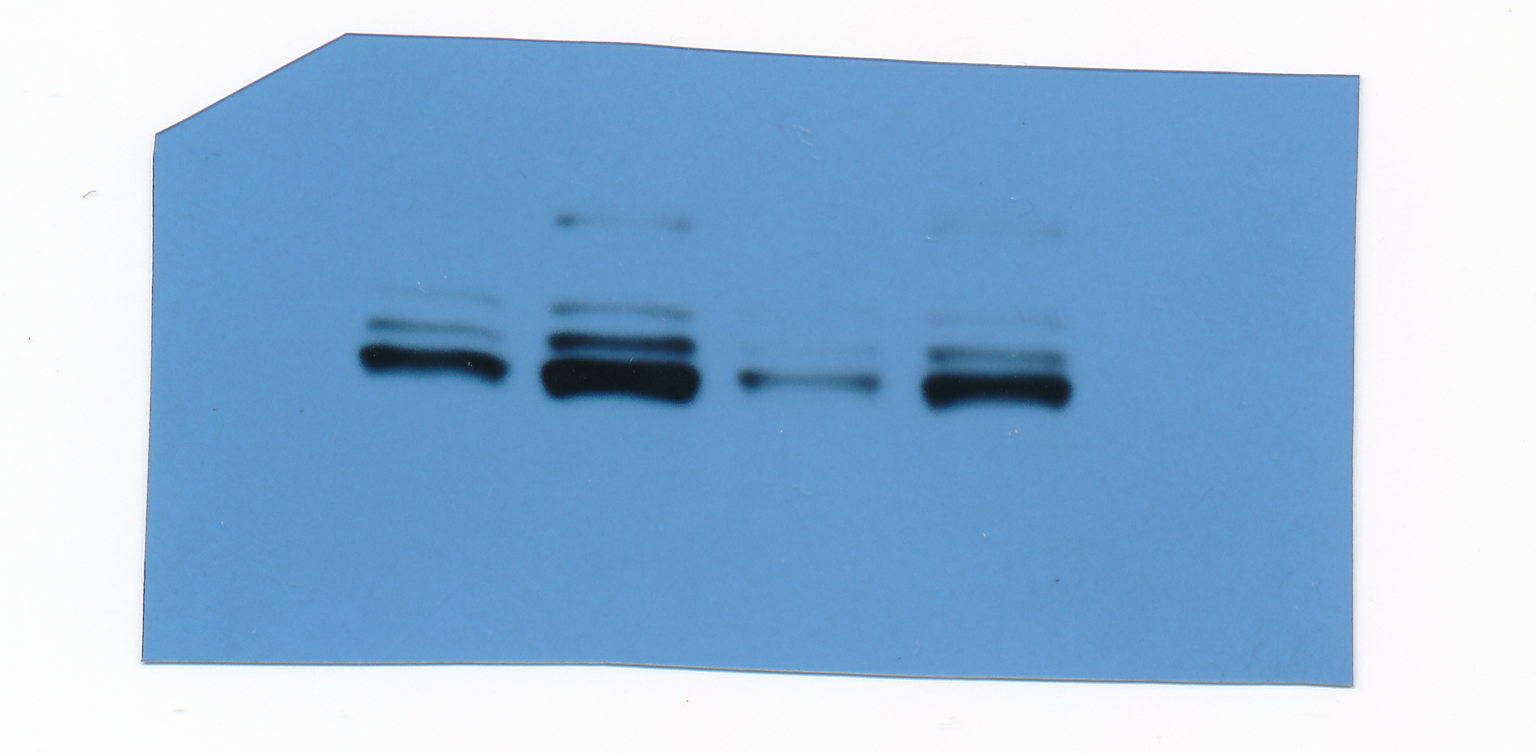

Supplement: Supplementary file 8 [file Data_Sheet_8.ZIP › Figure5 original data/Figure 5D WB/TIMP-1.tif]

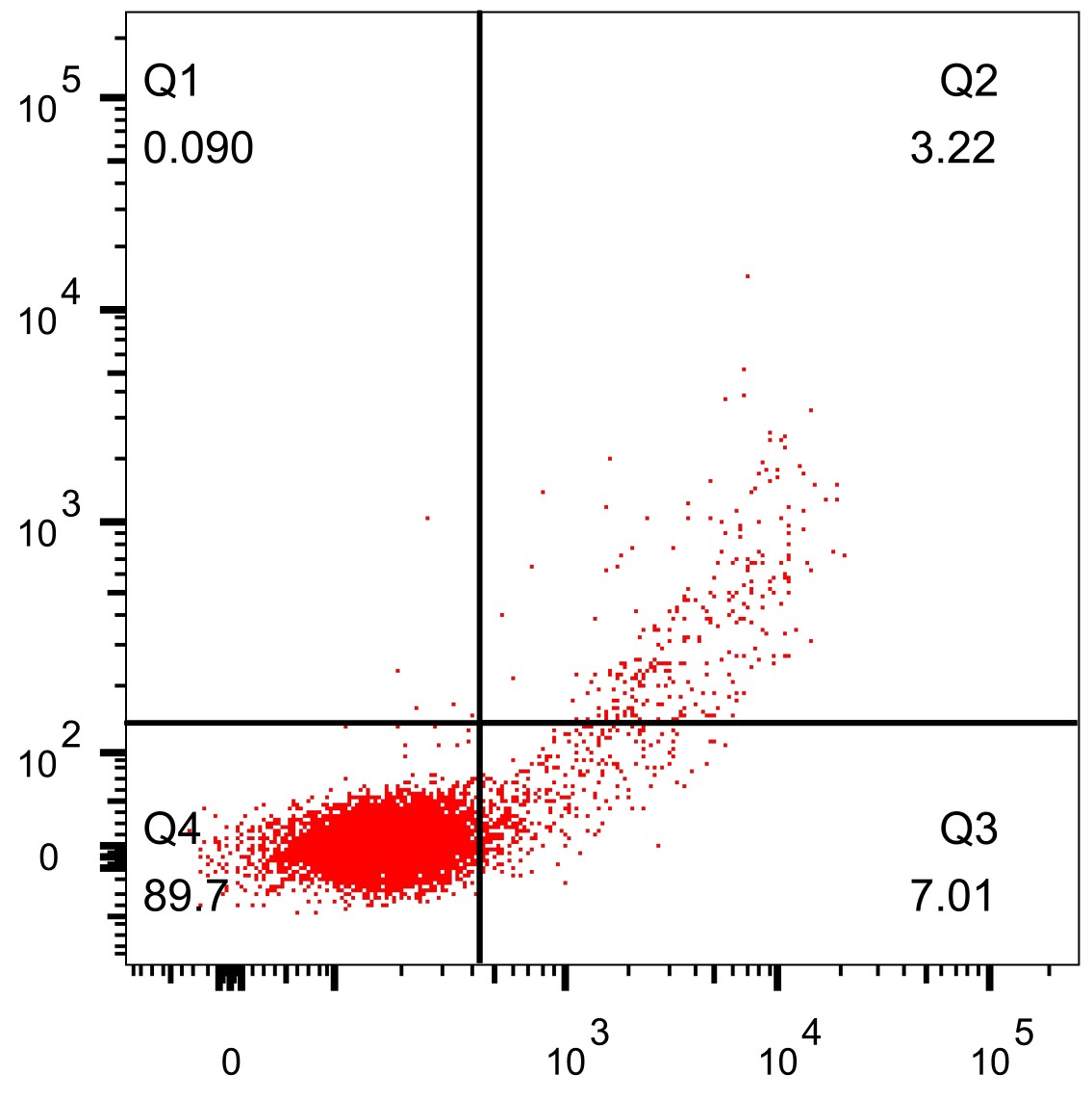

Supplement: Supplementary file 8 [file Data_Sheet_8.ZIP › Figure5 original data/Figure 5F/control agomir group.jpg]

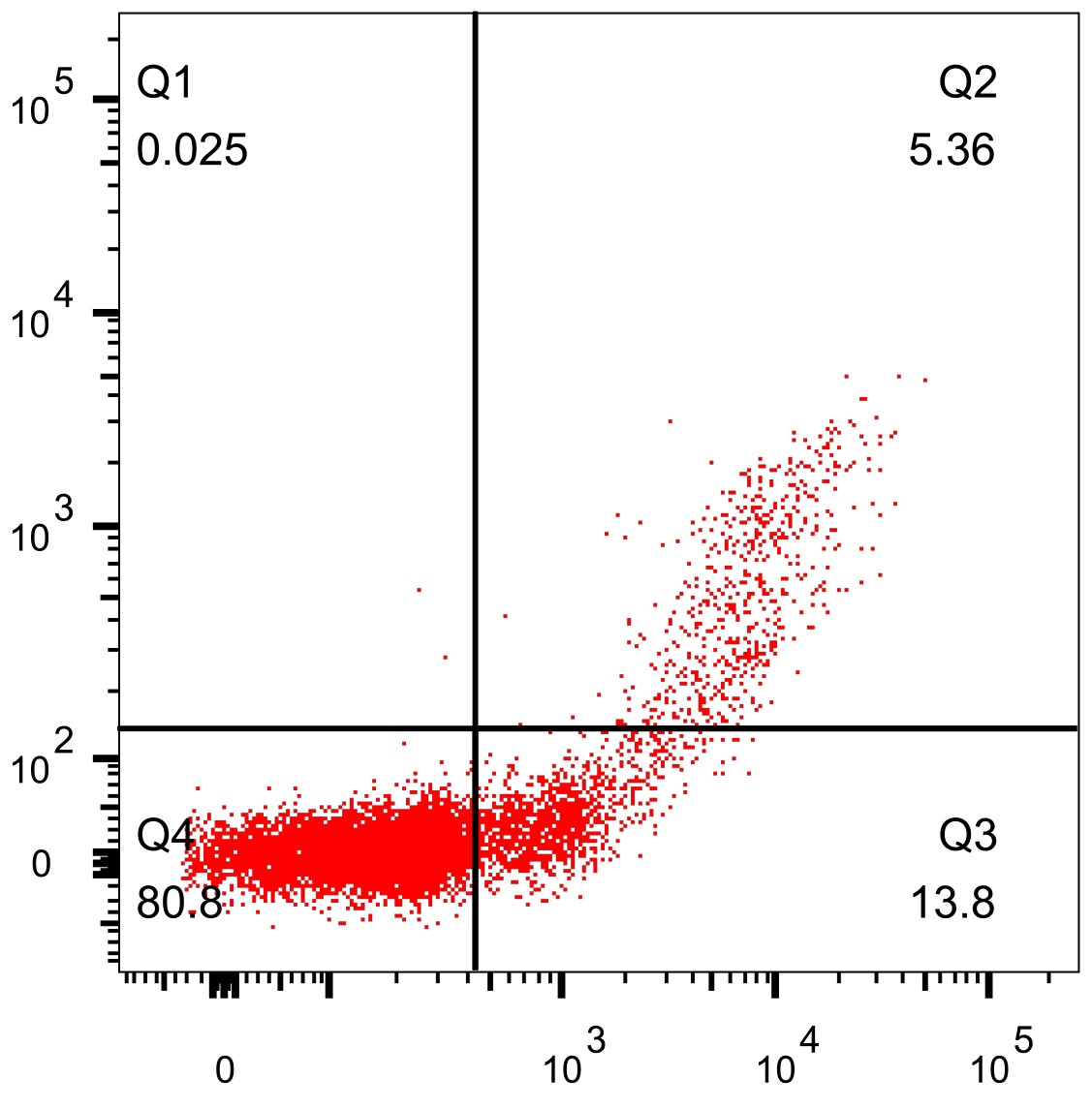

Supplement: Supplementary file 8 [file Data_Sheet_8.ZIP › Figure5 original data/Figure 5F/control agomir LV-FOXO1 group.jpg]

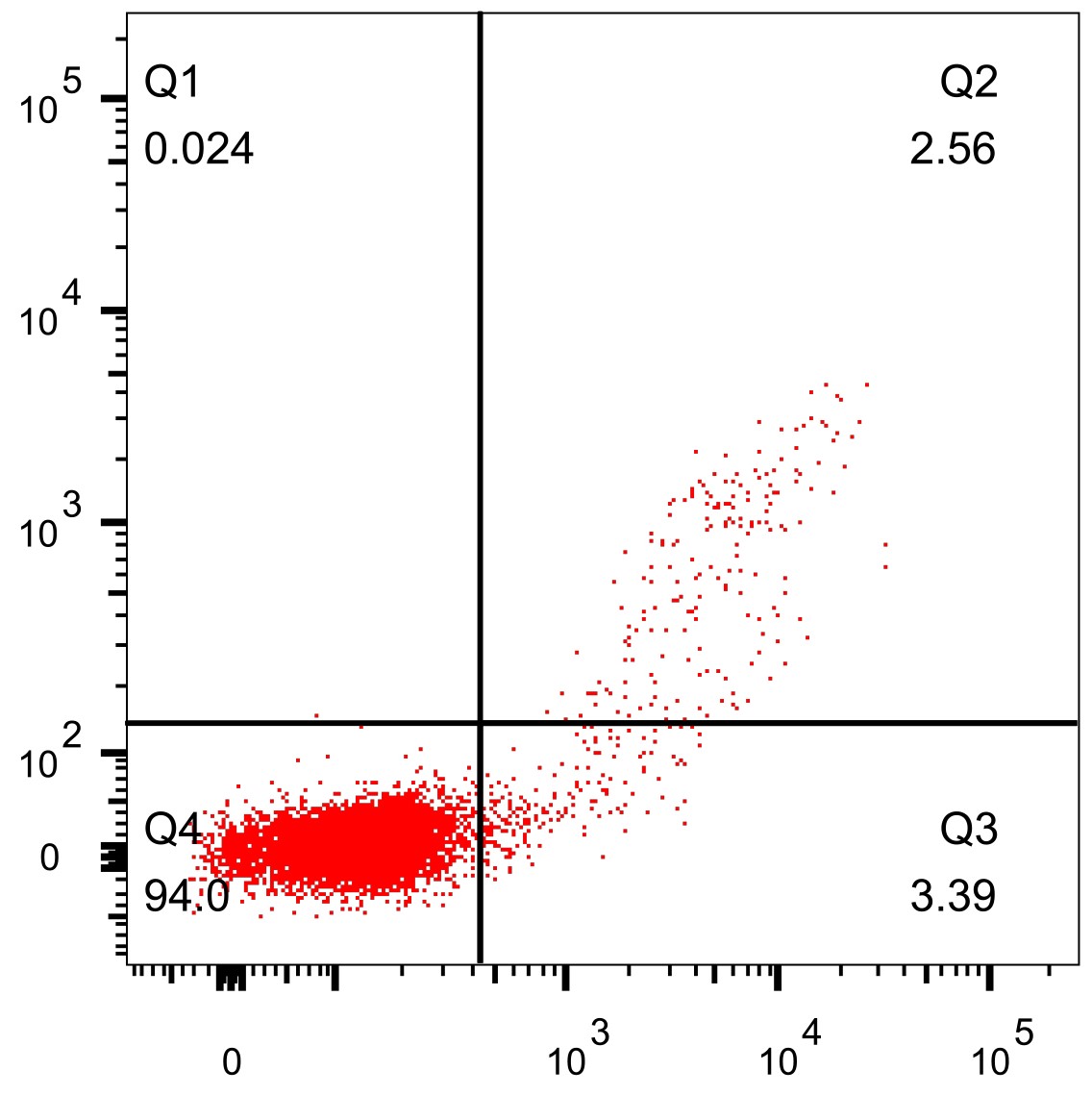

Supplement: Supplementary file 8 [file Data_Sheet_8.ZIP › Figure5 original data/Figure 5F/miR-183-5p agomir group.jpg]

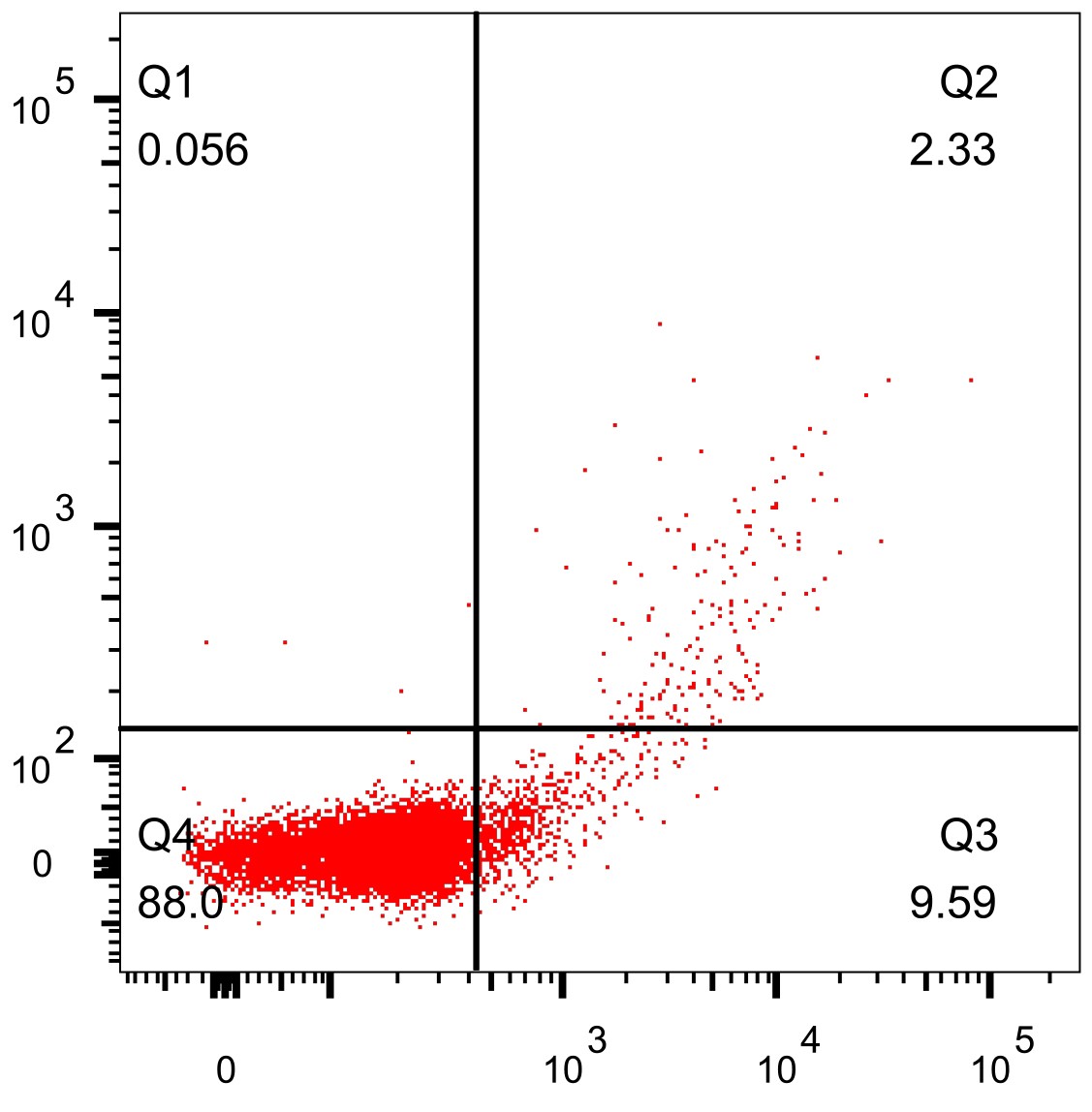

Supplement: Supplementary file 8 [file Data_Sheet_8.ZIP › Figure5 original data/Figure 5F/miR-183-5p agomir LV-FOXO1 group.jpg]

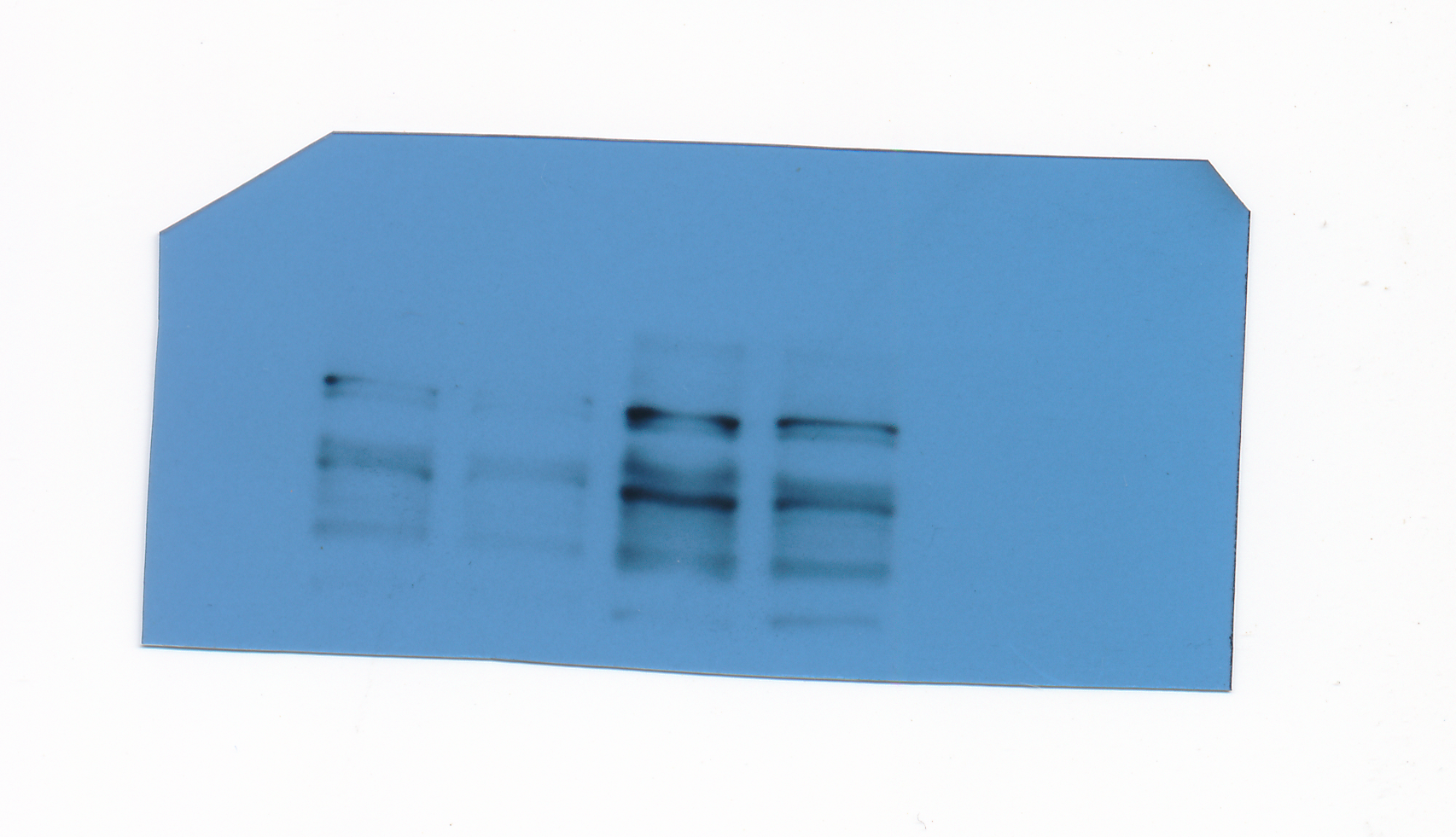

Supplement: Supplementary file 8 [file Data_Sheet_8.ZIP › Figure5 original data/Figure 5I WB/FOXO1.tif]

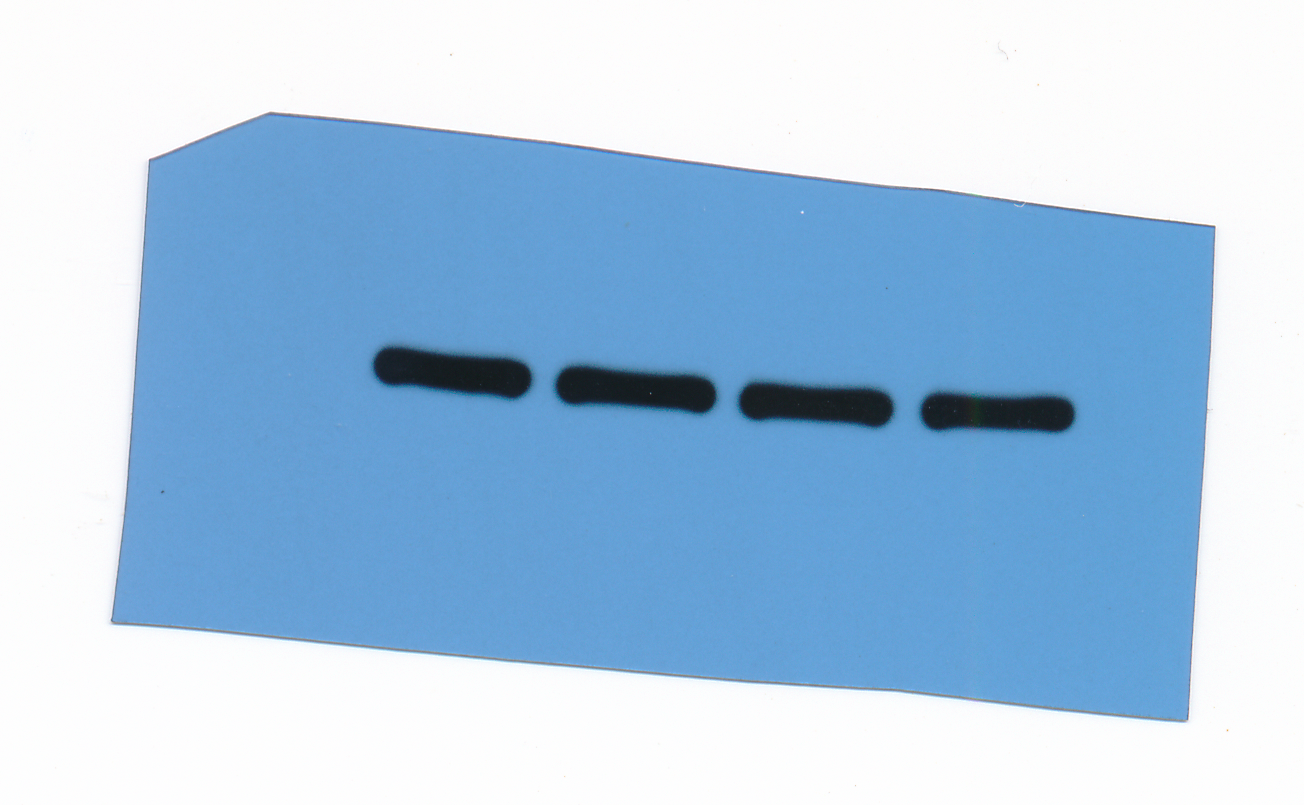

Supplement: Supplementary file 8 [file Data_Sheet_8.ZIP › Figure5 original data/Figure 5I WB/GAPDH.tif]

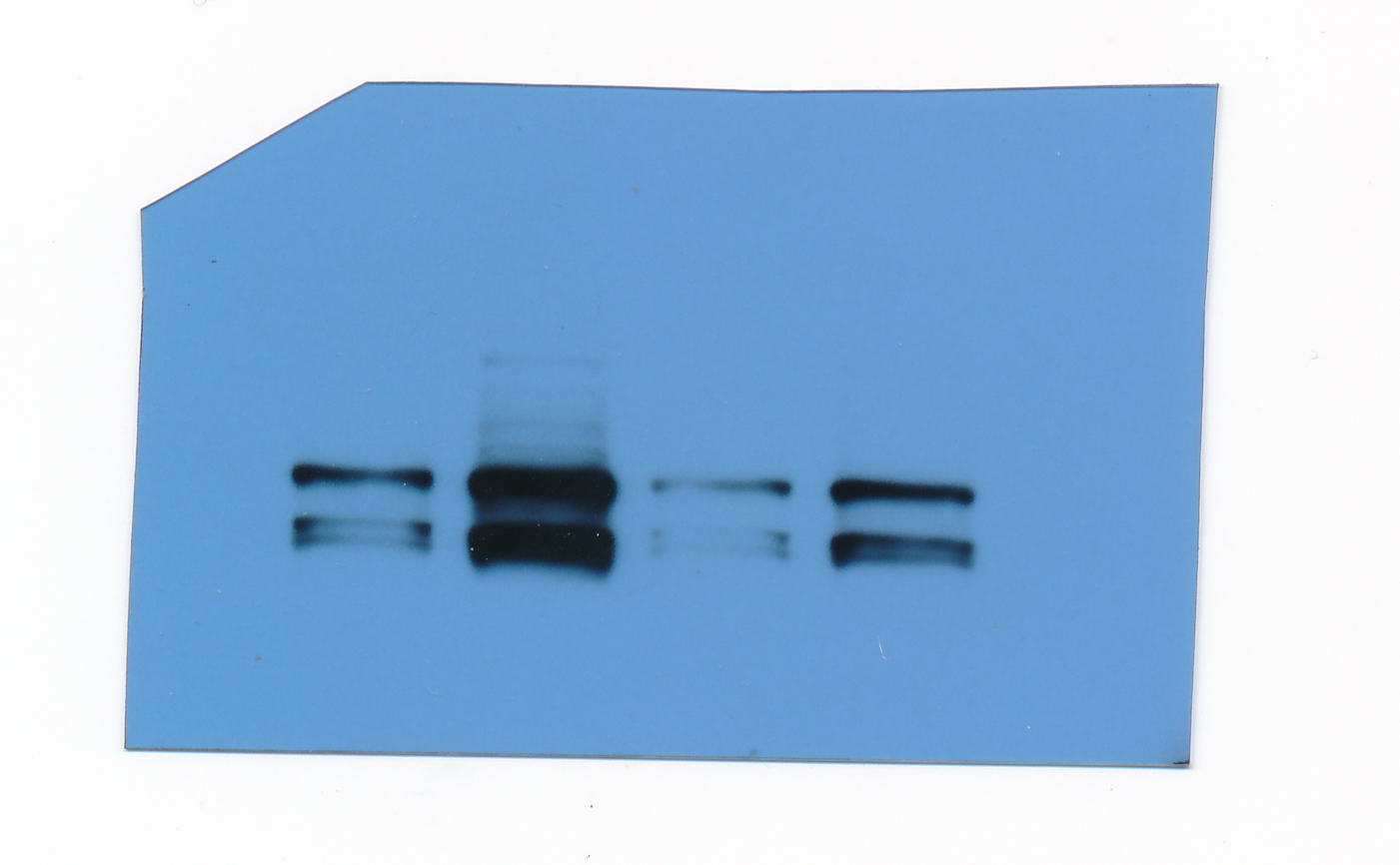

Supplement: Supplementary file 8 [file Data_Sheet_8.ZIP › Figure5 original data/Figure 5I WB/p-smad23.tif]

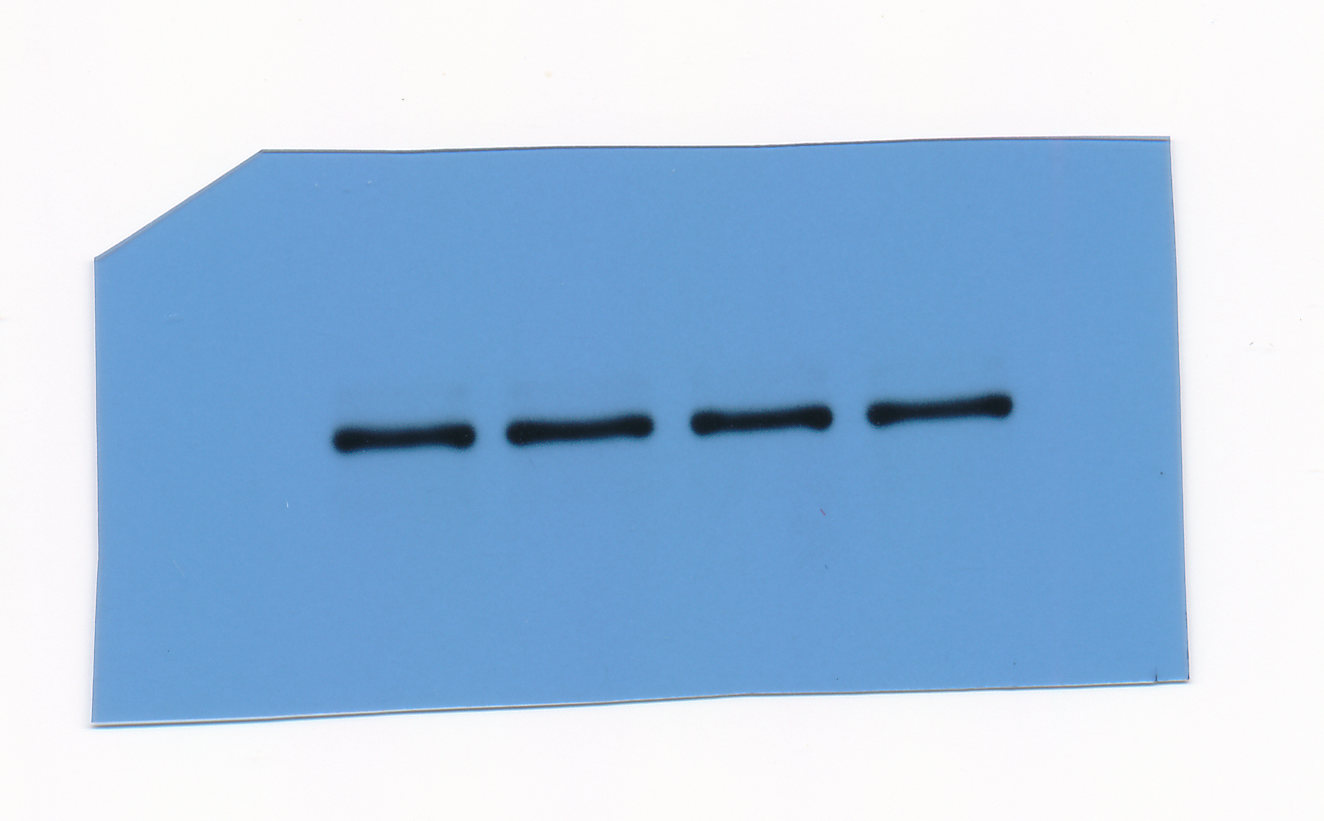

Supplement: Supplementary file 8 [file Data_Sheet_8.ZIP › Figure5 original data/Figure 5I WB/Smad2.tif]

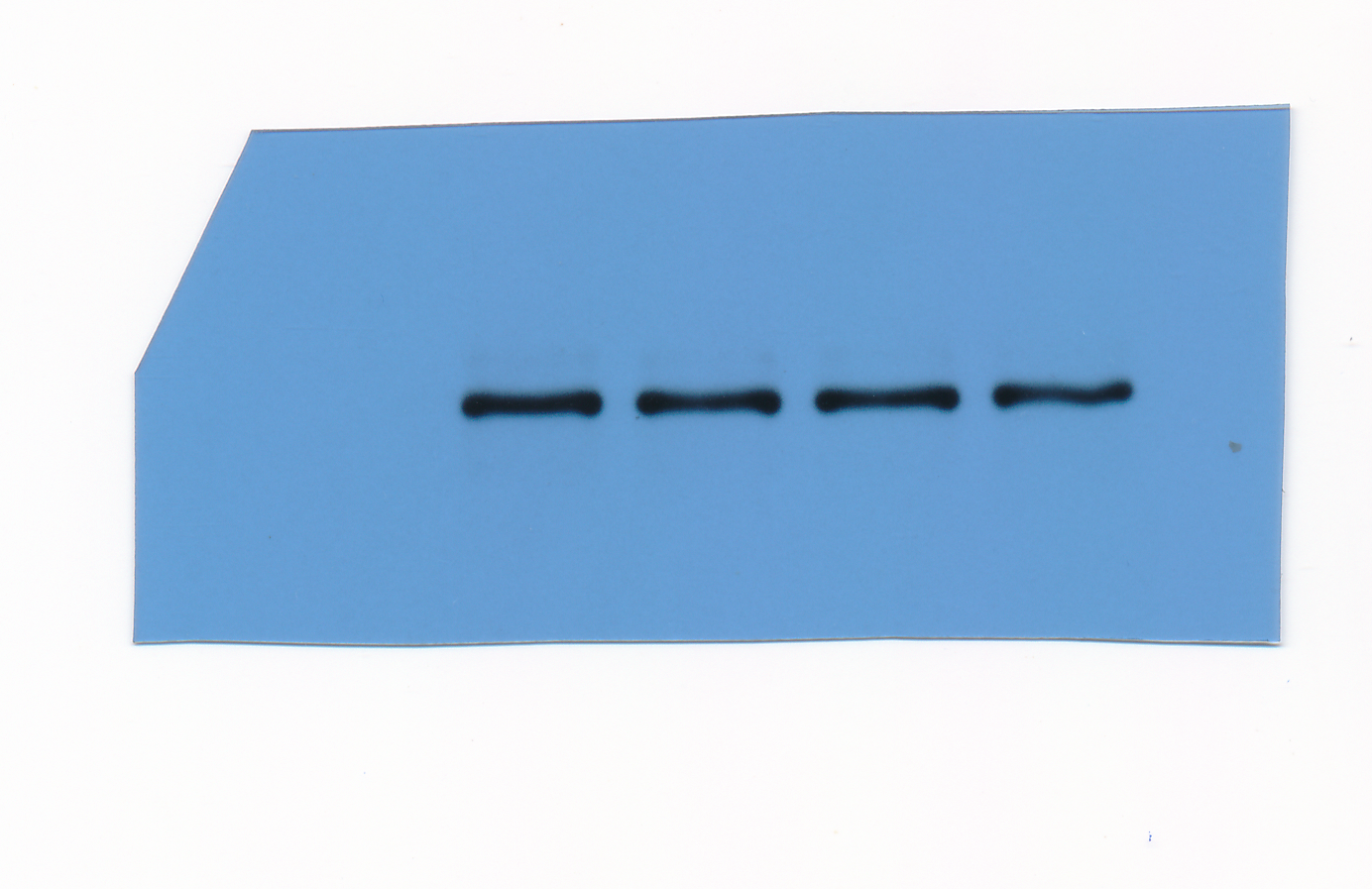

Supplement: Supplementary file 8 [file Data_Sheet_8.ZIP › Figure5 original data/Figure 5I WB/Smad3.tif]

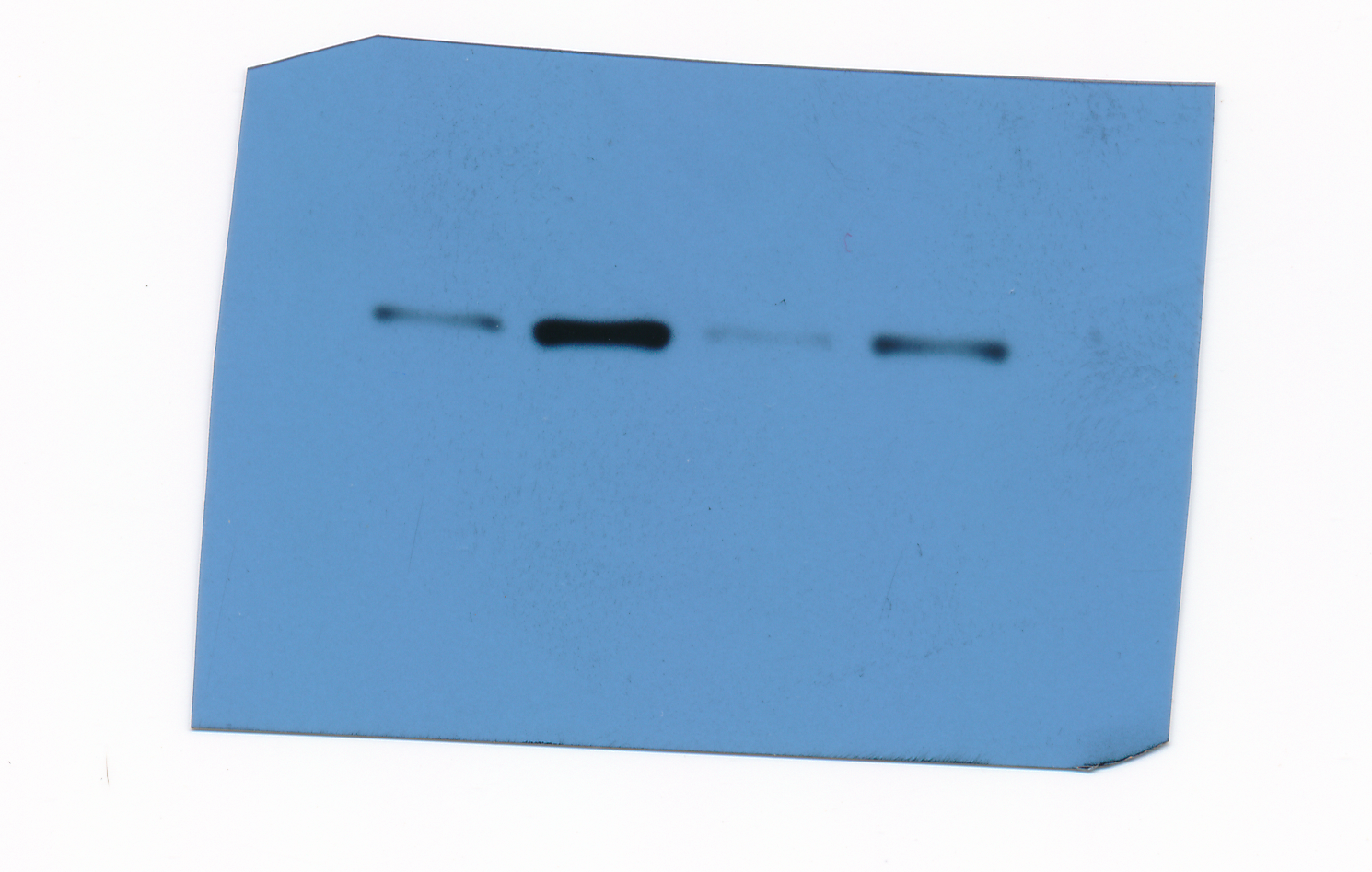

Supplement: Supplementary file 8 [file Data_Sheet_8.ZIP › Figure5 original data/Figure 5I WB/TGF-a┬.tif]

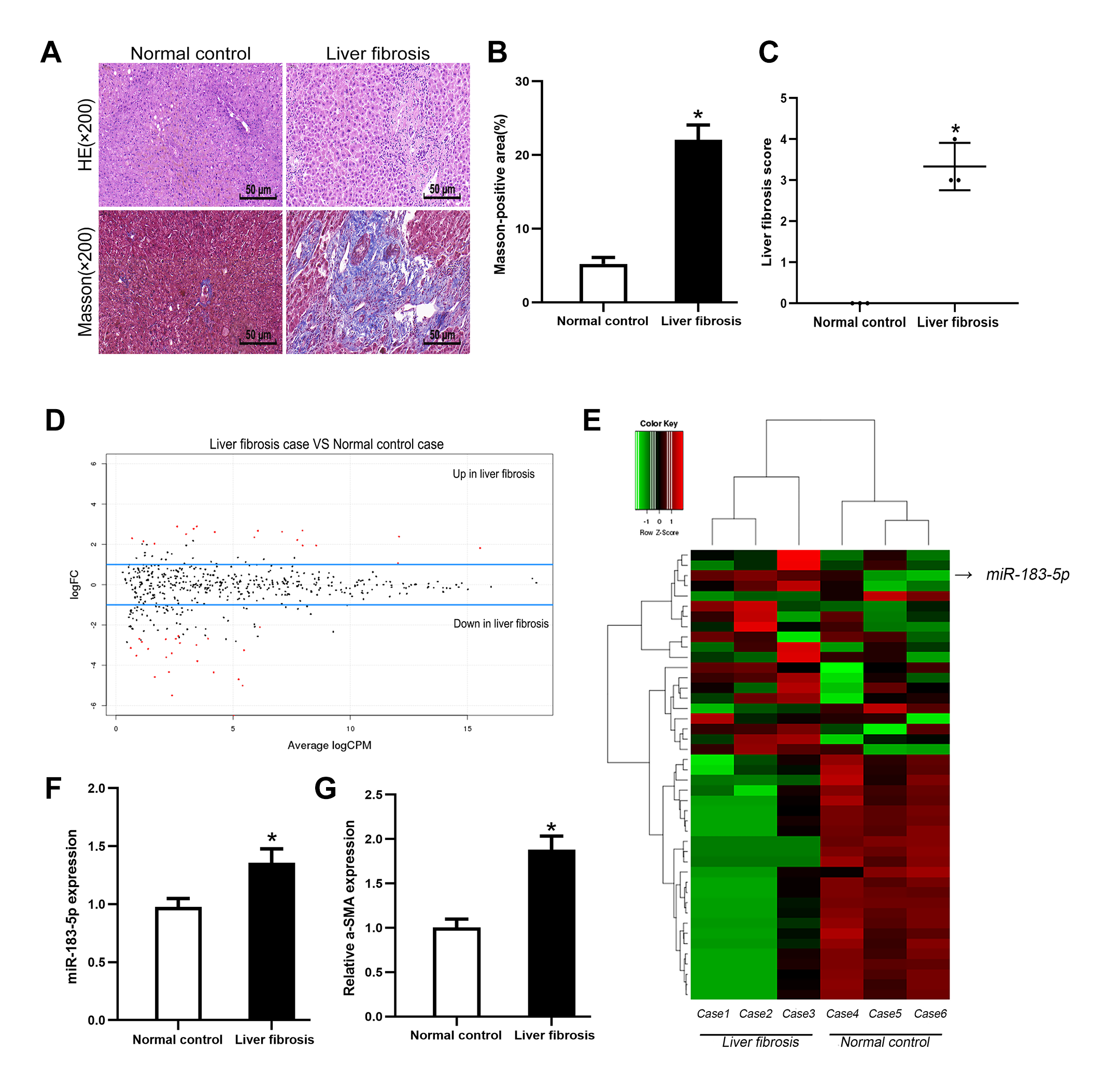

Supplement: Supplementary file 9 [file Data_Sheet_9.ZIP › Figure/Figure1.tif]

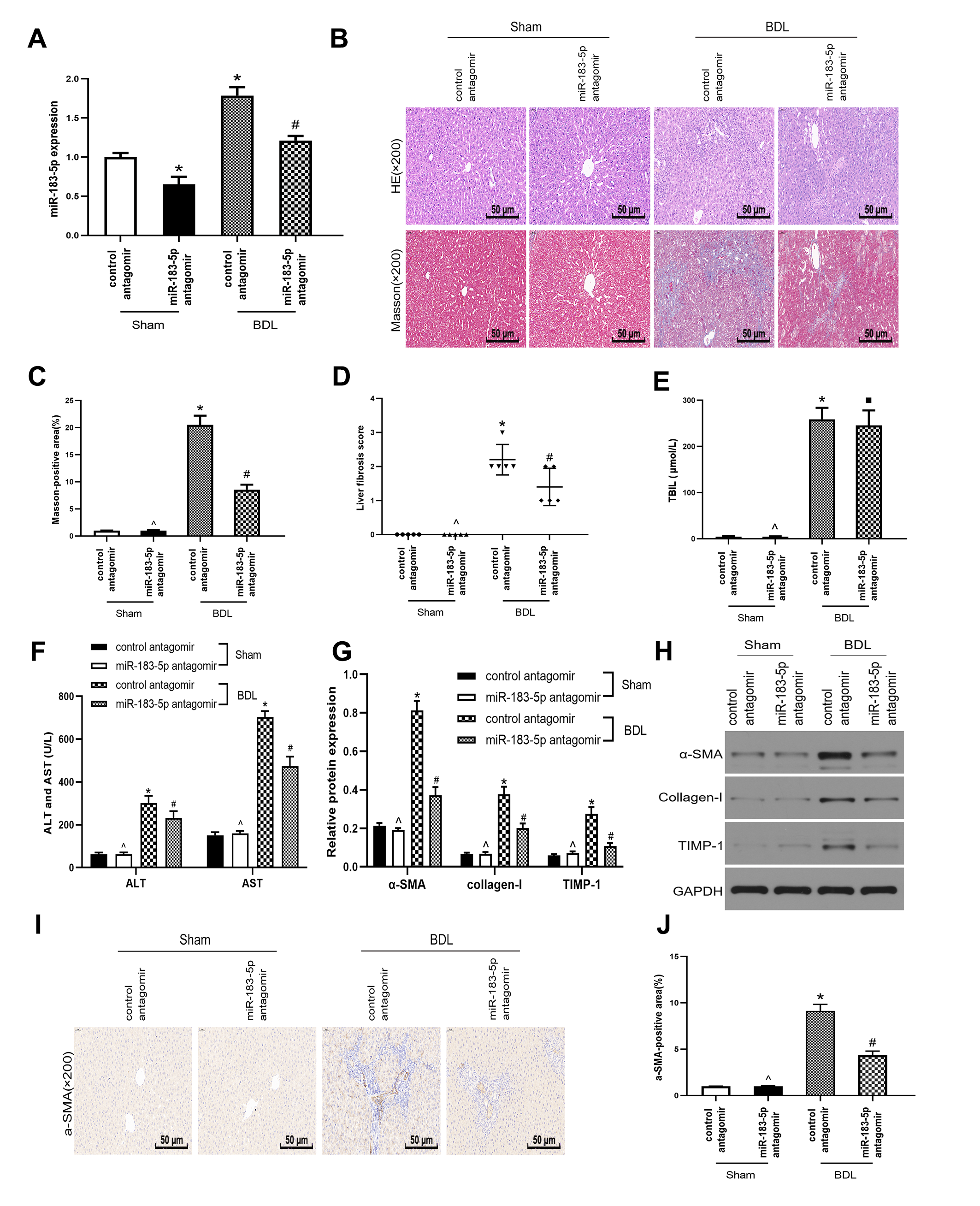

Supplement: Supplementary file 9 [file Data_Sheet_9.ZIP › Figure/Figure2.tif]

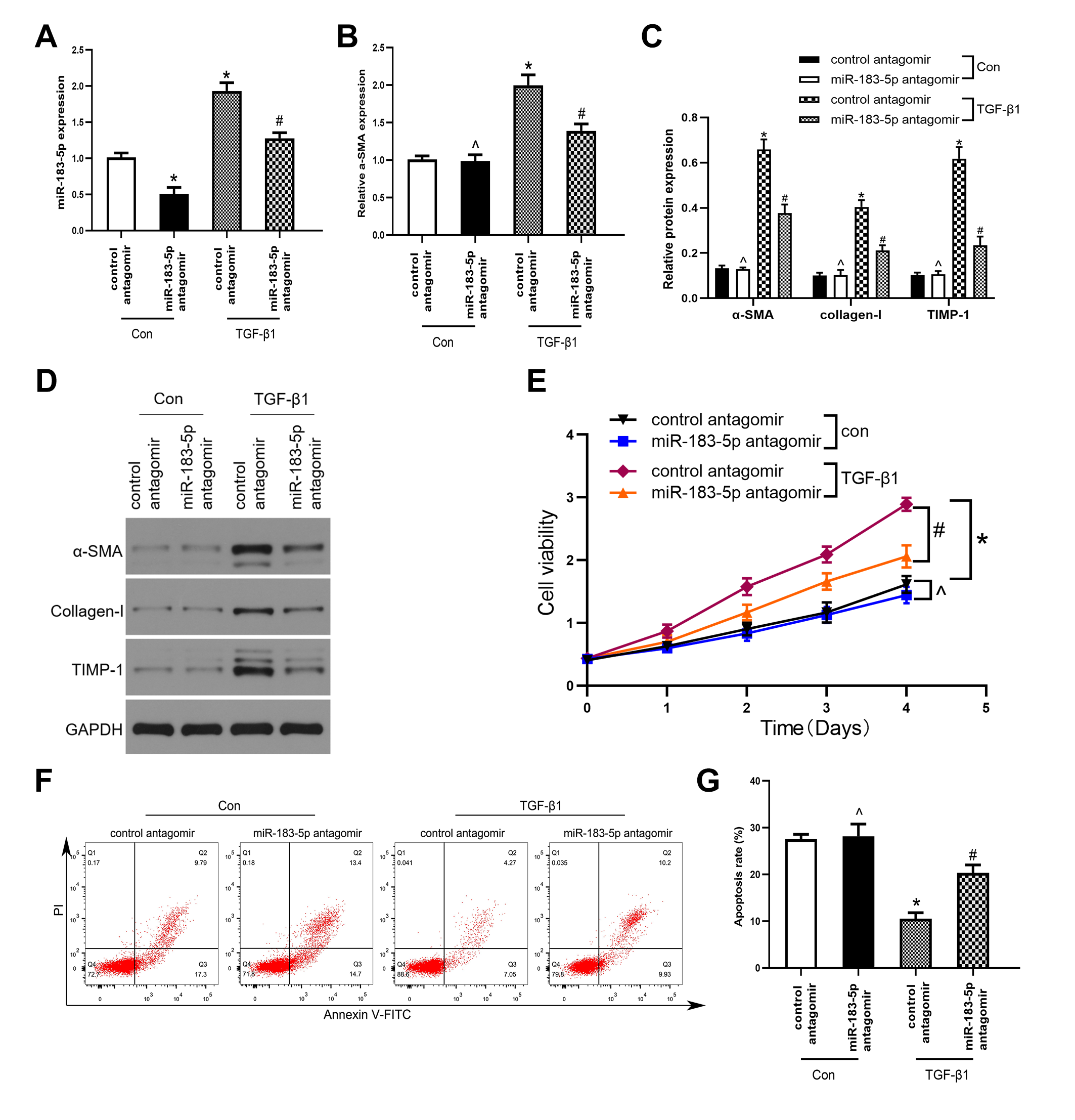

Supplement: Supplementary file 9 [file Data_Sheet_9.ZIP › Figure/Figure3.tif]

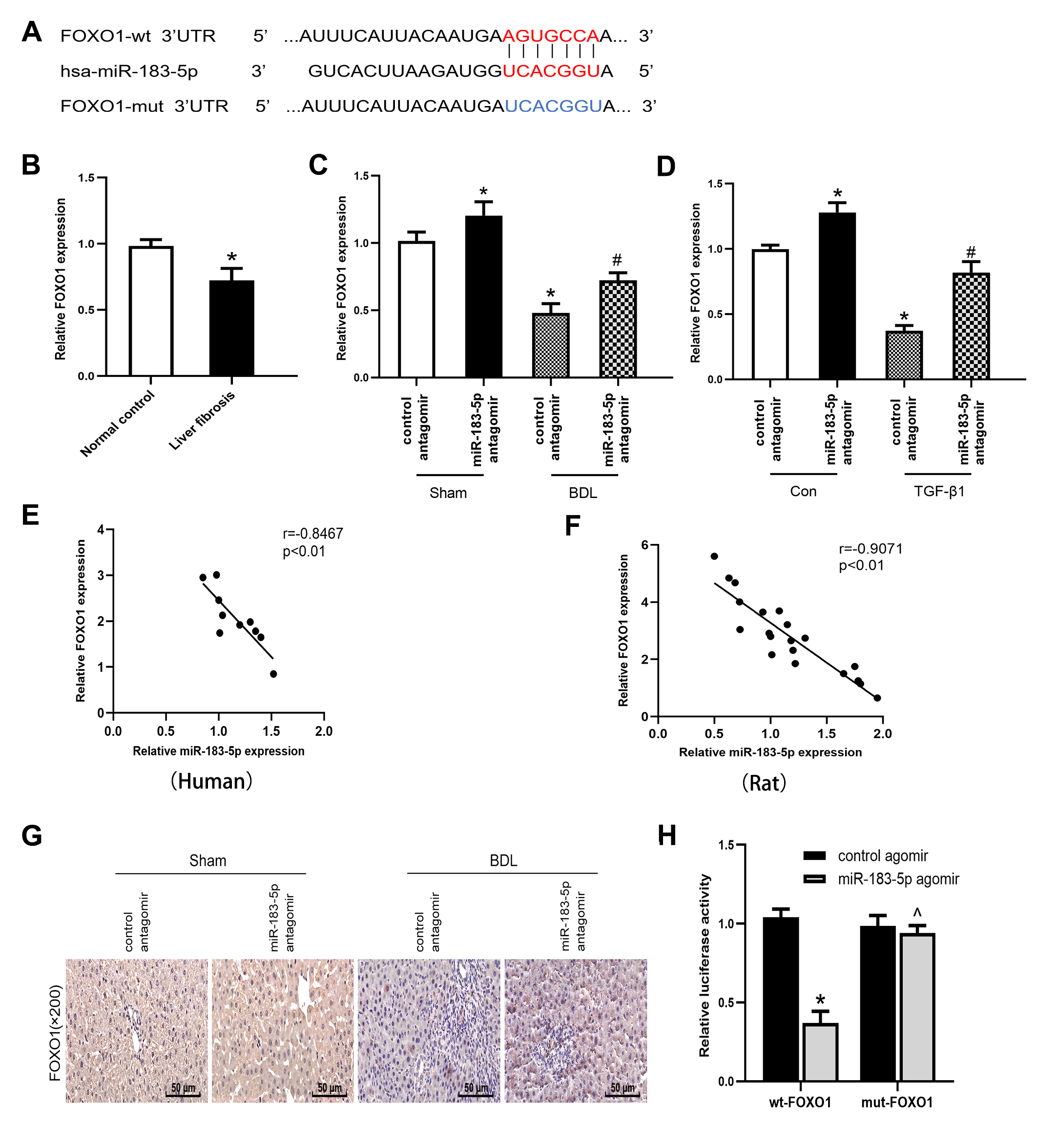

Supplement: Supplementary file 9 [file Data_Sheet_9.ZIP › Figure/Figure4.tif]

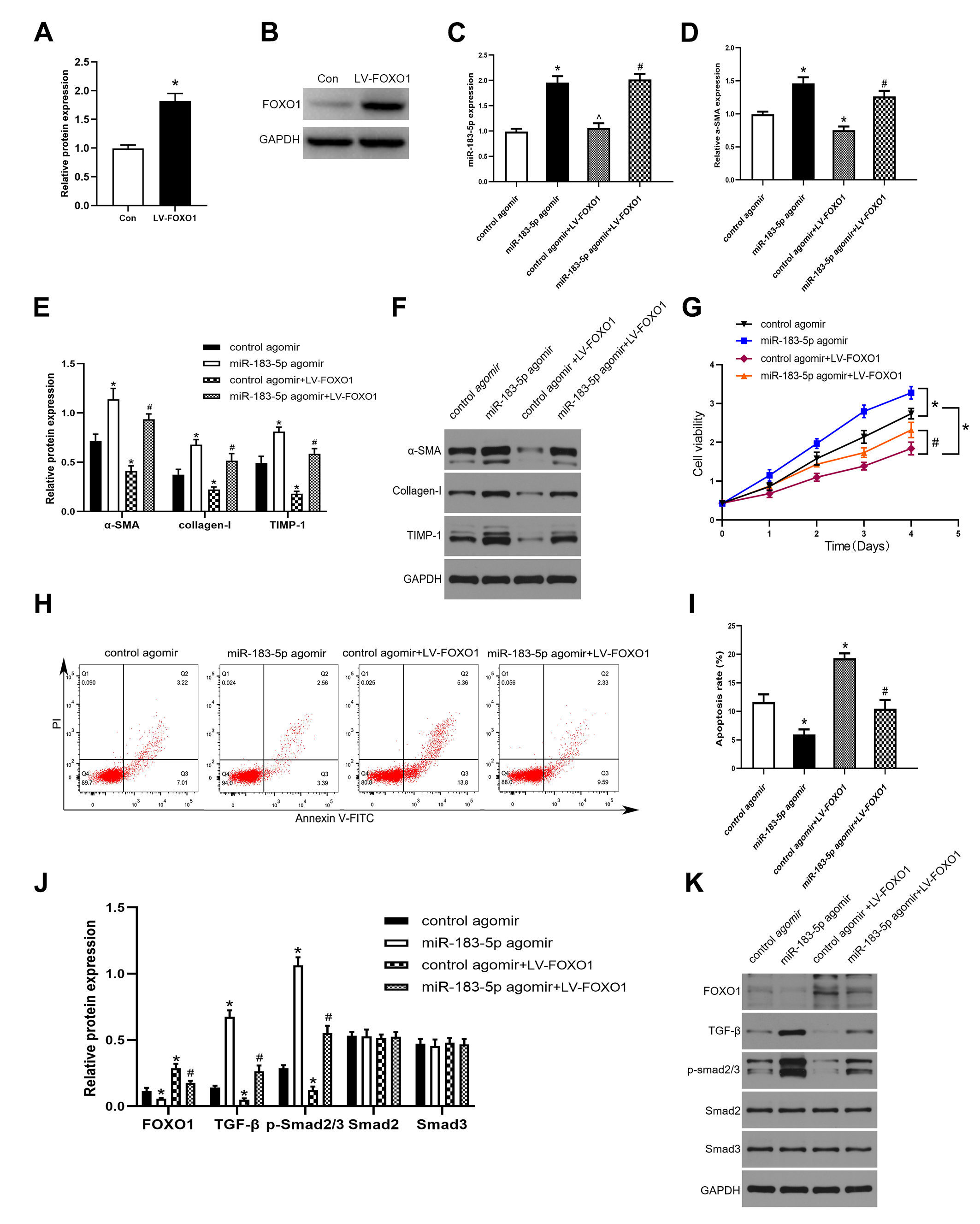

Supplement: Supplementary file 9 [file Data_Sheet_9.ZIP › Figure/Figure5.tif]

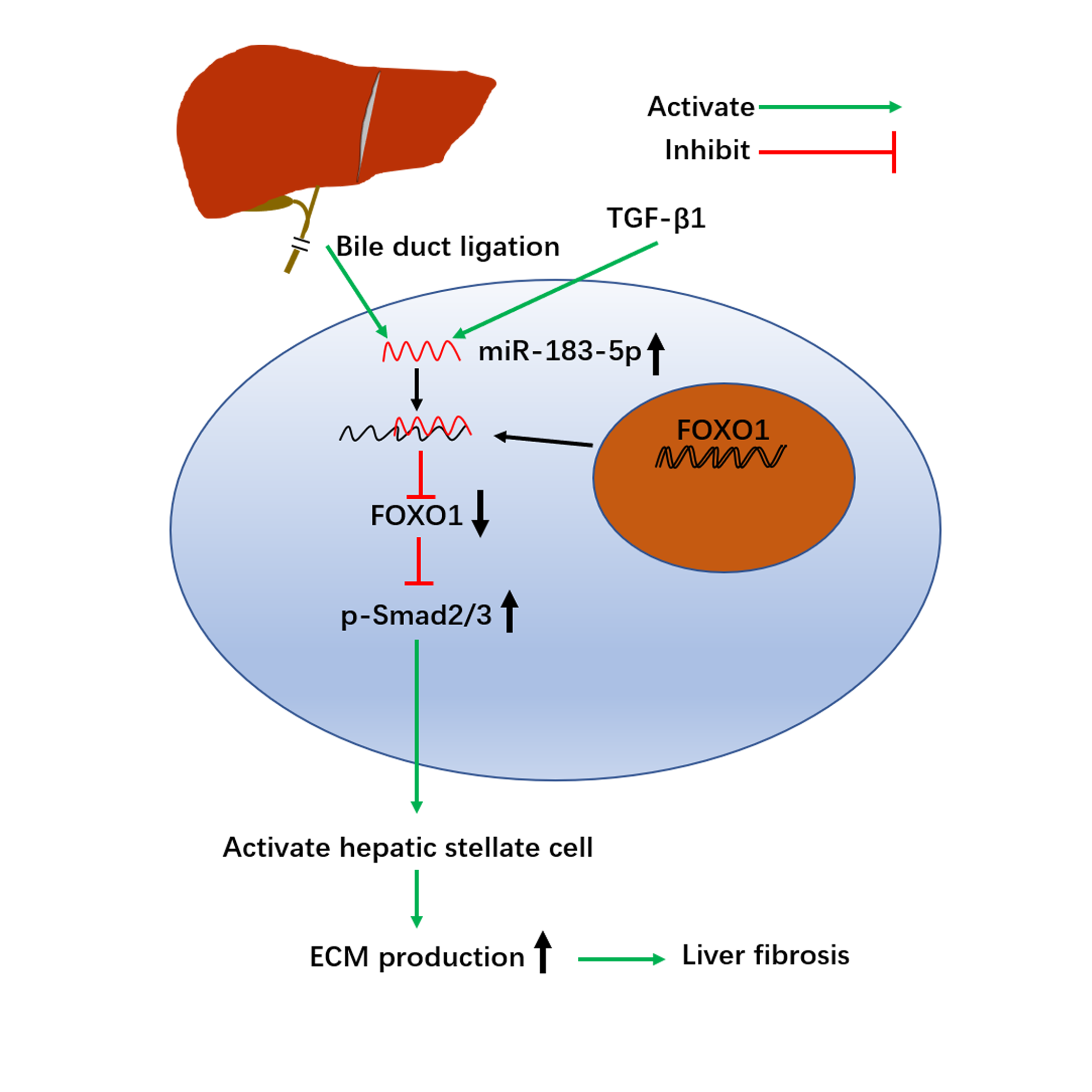

Supplement: Supplementary file 9 [file Data_Sheet_9.ZIP › Figure/Figure6.tif]
